# Supramolecular Coordination Cages for Artificial Photosynthesis and Synthetic Photocatalysis

**DOI:** 10.1021/acs.chemrev.2c00759

**Published:** 2023-01-20

**Authors:** Rens Ham, C. Jasslie Nielsen, Sonja Pullen, Joost N. H. Reek

**Affiliations:** Homogeneous and Supramolecular Catalysis, Van ’t Hoff Institute for Molecular Sciences, University of Amsterdam, 1098 XHAmsterdam, The Netherlands

## Abstract

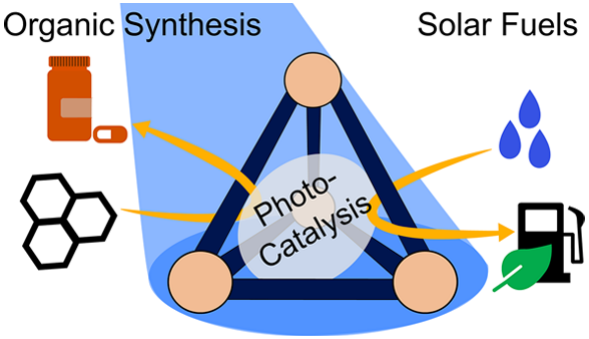

Because sunlight is the most abundant energy source on earth, it has huge potential for practical applications ranging from sustainable energy supply to light driven chemistry. From a chemical perspective, excited states generated by light make thermodynamically uphill reactions possible, which forms the basis for energy storage into fuels. In addition, with light, open-shell species can be generated which open up new reaction pathways in organic synthesis. Crucial are photosensitizers, which absorb light and transfer energy to substrates by various mechanisms, processes that highly depend on the distance between the molecules involved. Supramolecular coordination cages are well studied and synthetically accessible reaction vessels with single cavities for guest binding, ensuring close proximity of different components. Due to high modularity of their size, shape, and the nature of metal centers and ligands, cages are ideal platforms to exploit preorganization in photocatalysis. Herein we focus on the application of supramolecular cages for photocatalysis in artificial photosynthesis and in organic photo(redox) catalysis. Finally, a brief overview of immobilization strategies for supramolecular cages provides tools for implementing cages into devices. This review provides inspiration for future design of photocatalytic supramolecular host–guest systems and their application in producing solar fuels and complex organic molecules.

## 1 Introduction

Chemical photocatalysis in a broad context aims at using light as a green reagent for driving reactions in a sustainable fashion, thus contributing to combat climate change and to a sustainable economy.^1^ In this sense, light-driven chemical conversions could be used in two main directions: (1) for synthesizing building blocks that could be used to produce bulk and/or fine chemicals such as pharmaceuticals^2−4^ and (2) to produce solar fuels that can contribute to transforming the current global energy supply into one that is sustainable.^5^ Although there is strong interest in the application of both directions, a detailed mechanistic understanding of the photochemical processes is often still lacking and their application remains underexplored. Whereas both directions are very different in scale and reaction scope, their elemental (photochemical) steps are similar. In that regard, both photochemical research fields can benefit from each other’s insights and detailed understanding.

Much of this research has been inspired by natural photosynthesis, which is responsible for the global oxygen content as well as most of Earth’s energy stocks in the form of sugars and fossil fuels. Mimicking principles of natural photosynthesis is a key part in photocatalytic research to pursue light-driven reactions efficiently.^6^ Several proteins, cofactors, and metal-centers are involved in photosynthesis, all of them purposefully having their own function.^7^ Overall efficiency is achieved due to a high degree of spatial organization, cooperativity, and chemical logistics, i.e., providing the right components at the right place and time and matching energy levels. Due to the unique chemical environment that is induced by the protein around active sites, nature is able to achieve high control over the activity and selectivity of photosynthesis and many other chemical transformations. In short, increased local concentration, substrate preorganization, as well as cooperativity of several functions within the protein, make them excellent catalysts.^8^

**Figure 1:**
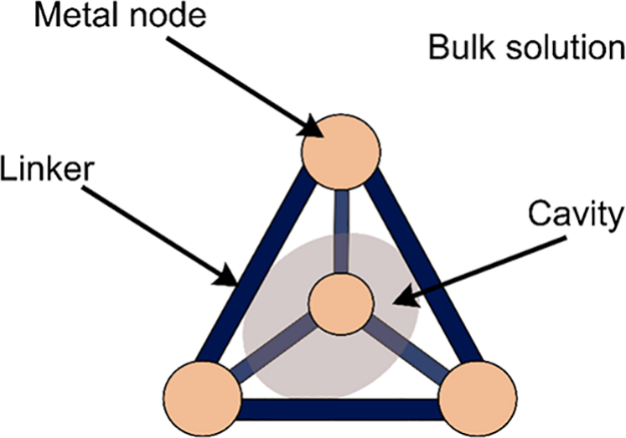
Schematic representation of a supramolecular coordination cage with different positions that are available for the introduction of (photo)catalytic functions.

Supramolecular self-assembly is a powerful strategy to build similarly complex and well-organized structures from simple building blocks, thus mimicking nature’s principles.^9,10^ Using metal ions and organic linkers, discrete coordination cages can be obtained (Figure 1).^11−22^ Coordination cages provide a single, defined cavity, which is easily accessible by substrates from bulk solution through dynamic exchange.^23,24^ Their homogeneous nature allows processing and mechanistic studies in solution. Coordination cages have extensively been used for enzyme-mimicking catalysis,^25−33^ making use of substrate preorganization, stabilization of reactive intermediates, and increased local concentration. For photocatalysis, redox-active moieties^34,35^ and light-absorbers such as organic dyes or metal-based photosensitizers need to be integrated,^36^ which has been recently reviewed.^37−40^ Additionally, other types of supramolecular assembly strategies used in chemical photocatalysis to achieve efficient photo(redox) reactions have been reviewed elsewhere.^41,42^

Herein we focus on coordination cages with application in light-driven catalysis. After introducing general principles, we will discuss photocatalysis using coordination cages within in two sections: (1) application of cages in the synthesis of solar fuels with the aim of storing (solar) light energy in molecules, and (2) application of cages in the synthesis of complex organic molecules. While the challenge in the first part mostly lies in managing multi-electron processes, the latter aims at enabling novel and sustainable synthetic strategies in the production of high-value chemicals. The sections will be structured according to the position of the photosensitizer (PS, in this review, the unit that absorbs the light) within the cage to generate the photocatalyst (in the review, the complete system needed for the light driven conversion). At the end, we will analyze which strategies lead to most successful generation of photoproducts and how such cages can potentially be integrated into devices.

## 2 General Principles

### 2.1 Artificial Photosynthesis

**Figure 2:**
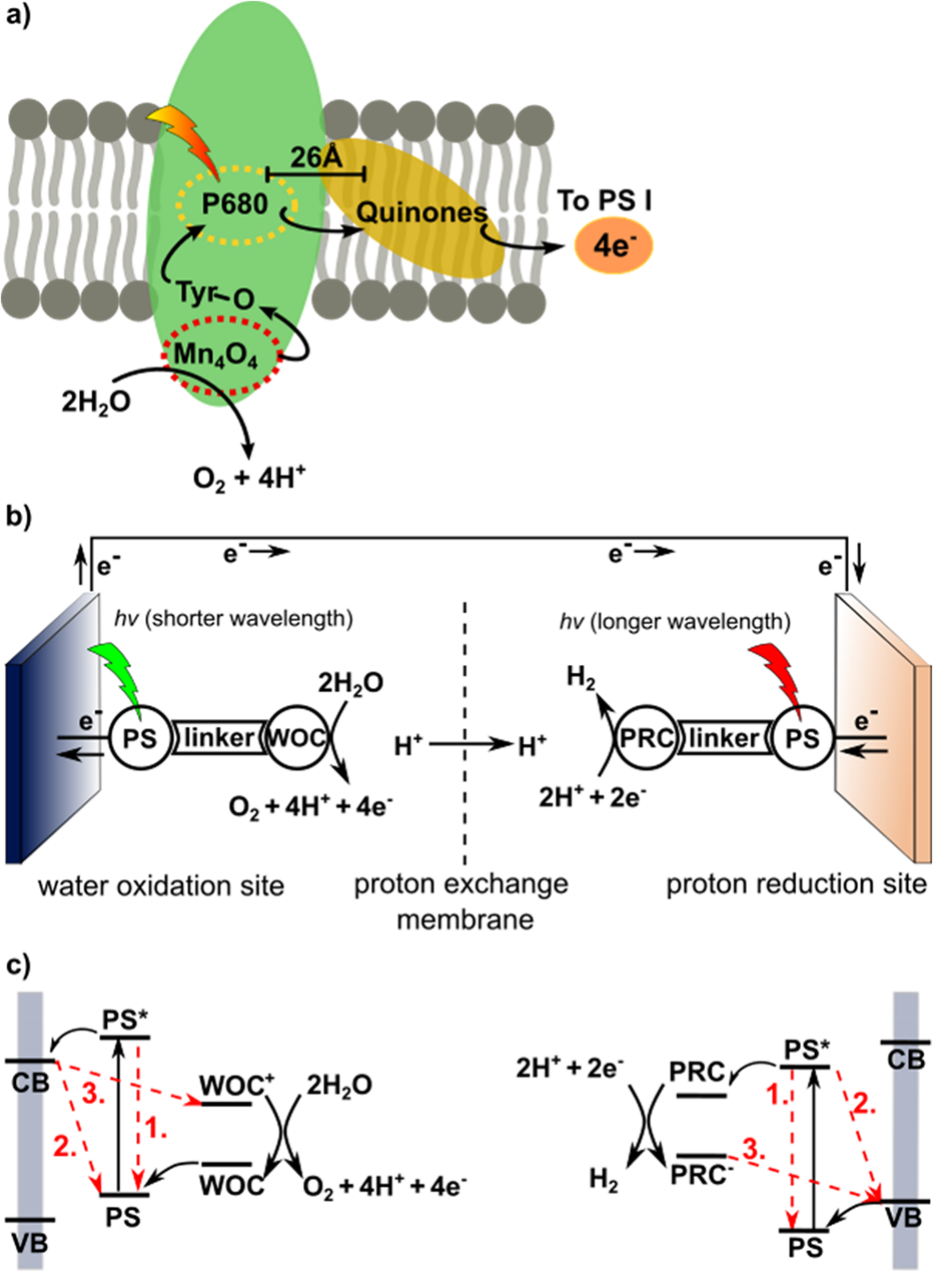
(a) Schematic representation of photosystem II, which catalyzes the oxidation of water to oxygen while charge separating protons and electrons. Flow of electrons is shown by the black arrows. (b) Schematic picture of an artificial photosynthetic device with an anode for light-driven water oxidation (PS = chromophore, WOC = catalyst) and a cathode for light-driven proton reduction catalysis (PS = chromophore, PRC = proton reduction catalyst). (c) Schematic representation of the relevant energy levels for light-driven water oxidation (left) and proton reduction (right) pathways unproductive charge recombination indicated in red (CB = conduction band, VB = valence band).

Artificial photosynthesis aims at the conversion of solar energy into chemical fuels, often referred to as *solar fuels*, by mimicking principles of natural photosynthesis. Three main functions are essential in natural photosynthesis, and these are the central features to be mimicked by artificial systems: (1) light-harvesting, (2) charge-separation, and (3) redox catalysis.^43^ Natural photosynthesis is a very complex process comprising multiple light and dark reactions, and the reaction centers are fostered by a manifold of cofactors. Water splitting takes place in photosystem II (PSII). Protons and electrons generated through light-driven water splitting are used to produce energy-rich ATP and NADPH, which are the chemical fuels used to drive consecutive dark reactions fixating carbon dioxide.^44^ Light-driven water splitting in PSII is achieved by the excitation of a distant chlorophyll (P680), which transfers the exciton to a quinone derivate about 26 Å away from the photosensitizer (Figure 2a).^7^ The oxidized photosensitizer then accepts electrons one-by-one from the catalytically active Mn_4_O_4_ cluster via a redox-active tyrosine residue. Spatial organization of the different components involved is crucial, as it minimizes unproductive reaction pathways while maximizing directed charge separation. Artificial photosynthesis simplifies these schemes: protons and electrons generated from water-splitting are directly used for the production of molecular hydrogen, carbon dioxide reduction, or for the reduction of other substrates. Likewise, water oxidation can in principle be replaced by the oxidation of organic matter, e.g., biomass,^45,46^ plastic waste,^47^ and small organic molecules.^48^

While the combination of both an oxidation reaction and a reduction reaction in one device or material is the ultimate goal of artificial photosynthesis, research toward the design and optimization of catalysts for energy conversion is typically focused on one of the half-reactions. This can be done either by chemical oxidation/reduction, by electrochemistry, or by light-driven catalysis using a photosensitizer (PS) and a sacrificial oxidant/reductant together with the catalyst. For instance, in light-driven proton reduction, a sacrificial reductant such as triethylamine (TEA) is used to supply the excited photosensitizer with electrons and to close the photoredox cycle. Similarly, in light-driven water oxidation, a sacrificial oxidant such as Na_2_S_2_O_8_ is applied.^49^ In a complete and functional system for solar-driven water splitting, both water oxidation and proton reduction (or alternative oxidation/reduction reactions) are coupled. For this, various designs are possible, including catalyst-PS colloids suspended in aqueous electrolyte.^50^ An alternative design more common in research laboratories and conceptionally similar to solar cells is the photoelectrochemical cell (PEC). The first example of such an artificial water splitting system generating molecular oxygen and hydrogen was described already in 1972 by Honda and Fujishima. This system was based on a TiO_2_ anode and a platinum cathode conducting water splitting under UV irradiation.^51^ Inspired by this pioneering study and by the design of dye-sensitized solar cells, dye-sensitized photoelectrochemical cells (DS-PECs) have been developed.^52^ A DS-PEC combines an oxidation reaction with a reduction reaction, possibly both driven by light (Figure 2b). The photocathode is made of a p-type semiconductor (e.g., NiO), on which molecular reduction catalysts and photosensitizers are immobilized. The photoanode is based on an n-type semiconductor (e.g., TiO_2_), functionalized with a molecular water oxidation catalyst (WOC) and a photosensitizer. Energy levels of the different components need be balanced carefully in order to obtain an overall driving force for the paired redox reactions. The overall light to fuel efficiency of a DS-PEC is determined by (1) light harvesting followed by charge separation (preventing charge recombination), (2) catalytic rates on each side, and (3) overpotentials. The overpotential is the extra potential added to the thermodynamic potential required to drive each reaction and is dependent on the catalyst used. In order to achieve efficient overall light-to-fuel conversion over a sufficiently long period of time, highly stable and fast catalysts are required. A major issue in DS-PECs is unproductive charge recombination, which can occur via different pathways: (1) decay of the excited electron at photosensitizer to the ground state before injection into the semiconductor, (2) charge recombination from semiconductor to the photosensitizer, and (3) regeneration of catalyst by semiconductor before catalysis occurs (Figure 2c). The difference in time scales of the various processes is often the reason for an inefficient interplay of the different components. Energy- and electron transfer should be guided by directionality, thus preventing unproductive side reactions such as back electron transfer (BET), leading to charge recombination. Ideally, forward electron transfer processes are faster than BET, and once the electron reaches the catalyst, it should be converted rapidly. Thus, ordering the components in a rational way ensures that the processes occur in a desired stepwise fashion. Supramolecular preassembly of different chromophores for instance leads to fast and directional electron transfer and thus long-distance charge separation in the light-harvesting part.^6^

### 2.2 Principles of Synthetic Photocatalysis

Nature uses light mainly for the generation of chemical fuels, i.e., NADPH and ATP, which are used to drive dark reactions creating complex organic matter. However, in organic synthesis, light can be used directly to drive multiple (catalytic) reactions relevant for chemical research and industry. The application of visible light is highly promising because it is abundant, clean, cheap, and safe. Furthermore, by using light, one-electron redox processes typically occur, introducing open-shell organic molecules as intermediates, which may lead to new reaction pathways. Crucially, a specific low-energy wavelength (i.e., visible light) should be used to activate only the PS without damaging other (organic) molecules in the reaction mixture. In the past two decades, research in light-driven catalysis for organic synthesis has been very fruitful.^41,53−55^

**Figure 3:**
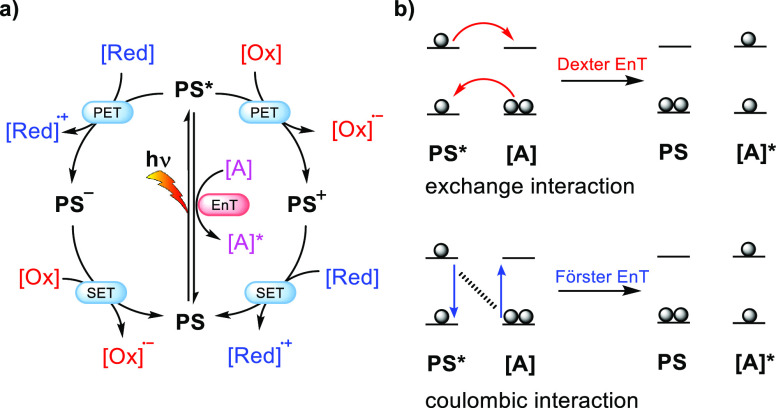
(a) General mechanisms of photo(redox)catalysis: energy transfer (EnT) and photoinduced electron transfer (PET) involving a photosensitizer (PS), single electron oxidants [Ox] and reductants [Red], and energy acceptor [A] that accepts excited-state energy. (b) Schematic representation of the energy levels for the Dexter (red) and Förster (blue) EnT processes from an excited-state PS.

In general, two different productive mechanisms can occur after excitation of the PS (Figure 3a): (i) energy transfer (EnT) from the excited state (PS*) to an energy acceptor ([A]), and (ii) photoinduced electron transfer (PET) to form either an oxidized (PS^+^) or reduced (PS^–^) species that is subsequently neutralized via single electron transfer (SET) to the original state.^55^ Visible light can therefore be used to activate (organic) molecules to overcome energy barriers and to drive redox reactions by enabling one-electron chemistry.^3,56−58^ Several catalysts for EnT and photoredox reactions have been developed, ranging from metal complexes to organic dyes. Photoredox catalysis typically proceeds via PET to or from a sacrificial agent such as TEA or persulfate after initial excitation of the photosensitizer. EnT catalysis involves the transfer of the excited-state energy from the PS to an energy acceptor (Figure 3b). Generally, two types of nonradiative EnT mechanisms can occur: Dexter energy transfer (DET) or Förster resonance energy transfer (FRET). Crucially, both significantly rely on the distance between the energy donor and acceptor, which can be controlled by supramolecular preorganization.^59^

Each excited state has a limited lifetime (τ) before emissive and vibrational relaxation pathways result in a return to its ground state. In order to undergo energy or electron transfer from the excited state, the reaction partner (i.e., catalyst or substrate) has to be in proximity to PS*. In solution, these processes are limited by diffusion and are highly dependent on the concentrations of the solutes. Therefore, the excited-state lifetime of the photosensitizer is mainly determining the viability of the overall process. Generally the excited-state lifetime should be τ > ns to ensure energy or electron transfer to a catalyst or substrate. However, if photosensitizer, catalyst, and/or substrate are preorganized, the system is no longer limited by diffusion, and shorter lifetimes could become feasible. In addition, BET processes are often faster than a chemical reaction and thus are challenging the turnover. In natural (photo)systems, electrons are transferred through multiple units far away from the PS by making use of charge transfer linkers, which is an elegant strategy to combat BET processes.^7^ In simplified artificial systems like most synthetic supramolecular photocatalysts, it is thus key to consider that the desired forward (electron transfer) process is faster than the backward one. Interestingly, in the area of photoredox catalysis, there is little attention for solar to product efficiency as the focus is typically on getting new products in high yields.

### 2.3 Supramolecular Coordination Cages

Supramolecular architectures based on coordination complexes have been extensively explored and have been demonstrated to display excellent properties for biomimetic catalysis.^60−63^ A coordination complex is build up from a metal ion and ligands (Figure 1). Depending on the type of metal, its oxidation state, and the structure of the ligands, specific structures can be made via self-assembly.^64^ Ligands containing two or more donor groups are able to coordinate multiple metals, leading to larger self-assembled objects. Depending on the angle between these donor groups, determined by the ligand backbones, and the coordination geometry of the metal, either discrete coordination cages or coordination polymers like metal–organic frameworks (MOFs) can be made.^11,65^ Besides using multidentate bent ligands,^11,66^ metal ions and clusters are used as nodes, and these can be capped^67^ or protected by bis-dentate ligands^68^ in order to selectively obtain discrete coordination cages. In addition to highly symmetric, homoleptic cages with one type of ligand, in recent years, a synthetic toolbox for the self-assembly of heteroleptic cages with increasing complexity has been developed.^69^ In contrast to MOFs, coordination cages are soluble and feature a single, well-defined cavity, providing a chemical environment that can be tailored similarly to enzyme pockets. The chemical environment in these cavities can be tuned by the nature of ligands, (endohedral) functional groups attached to the ligands, as well as the charge that is induced by the metal connecting nodes. Additionally, computational work toward the prediction of self-assembly, binding, and catalysis within coordination cages has recently gained attention.^70,71^ Small molecules (such as substrates, intermediates, and catalysts) can be selectively bound within the cavity.^72,73^

**Figure 4:**
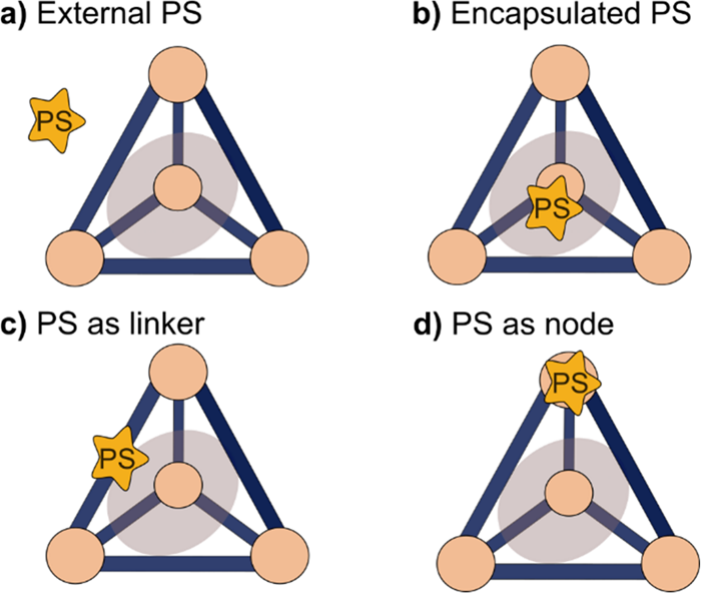
Schematic representation of light-absorbing supramolecular coordination cages with the photosensitizer (PS) at different positions (a) in bulk solution, (b) encapsulated inside the cage, (c) as part of the cage linker, and (d) as part of the cage metal node.

Supramolecular coordination cages have been extensively researched in various kinds of chemical transformations in which their porosity is exploited to preorganize substrates in the accessible cavity.^25−27^ Because the cavity is shielded from the bulk solution, two different environments are created that are separated from each other (Figure 1). This allows for rational design of the cage structure to place desired functionalities outside or inside the cage, which can be used to induce charge separation. In addition, bifunctional cages can incorporate both a PS and the desired excited-state quencher, e.g., substrate or catalyst, to remove diffusion constraints of the PET. In such a system, the use of photosensitizers with shorter lifetimes can be realized. Generally, the PS can be installed in four different places (Figure 4): in the bulk solution, encapsulated inside of the cavity, in the linkers forming the cage, and in its metal nodes. In summary, coordination cages show a high potential for increasing efficiency and scope of photochemical processes, mainly due to the unique constraint environment they provide.

## 3 Coordination Cages for Artificial Photosynthesis

In the following section, the focus lies on coordination cages that have been used for artificial photosynthesis, e.g., the generation of solar fuels. We will discuss various approaches in which light-harvesting, charge separation, and catalysis have been combined in supramolecular coordination cages. So far, such systems have been mostlty used for either the oxidation or the reduction reaction and examples of the combination are rare. In the following, we first describe the relevant reactions before discussing how coordination cages have been used in this context.

### 3.1 Reactions

#### 3.1.1 Water Oxidation

**Figure 5:**
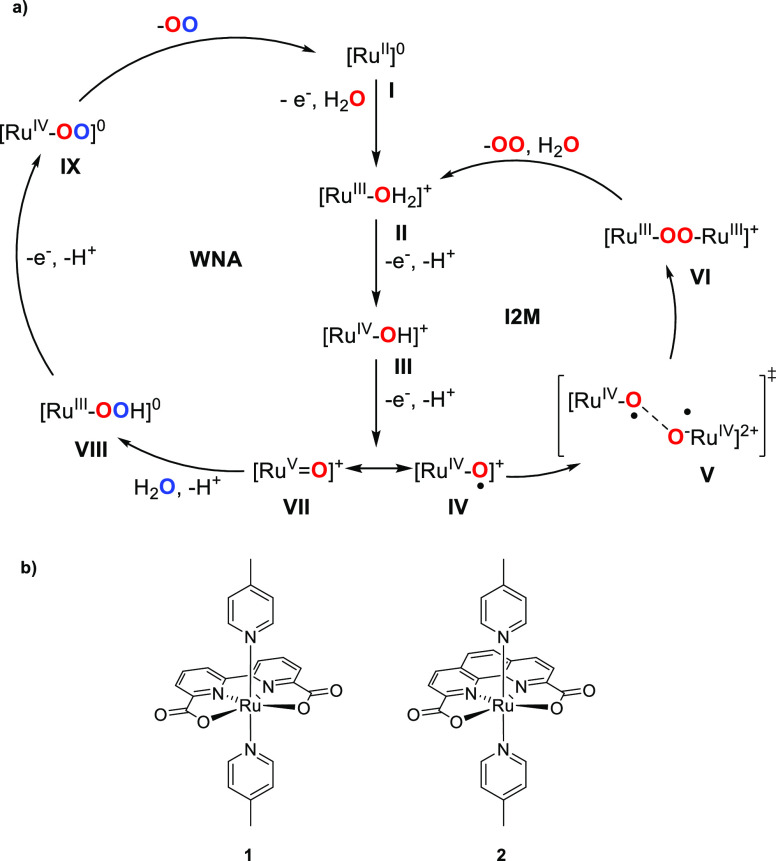
(a) Comparison of the two mechanisms WNA (left) and I2M (right) on the example of (b) Sun’s catalysts [Ru(pda)(pic)_2_] (**1**) and [Ru(bpa)(pic)_2_] (**2**).^80^

Water oxidation is a four-electron process generating molecular oxygen, protons, and electrons (eq 1). Because this reaction proceeds typically with slow kinetics, it represents one of the bottlenecks in artificial photosynthesis.^74^ In the past decades, multiple molecular and heterogeneous catalysts have been developed that partially overcome some of the limitations by lowering the overpotential and displaying fast kinetics.^75^
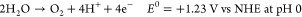
1One of the best molecular water oxidation catalysts to date, [Ru(bpa)L_2_] (**1**, bpa = 2,2′-bipyridine-6,6′-dicarboxylic acid), has been developed by the group of Sun.^76^ The seven-coordinate configuration of this ruthenium-based catalyst allows the formation of higher oxidation states (up to Ru^V^) of the metal center via proton-coupled electron transfer (PCET) processes. In combination with Ce(IV) as chemical oxidant, this catalyst features similar activities to PSII.^76−78^ As described in eq 1, two water molecules are required to form molecular oxygen, and the key step in the overall reaction is the O–O bond formation. For this, two mechanistic pathways have been identified, depending on the nature of catalyst and the reaction conditions: (1) water nucleophilic attack (WNA) and (2) oxyl-radical mechanism (I2M), also referred to as an intermolecular face-to-face mechanism (Figure 5).^79^

Both mechanisms initially follow a similar pathway: Ru(II) (**I**) is oxidized to Ru(III), followed by coordination of a water molecule to form intermediate **II**. This is followed by two consecutive PCET steps via intermediate **III**, abstracting in total two electrons and two protons to generate either a Ru(V) oxo (**VII**) intermediate which will follow the WNA mechanism, or a Ru(IV) oxygen radical (**IV**) intermediate that will follow the I2M mechanism. In the I2M mechanism, two Ru(IV) oxygen radicals combine (**V**) to produce intermediate Ru(III)-O-O-Ru(III) (**VI**). From here, oxygen is released and coordination of H_2_O regenerates intermediate **II**. On the other hand, during the WNA mechanism nucleophilic attack of H_2_O on intermediate **VII** takes place, resulting in generation of a Ru(III)-OOH peroxide (**VIII**) intermediate while releasing H^+^. Finally, PCET leads to the peroxide radical **IX**, which releases oxygen and regenerates starting complex **I**. Sun’s water oxidation catalyst **1** follows the bimolecular I2M mechanism, while the slightly different catalyst [Ru(pda)(pic)_2_] (**2**, pda = phenanthroline-2,9-dicarboxylicacid, pic = 4-picoline) follows the WNA mechanism.^77,80^ While molecular catalysts following the I2M mechanism typically feature lower overpotentials, the WNA mechanism has so far resulted in the highest catalytic rates.^81,82^ Based on its high activity and stability as molecular catalyst, Sun’s water oxidation catalyst **1** and its derivatives have become some of the most used water oxidation catalysts.^83−85^

#### 3.1.2 Proton Reduction

The second key reaction in artificial photosynthesis is the generation of molecular hydrogen from protons. Per definition, the reduction potential of the proton reduction reaction is 0 V vs NHE at pH = 0 (eq 2). As these thermodynamic potentials scale with pH, water reduction at neutral or basic pH occurs at a lower potential (eq 3), while the water oxidation requires less potential under these conditions.^86,87^ While hydrogen can be generated electrochemically, for example on Pt electrodes, numerous homogeneous systems for light-driven proton reduction have been reported (e.g., natural hydrogenase mimics).^88−94^
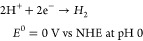
2
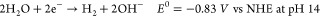
3

#### 3.1.3 CO_2_ Reduction

Carbon dioxide is the most common greenhouse gas in the atmosphere that plays a significant role in global warming by the greenhouse effect.^95^ In order to close the carbon cycle, novel and sustainable approaches are required to convert CO_2_ into other chemicals such as CO and HCOOH, which can be used to generate fuel or as building blocks for the chemical industry. Below the two reactions and reduction potentials are given for CO_2_ reduction into CO and HCOOH, eqs 4 and 5, respectively.^96^
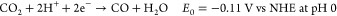
4
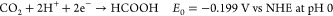
5Lately, substantial progress in light-driven CO_2_ reduction to CO and formic acid has been made,^97−99^ however homogeneous (photo)catalysts have shown low stabilities because they are readily transformed to nonactive forms during prolonged light-driven reactions.^100^ As a potential solution, these homogeneous (photo)catalysts can be incorporated within cages, resulting in more stable catalysts while featuring high catalytic activity and selectivity. Another challenge is that the reduction potentials required are close to that for proton reduction, thus the reduction of CO_2_ competes with the proton reduction reaction.^101^

#### 3.1.4 H_2_S Oxidation

H_2_S is abundant in natural gas and crude oil, which despite the toxicity of the gas has resulted in increased interest as feedstock.^102^ In analogy to water splitting, H_2_ can be generated from photocatalytic H_2_S splitting, forming elemental sulfur as a side product.^103,104^ This overall reaction, sulfide oxidation with proton reduction, is displayed in eqs 6 and 2, respectively.^105^
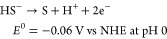
6As has been discussed in section 2 of this review, functionalities can be generally introduced into coordination cages in four different ways (Figure 4): The PS can be (1) external in solution, (2) encapsulated in the cavity, (3) installed as linker, or (4) introduced as metal node. In the following discussion, we divide recent literature into subsections according to the position of the PS and by the investigated redox reaction.

### 3.2 Cage Catalysts with External Photosensitizer

In this part, we discuss literature where the catalyst is part of the supramolecular structure and an external photosensitizer is used.

#### 3.2.1 Water Oxidation

**Figure 6:**
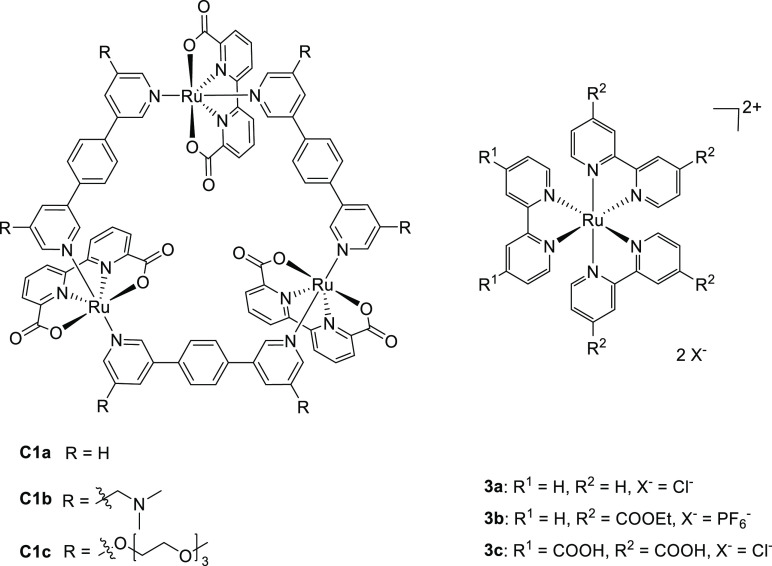
Structures of supramolecular triangles (**C1a**–**c**) and typical Ru(bpy)_3_ derivates (**3a**–**c**) used by the group of Würthner. Each triangle contains three Ru-based WOC catalysts.

Würthner and co-workers incorporated [Ru(bpa)(L)_2_] complex **1** as metal node in a series of supramolecular rings (Figure 6). In a first report, ditopic pyridine ligands were used to prepare supramolecular triangle **C1a**, containing three Ru(bpa) catalytic centers.^106^ The catalytic activity of the new supramolecular triangles was initially compared to the parent complex **1** by using cerium(IV) ammonium nitrate (CAN) as chemical oxidant. Due to their low solubility in pure water, solvent mixtures of MeCN in H_2_O were used, with 59% MeCN showing the highest catalytic activity. The TON and TOF obtained when the triangles are used are 7400 and 155 s^–1^, respectively. Under the same conditions, the monomeric reference catalyst **1** showed only a TON and TOF of 970 and 8.4 s^–1^, respectively. For homogeneous photocatalytic experiments, Ru(bpy)_3_Cl_2_ (**3a**) was used as photosensitizer with Na_2_S_2_O_8_ as a sacrificial oxidant.

Embedding the catalyst in the supramolecular triangle showed two effects. Firstly, the stability of the molecular catalyst was increased, leading to higher TON. This is due to the tethering effect of the connecting ligand in the triangle, avoiding decomposition of the catalyst. In the mononuclear control complex **1**, dissociation of one pyridine ligand leads to decomposition of the catalyst, and such dissociation is prohibited in the triangle structure. Secondly, the oxidation potentials increase for the triangles by ±100 mV per formal oxidation event from Ru^II^ to Ru^V^ compared to the monomer, possibly due to the close proximity of the catalytic centers.

Embedding the catalyst in the supramolecular triangle leads to a change in mechanism, as revealed by kinetic studies and ^18^O labeling experiments. While the molecular reference complex **1** typically follows the I2M mechanism, triangle **C1a** performs water oxidation via the WNA mechanism. Experiments revealed that the process follows first-order reaction kinetics in the oxygen evolution reaction for both catalyst and CAN concentration.^109,110^ Furthermore, multiple water molecules are preorganized within the supramolecular structure. DFT calculations revealed that up to 10 water molecules can fit in the cavity of the triangle, while only three are required to form a hydrogen bonding network between two Ru–OH centers. Due to preorganization of the water molecules, the activation energy for O–O bond formation is lowered and as a result the overall activity is significantly increased when using the triangle as catalyst.

In a follow-up study by Würthner and co-workers, two derivatives of **C1a** were synthesized, bearing water-solubilizing groups in the backbone of the supramolecular triangle in order to increase the water solubility of the WOC.^107^**C1b** was synthesized containing tertiary amines in the backbone structure of the ligand, being able to perform chemical water oxidation with CAN in a solvent mixture of 7:3 MeCN:H_2_O, obtaining a TOF up to 147 s^–1^ and TON up to 5.2 × 10^3^ at pH = 1. **C1c**, bearing oligo(ethylene glycol) functionalities on the backbone, was also active in fully aqueous solution, however, the photocatalytic activity decreased by ca. 20%. On the other hand, lowering the MeCN content for **C1b** led to precipitation of the catalyst. The lower activity observed for **C1c** is likely due to Coulombic repulsion between the charge on the catalyst and the charged Ce^IV^ ions. Overall, the more water-soluble supramolecular triangles displayed relatively high activities yet did not outperform the parent triangle **C1a** in terms of activity. This was attributed to the new supramolecular triangle being prone to oxidative decomposition.

The same group also investigated the effect on water oxidation by substituting the bpy backbone of the photosensitizer with electron-withdrawing groups (**3b** and **3c**) to increase the PS^+^/PS oxidation potentials and, therefore, the thermodynamic driving force for activation of the WOC.^108−111^ The photosensitizers were compared using catalyst **C1a** and the more water-soluble catalyst **3c** in the presence of sodium persulfate as a sacrificial electron acceptor. Unexpectedly, the highest catalytic activity for **C1a** was observed in combination with **3a**, obtaining a TOF up to 10.9 s^–1^ and TON up to 430 in a 1:1 MeCN:H_2_O (v/v) solvent mixture, while using **3b** and **3c**, the TOFs decreased to 2.8 and 0.5 s^–1^, respectively. A similar trend was obtained for **C1b** in combination with the photosensitizers, obtaining TOFs of 9.5, 2.2, and 0.4 s^–1^ for **3a**, **3b**, and **3c**, respectively. Photocatalytic water oxidation using catalyst **C1b** was also explored in a 5:95 MeCN:H_2_O solvent mixture, as this WOC is more soluble in water than its **C1a** derivative. Interestingly, the highest catalytic activity was observed when **3b** was used as sensitizer, obtaining a TOF of 10.8 s^–1^ and a TON of 320, compared to TOFs of 2.9 and 0.7 s^–1^ for **3a** and **3c**, respectively. It is notable that a 7.5 times lower PS concentration was used in the 5% MeCN mixture due to limited solubility of the photosensitizer in the respective solvent mixture. In comparison, in the 1:1 MeCN:H_2_O mixture, **3b** in combination with **C1b** decreased the TOF and TON to 1.1 s^–1^ and 45 at 0.2 mM **3b** concentration, respectively, indicating high efficiency in the 5:95 MeCN:H_2_O system for the relevant photosensitizer concentration. Nanosecond flash photolysis was used to study the efficiency of electron transfer from **C1b** to the photosensitizers. The electron transfer rate *k*_ET_ was observed to be 1 order of magnitude larger for **3b** compared to **3a**. In addition, Stern–Volmer quenching studies revealed an emission quenching *k*_q_ for **3a** of one magnitude higher than **3b** in both 5:95 and 1:1 MeCN:H_2_O mixtures. This indicates that electron transfer from the WOC to the photosensitizer is not the rate-determining step in the 1:1 MeCN:H_2_O mixture. The low photocatalytic activity of both catalysts in combination with **3c** is explained by electrostatic repulsion of anionic carboxylates on the PS and the negative charge of the persulfate anions, which could improve upon performing the reaction in stronger acidic conditions.

**Figure 7:**
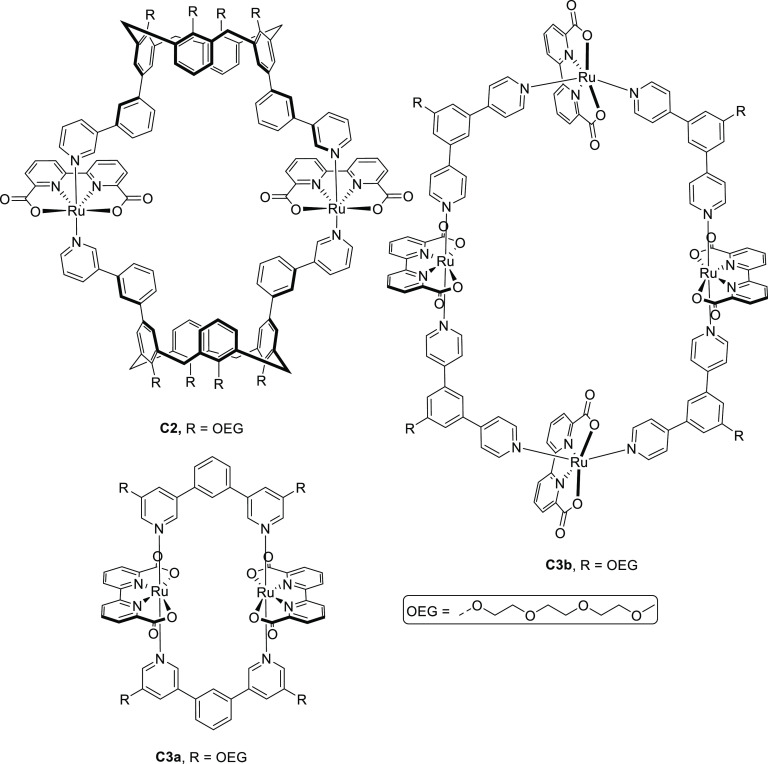
Structures of supramolecular ring structures based on Ru nodes that are active in water oxidation catalysis containing oligo-ethylene glycol chains for improved solubility in aqueous media.

The synthesis of a cyclic dinuclear Ru(bda) complex equipped with oligo(ethylene glycol)-functionalized (OEG) axial calix[4]arene ligands for homogeneous catalytic water oxidation under highly diluted conditions (**C2**, see Figure 7) was reported by the same group.^112^ Performing photocatalytic water oxidation using **C2** (0.24 mM) as catalyst led to TOF of 15.5 s^–1^ and TON of 460 in a 40% MeCN in phosphate buffer (pH = 7). Increase of the MeCN in the solvent mixture led to a decrease of the TOF due to competitive binding of MeCN to the catalytic center (13.3 s^–1^ in 1:1 MeCN:H_2_O), yet the TON did increase slightly (540 in 1:1 MeCN:H_2_O) because the supramolecular structure is more stable in solutions with a higher MeCN content.

Würthner and co-workers improved the system even further by synthesizing di-, and tetranuclear Ru(bda) OEG-functionalized supramolecular rings (**C3a** and **C3b**, see Figure 7) based on previously mentioned triangle (**C1c**).^113^ OEG side groups were incorporated in the structure to increase the water-solubility of the catalysts, enabling all of the catalysts to perform photocatalytic water oxidation in a 1:1 MeCN:H_2_O mixture. Photocatalytic experiments revealed that upon increasing the size and number of catalytic centers of the supramolecular ring, the activity of photocatalytic water oxidation increases. Using **C3b** as catalyst resulted in a TOF of 23 s^–1^ and TON of 500, while the smaller rings obtained TONs of 400 and 36 for **C1b** and **C3a**, respectively. In addition, not only did the total TOF increase but the TOF per Ru^II^-center also increased, ranging from 5.8 s^–1^/Ru for the tetramer, compared to 3.3 s^–1^/Ru for the trimer. Because for all catalysts similar oxidation potentials were measured, the increase in photocatalytic performance is suggested to be a result of higher rates by water pre-organization increased stability by the macrocyclic effect (in which case not all the ruthenium in solution is active).

**Figure 8:**
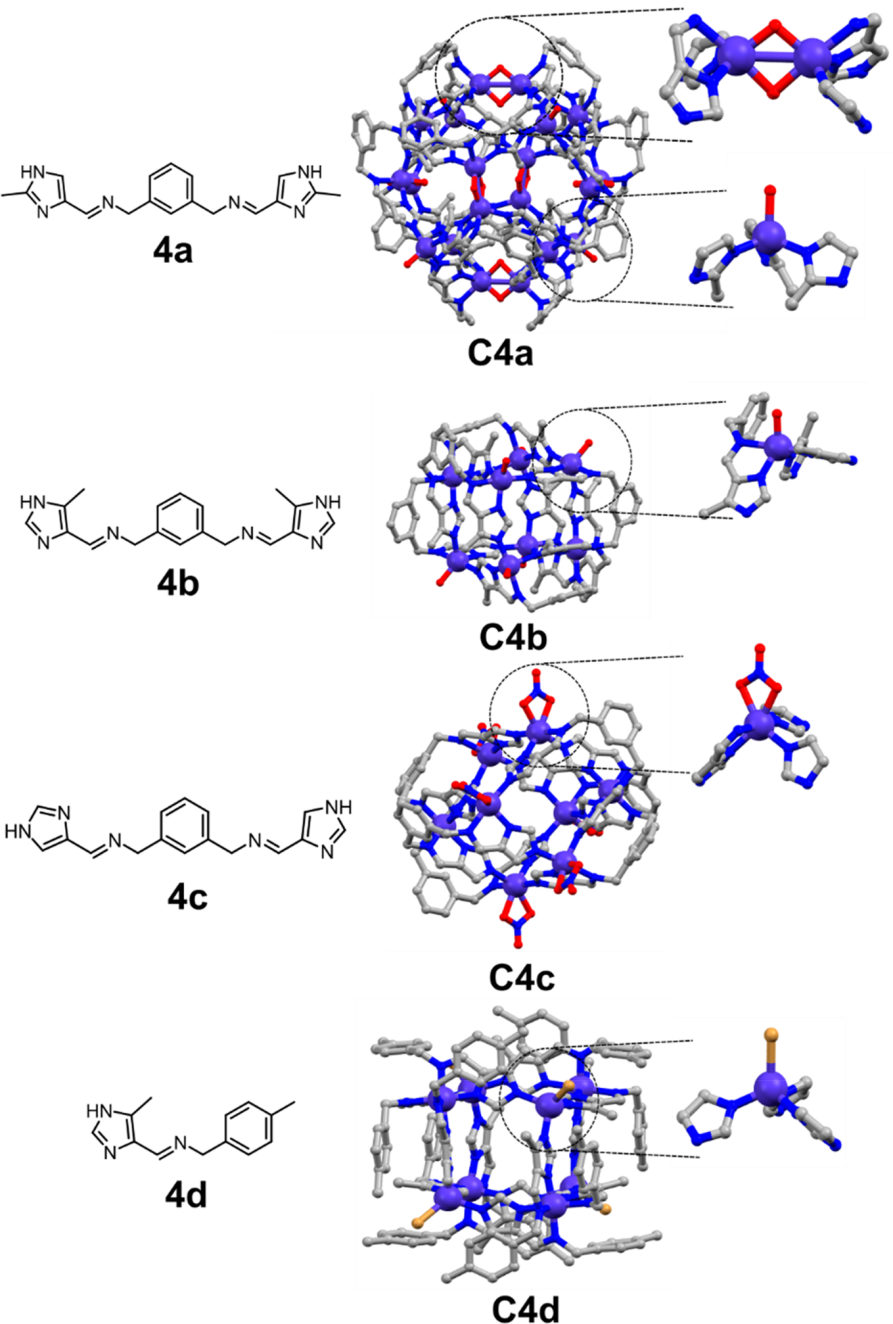
Different imidazolate ligands **4a**–**d** and XRD structures of their respective cobalt cages **C4a**–**d** for water oxidation developed by the group of Li. The magnified coordination environment of the Co center plays an important role in catalysis. Atoms: C = gray, N = blue, O = red, Co = purple, Br = light brown.^114−116^

The first supramolecular cage for photocatalytic water oxidation was synthesized by Li and co-workers, [Co_20_(**4**)_12_(OH)_12_(H_2_O)_4_(ClO_4_)_8_] (**C4a**), bearing Co–O active sites and imidazolate ligand **4a** (Figure 8).^117^ Interestingly, the cage contained two different active sites: (i) bis(μ-oxo) Co–O–Co and (ii) single Co–O functionalities. In photocatalytic water oxidation experiments, a TOF of 7.5 × 10^–3^ s^–1^ was obtained using **3a** as photosensitizer and Na_2_S_2_O_8_ as a sacrificial oxidant at pH = 9. To evaluate the efficiency of the respective active sites individually, similar cages with different ligands (**4b**–**d**) were synthesized. [Co_8_(**5**)_6_(H_2_O)_6_]*(BF_4_)_6_ (**C4b**) contains only Co–O active sites, while [Co_8_(**6**)_6_(H_2_O)_6_](NO_3_)_6_ (**C4c**) and [Co^III^_4_Co^II^_4_(**7**)_12_]Br_4_ (**C 4d**) feature only interactions of cobalt ions with their respective counterions (NO_3_^–^ and Br^–^). For **C4b** and **C4c**, only 50% and 25% of the activity was obtained, respectively, while for **C4d**, no oxygen evolution was observed at all. The enhanced activity of **C4c** over **C4d** could be attributed to more feasible substitution of the NO_3_^–^ counterion acting as ligand with H_2_O, compared to the Br^–^ counterion. The higher activity of **C4b** over **C4c** and **C4d** is attributed to the Co–O active metal centers. Mechanistic studies revealed that water oxidation in the bis-cobalt site is facilitated by PCET in the rate-determining step, explaining the higher oxygen evolution rates using **C4a** as a catalyst compared the **C4b**, where the bridged bis-cobalt is not present.

#### 3.2.2 Proton Reduction

**Figure 9:**
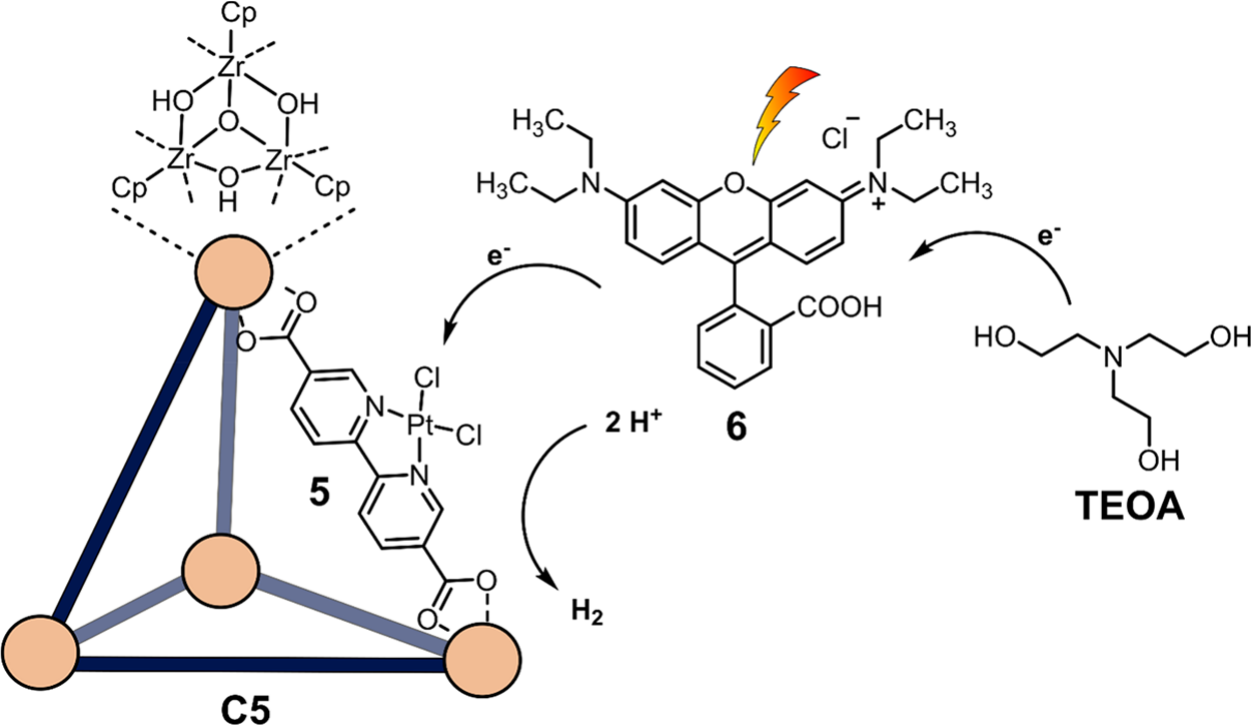
Proton reduction with [(Zr_3_O(OH)_3_Cp_3_)_4_(**5**)_6_)]Cl_4_**C5** as catalyst and rhodamine B (**6**) as photosensitizer. TEOA acts a sacrificial reductant.

Hong and co-workers were the first to report cages bearing zirconocene clusters as metal nodes, which gained popularity because of their high stability and the relatively easy synthesis.^118−121^ Yuan and co-workers synthesized [(Zr_3_O(OH)_3_Cp_3_)_4_(**5**)_6_)]Cl_4_ (**C5**), with proton reduction catalyst **5** incorporated as ligand (Figure 9).^122^ A hydrogen evolution rate of 10.5 mmol g^–1^ h^–1^ over the course of 27 h was obtained using TEOA as the electron donor and rhodamine B (**6**) as photosensitizer. The activity of **C5** was compared to the free ligand **5** as reference. Interestingly, for the first 5 h, similar activity was observed as for the cage. However, the molecular reference ligand **5** then lost its activity, while **C5** continued to produce H_2_ for another 7 cycles of 3 h with minor loss of activity. Overall, the cage analogue is 1.8 times more productive than the reference. The higher stability, and thus overall better productivity of **C5**, is a result of site isolation of the complex. This causes isolation of the metal complex from other metal complexes and prevents degradation to nanoparticles.^123^

**Figure 10:**
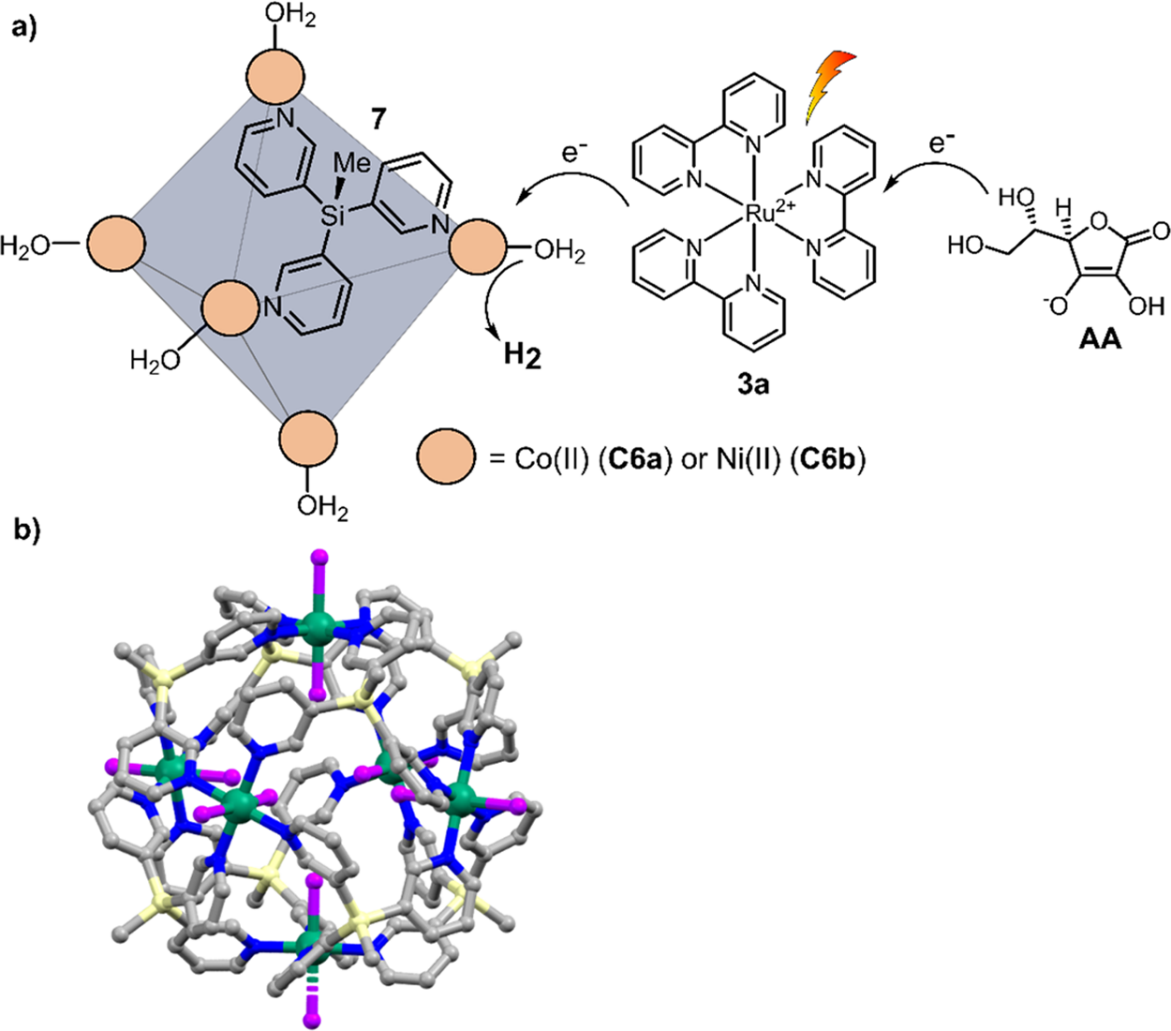
(a) Photochemical proton reduction catalyzed with [M_6_**7**_8_] (M = Co(II) **C6a** or Ni(II) **C6b**) cages, Ru(bpy)_3_Cl_2_**3a** as PS, and AA as sacrificial donor in ascorbate buffer solution (pH = 4). (b) Crystal structure of **C6a**, atoms: C = gray, Co = green, Cl = purple, N = blue, Si = pale yellow.^124^

In 2017, Boomishankar and co-workers used a fully water-soluble octahedral [Co_6_**7**_8_] **C6a** (**7** = tripodal silane ligands MeSi(py)_3_) cage for photochemical proton reduction, in conjunction with Ru(bpy)_3_^2+^**3a** as PS and ascorbic acid (AA) as a sacrificial reductant (Figure 10a).^124^ As shown in the X-ray structure of octahedral cage **C6a**, each Co(II) node is coordinated by one chloride ion pointing inside the cavity (Figure 10b). In addition, each Co(II) center coordinates four pyridine donors and one water molecule pointing outside of the cavity. This structure suggests that the cage cannot bind additional guest molecules in its internal cavity, as it is fully occupied by chloride ligands. Therefore, proton reduction likely occurs outside of the cavity. In photochemical hydrogen evolution, a TON of 43 was obtained after 2 h irradiation of the reaction mixture and a TOF of 21.5 h^–1^ in an aqueous phosphate buffer solution (pH = 7). Control experiments showed almost no hydrogen production in the absence of the PRC. The cease of photocatalytic H_2_ production after 2 h was investigated by adding additional SED, PS, and catalyst one by one, none resuming the H_2_ production. Photocatalytic H_2_ production was only resumed after adding additional PS and catalyst simultaneously, suggesting rapid degradation of photosensitizer **3a** and catalyst **C6a.** A Pourbaix diagram (plotting the potential of redox events vs pH), obtained from differential pulse voltammetry (DPV) in Briton–Robinson buffer, displays a linear dependence of the reduction Co(II)/Co(I) with a slope of −59 mV/pH in the range of pH 2.8–5.6, indicating the mechanism involves a PCET in this pH regime. This is beneficial, as PCET lowers the thermodynamic barrier by avoiding charge accumulation.^125^ Changing the metal node to nickel led to increased activity of the resulting cage [Ni_6_**7**_8_] (**C6b**).^126^ As in the previous system, the authors state that the polypyridyl silane ligands **7** stabilize low oxidation states on the nickel ions and act as redox-active functions to assist the PCET process. In contrast to the Co(II) analogue, **C6b** is significantly more stable and showed to be active for 69 h under irradiation in photocatalytic conditions, with a maximum TOF of 41 h^–1^ and TON of 2824.

**Figure 11:**
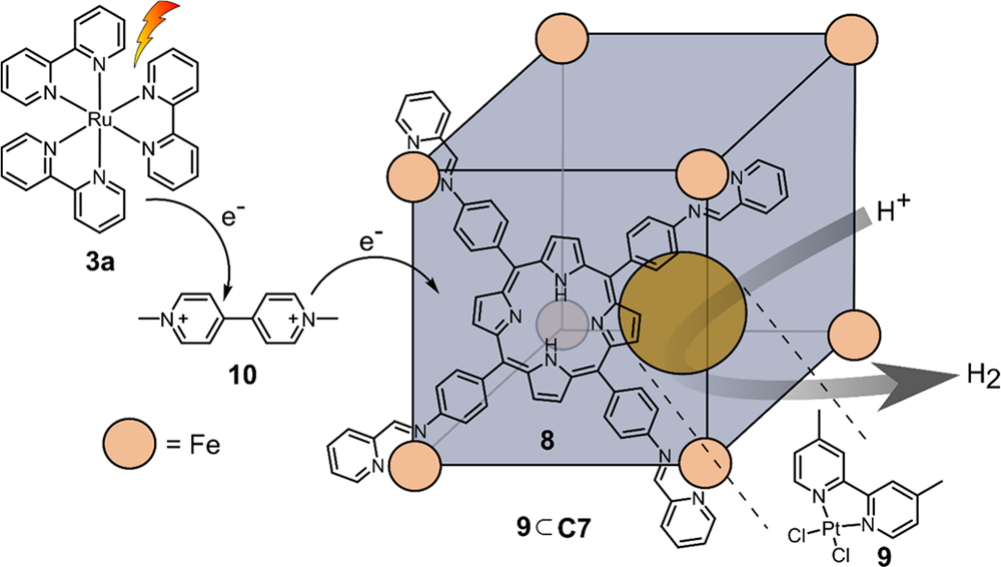
Cubic porphyrin **8** based cage [Fe_8_**8**_6_] **C7** that may encapsulate Pt^II^(dmb)Cl_2_ catalyst **9** for the production of hydrogen with Ru(bpy)_3_Cl_2_**3a** as photosensitizer and methyl viologen **10** as redox mediator.

Nitschke and co-workers have previously reported the binding of neutral guest molecules with large π-conjugated systems into cubic porphyrin cages ([Fe_8_**8**_6_], **C7**) formed by subcomponent self-assembly.^127^ Following the same principle, Sakai and co-workers introduced molecular Pt(II) PRCs such as Pt^II^(dmb)Cl_2_ (**9**) bearing π-conjugated ligands as guest into the cavity of **C7** (Figure 11).^128^ In addition to **3a** as PS and ethylenediaminetetra acetic acid (**EDTA**) as sacrificial reductant, methyl viologen (**10**) was used as electron relay in the photochemical system with [**9⊂C7**] as catalyst in aqueous acetate buffer at pH 5. The role of the redox mediator is to act as shuttle between PS and catalyst. **10** oxidatively quenches the excited PS* quickly and thus reduces unproductive energy transfer from PS*. Reduced **10** then diffuses to the catalyst and transfers one electron, thus leading to unidirectional electron flow. It has been shown previously that electron relays can facilitate both charge separation and efficient electron transfer between PS and catalyst.^129,130^ A TOF = 23 h^–1^ and TON = 58 after 200 min of irradiation is obtained, which is significantly higher than the reference system (complex without cage; TOF = 1 h^–1^ and TON = 6).

#### 3.2.3 CO_2_ Reduction

**Figure 12:**
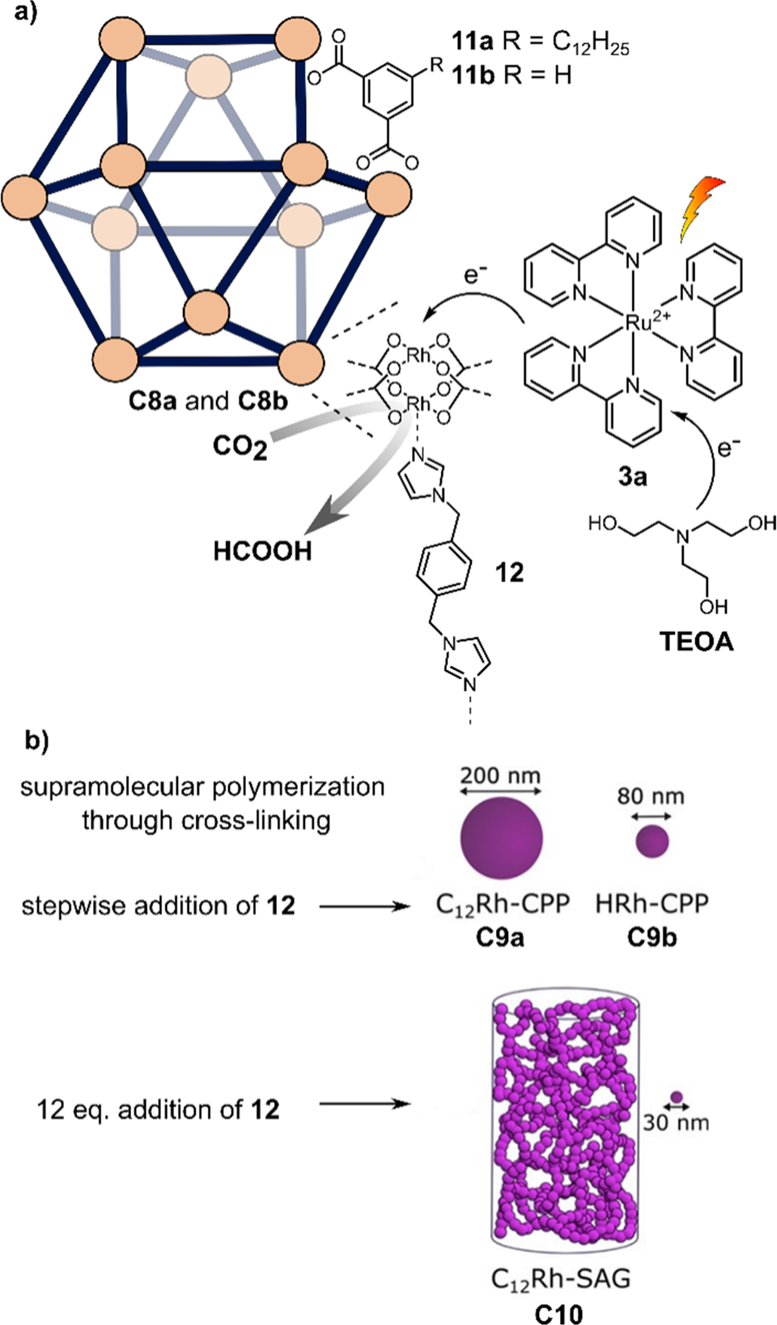
(a) Photocatalytic system for CO_2_ reduction to formate based on supramolecular Rh(II)-paddlewheel (Rh_2_)_12_L_24_ spheres **C8a** and **C8b** with Ru(bpy)_3_Cl_2_**3a** as the photosensitizer and TEOA as the sacrificial electron donor. (b) Polymers are obtained by mixing spheres **C8a** and **C8b** with ditopic ligand **12**.^131^ Adapted with permission from ref (122). Copyright 2022 American Chemical Society.

Wisser and co-workers prepared Rh(II)-paddlewheel cages **C8a** and **C8b** for light-driven CO_2_ reduction with dodecoxybenzene-1,3-dicarboxylic acid (**11a**) and isophthalic acid (**11b**) as ligands, respectively (see Figure 12a).^131^ Uniquely, **C8a** and **C8b** can be functionalized with axial ligands on both the endohedral rhodium and the exohedral rhodium center. Thus, ditopic ligands can be used to cross-link the spherical cages to form supramolecular polymers (Figure 12b). While stepwise addition of ditopic imidazole ligand bix (**12**) led to spherical coordination polymer particles (C_12_ Rh-CPP, **C9a**, and HRh-CPP, **C9b**), direct addition 12 equiv of **12** to **C8a** induced formation of a supramolecular aerogel (C_12_ Rh-SAG, **C10**). In both cases, the axial ligand **12** coordinates to the exohedral site of the Rh(II) dimer nodes, leaving the endohedral Rh(II) open as catalytic center. Photochemical CO_2_ reduction with the different species was performed, using Ru(bpy)_3_ Cl_2_**3a** as PS and TEOA as sacrificial reductant.

Despite the difference in size and porosity, C_12_-containing polymers **C9a** and **C10** featured similar photocatalytic activity, obtaining a TOF of 59 h^–1^ for the conversion of CO_2_ exclusively into HCOOH. In the case of the single sphere **C8a**, a slightly lower activity was observed (TOF = 52 h^–1^). The effect of the axial ligand was tested by addition of monodentate 1-benzylimidazole, which coordinated to the exohedral Rh(II) center. A TOF of 58 h^–1^ was observed, clearly demonstrating the positive influence of axial coordination. As shown previously, the axial ligand on the exohedral rhodium center leads to increased electron density on the endohedral rhodium center, which results in improved CO_2_ reduction.^132^ The same effect is anticipated to lead to better performance of polymers **C9a** and **C10**. Single sphere **C8b** displayed slightly lower activity (TOF = 43 h^–1^), likely due to the lack of alkyl chains which contribute to increasing the electron density. Recycling experiments using **C9a** revealed that the polymeric catalyst features similar initial activity for the first 2 h within 4 cycles. The activity rapidly decreased after 2 h, which was explained by photosensitizer degradation. Apparently, the cages remain stable even after 4 runs of 8 h. Additionally, TEM experiments showed that no Rh nanoparticles were formed during catalysis, confirming the stability of the supramolecular polymeric network and the cages themselves.

### 3.3 Encapsulated Photosensitizer

Supramolecular cages can host guest molecule by employing attractive forces such as electrostatic interactions, or using hydrophobic effects.^24^ These strategies have also been used to encapsulate molecular photosensitizers. In the following, we will discuss recent work on encapsulated photosensitizers. In these examples, the metal nodes of the cages act as catalytic centers.

**Figure 13:**
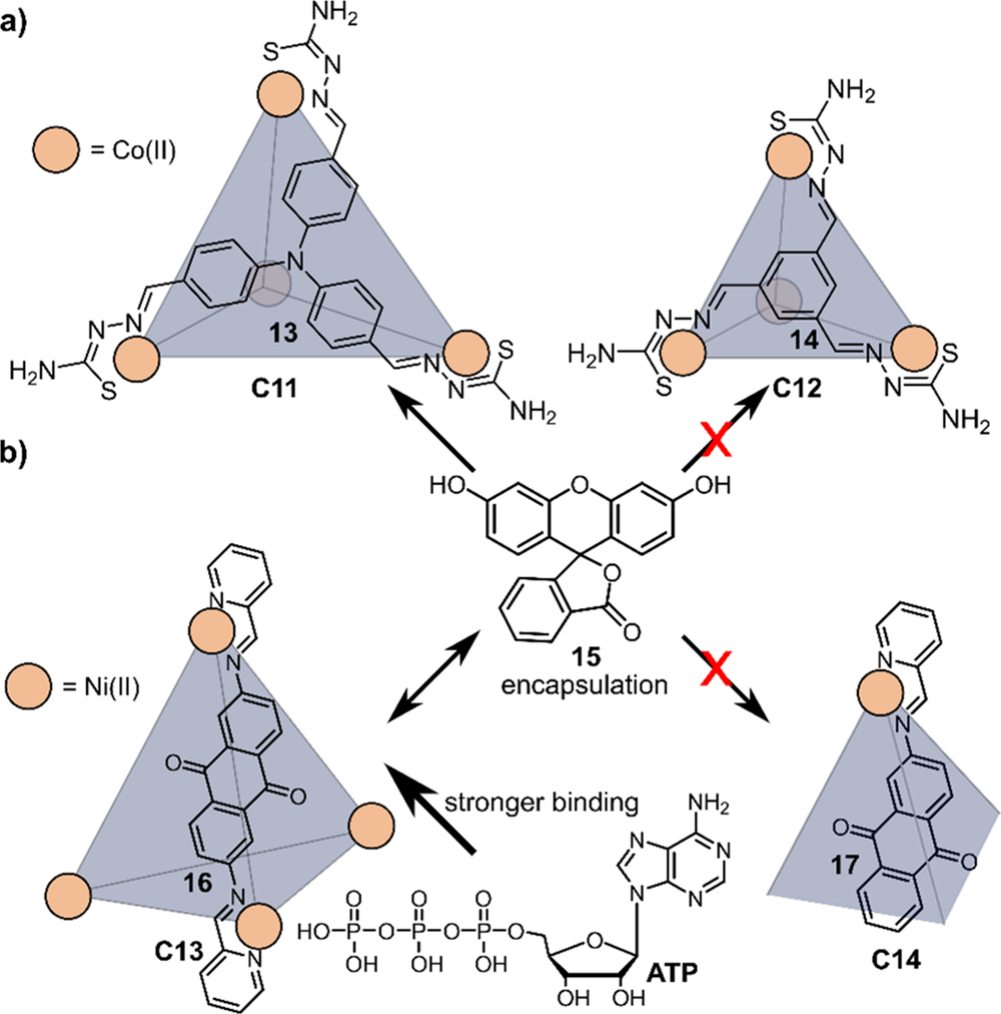
(a) Host–guest binding properties of [Co_4_**13**_4_] (**C11**) and [Co_4_**14**_4_] (**C12**). **C11** readily encapsulates one molecule of fluorescein **15**, while **C12** is too small to bind **15**. (b) [Ni_4_**16**_6_] **C13** binds **15**. Addition of ATP leads to expulsion of **15** due to stronger binding. [Ni**17**_3_] **C14** is too small to bind either of these guests.

Duan and co-workers designed tetrahedron [Co_4_**13**_4_] (**C11**), which can be prepared via self-assembly of triphenylamine ligands substituted with thiosemicarbazone handles that can coordinate to Co^II^ cornerstones as the catalytic centers (see Figure 13a).^133^ Binding studies revealed that one fluorescein molecule **15** can bind in the cavity of **C11**. Optimal conditions for photocatalytic H_2_ evolution were found to be a H_2_O:EtOH (1:1) mixture, using triethylamine (Et_3_N) as sacrificial electron donor. The initial TOF for [**15⊂C11**] was calculated at 750 h^–1^, and the value for the TON was 11000 over a period of 24 h, which was the highest activity for a cobalt–fluorescein system at the time. For comparison, a smaller cage [Co_4_**14**_4_] (**C12**) was synthesized with comparable redox potentials and coordination structure yet with a smaller cavity compared to **C11** and unable to bind **15**. Under similar photocatalytic proton reduction conditions, a decrease in initial TOF (450 h^–1^) and TON (4500) were obtained. Fluorescence quenching studies revealed that both cages follow a different mechanistic pathway. As a result of preorganization, the mechanistic pathway of **C11** is dominated by PET from 15* to **C11** (oxidative quenching), inducing immediate H_2_ evolution. The mechanism in **C12** is initiated by reductive quenching of 15* to **15**^–^, which then has to diffuse to the cobalt center for H_2_ evolution.

He and co-workers synthesized electron-deficient redox cage [Ni_4_**16**_6_] (**C13**, see Figure 13b), containing Ni^II^ as the catalytic center, with similar binding properties of **15** as in **C11**.^134^ Proton reduction was facilitated in a H_2_O:EtOH (1:1) mixture with 12% Et_3_N as the sacrificial electron donor. The calculated TON obtained was up to 1200 mol of hydrogen per mole of catalyst under optimal conditions. Using smaller bowl-shaped [Ni**17**_3_] (**C14**) that cannot bind **15** in its cavity resulted in lower H_2_ evolution up to 0.5 mL over a period of 24 h (vs >1.2 mL for **C13**).

In a competition experiment, adenosine triphosphate (ATP) was used to block the cavity as it binds more strongly than **15**. Upon addition of ATP to the host–guest complex [**15⊂C13**], all bound **15** is substituted by ATP. Expulsion of **15** from the cage led to a 65% decrease in H_2_ evolution activity compared to the original system. Addition of ATP to **C14** only lowered the activity slightly, not having as much effect on the electron transfer as compared to the inhibition in **C13**. Similar differences in mechanistic pathways are assumed as for **C11** and **C12**.

**Figure 14:**
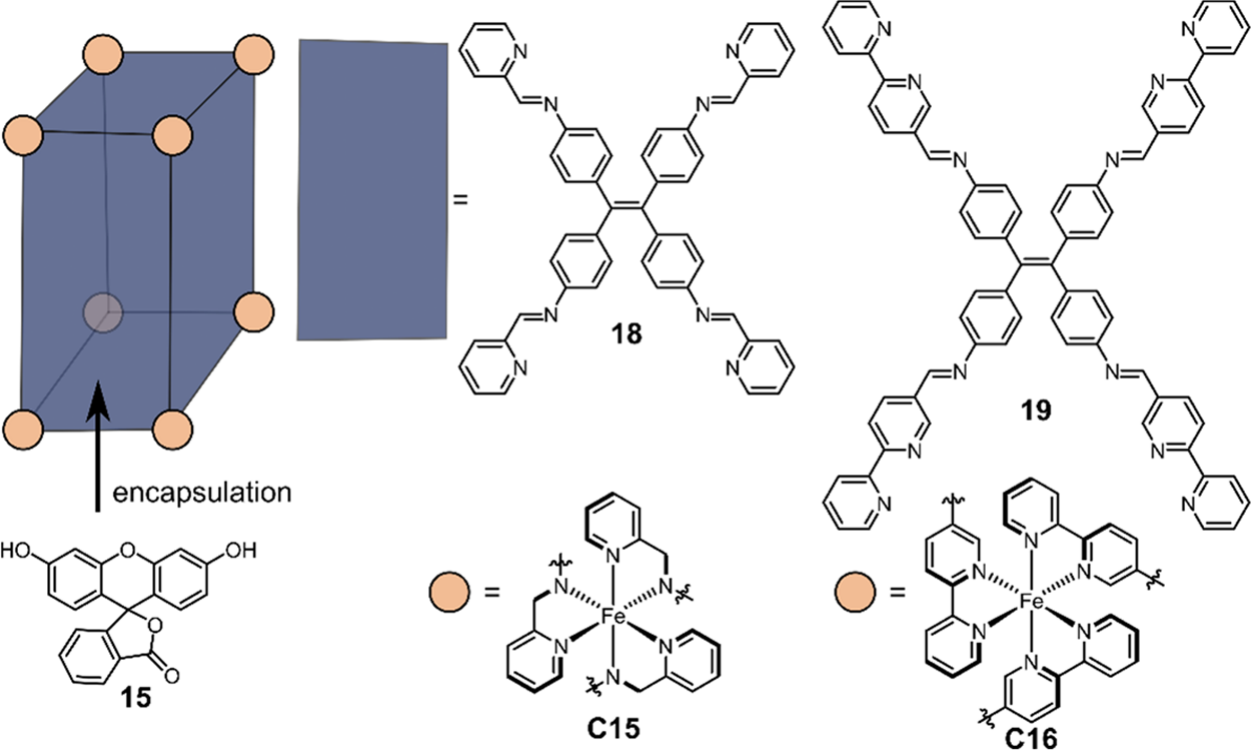
Schematic structure of box-like cages **C15** and **C16** based on tetraphenylethylene ligands **18** and **19** that is able to encapsulate fluorescein **15** and use the Fe centers for H_2_ production.

Duan and co-workers continued to synthesize box-like cages [Fe_8_**18**_6_] (**C15**) and [Fe_8_**19**_6_] (**C16**), bearing Fe^II^(bpy)_3_ and Fe^II^(2-pyCH_2_=N)_3_ cornerstones and tetraphenylethylene ligands **18** and **19** as backbones, respectively (Figure 14).^135^ The cavity of **C16** is large enough to bind two fluorescein molecules **15**, leading to stronger quenching of the fluorescence compared to **C15**, which can bind only one molecule of **15**. The binding constant for two **15** in the cavity of **C16** is larger than for only one (positive cooperativity). As a result of this binding, **C16** produces more H_2_ under optimal conditions (MeCN: H_2_O, pH = 11). Similar to **C11**, fluorescence quenching and lifetime studies indicate a PET from 15* to both **C15** and **C16**.

**Figure 15:**
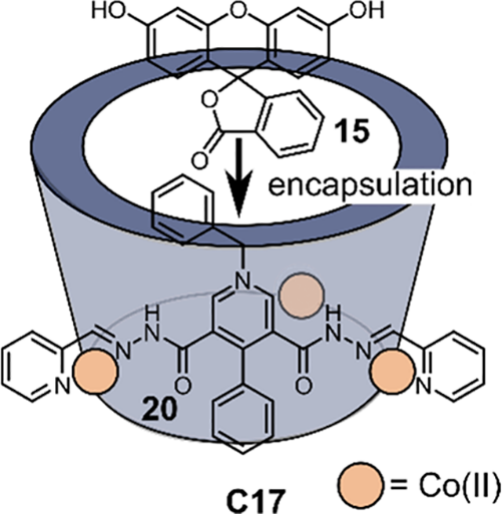
Schematic structure of DHPA moieties containing ligand **20** for the self-assembly of [Co_3_**20**_3_] bowl **C17** for fluorescein **15** encapsulation.

Dihydropyridine amido moieties (DHPA) are important in nature, as they play a role in electron transfer, and it is the key molecular structure in nicotinamide adenine dinucleotide (NADH) (Figure 15).^136,137^ Duan and co-workers incorporated DHPA groups in the ligand backbone of **20** to coordinate to Co^II^ ions, which leads to formation of supramolecular barrel [Co_3_**23**_3_] (**C17**).^138^ Host–guest binding studies revealed binding of **15** in the cavity of the supramolecular barrel, facilitating preorganization of the photosensitizer and the catalytic centers. Photocatalytic H_2_ evolution was observed in a MeCN:H_2_O mixture while using 5% Et_3_N as a sacrificial electron donor, obtaining up to a TON value of ca. 400 mol H_2_ per mole catalyst and a TOF value of ca. 100 mol H_2_ per mole per catalyst per hour. To confirm if the photocatalytic H_2_ evolution occurs in the cavity, ATP was added because studies revealed it binds stronger in the cavity of **C17** compared to **15**. Addition of ATP to the catalytic system stopped the H_2_ production completely, inhibiting the photocatalytic reaction. This confirms that photoinduced H_2_ production occurs in the cavity rather than via a diffusion dependent homogeneous system.

**Figure 16:**
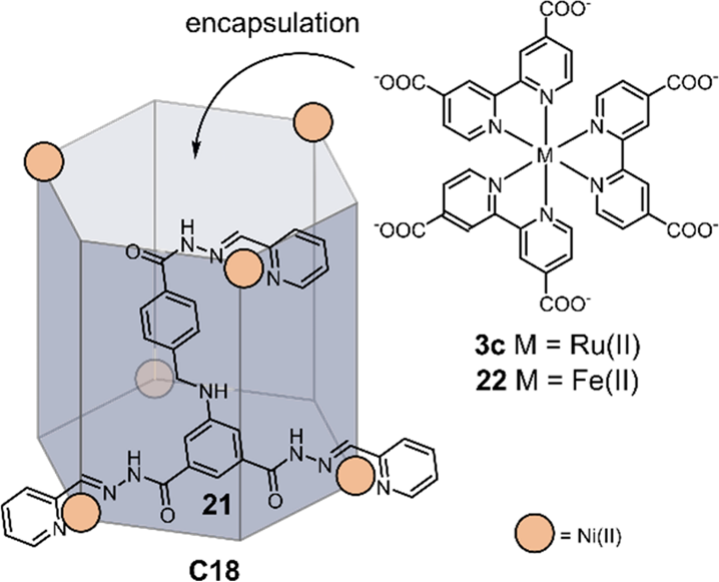
Schematic structure of the [Ni_6_**21**_6_]^12+^**C18** cage, which can bind anionic Ru(dcbpy)_3_^4–^**3c** through electrostatic interactions and uses the NiII nodes as catalysts for hydrogen production.

He, Guo, and co-workers synthesized [Ni_6_**21**_6_]^12+^ (**C18**), in which the ligand **21** contains three binding sites (Figure 16).^139^ Because **C18** is cationic, photosensitizer Ru(dcbpy)_3_^4–^**3c** present in its anionic form in alkaline media binds through electrostatic interactions in the pocket of **C18**. Encapsulation of the photosensitizer was confirmed by MS and ^1^H NMR. Under optimal conditions, an initial TOF of 1100 mol hydrogen per mole of catalyst per hour and TON of 1600 mol hydrogen per mole catalyst was achieved, using TEOA (15%) as a sacrificial reductant in a H_2_O:EtOH (1:1) mixture (0.26 mL H_2_ in 5 h). As expected from previous systems, an oxidative quenching pathway was established by transient absorption spectroscopy (TA). A peak was observed at λ = 420 nm, in line with the formation of Ru^III^ complexes expected to form by oxidative quenching. No signals of Ru^I^ complexes were observed, which would form by reductive quenching. When an equimolar amount of Fe(dcbpy)_3_**22** was added to the solution, only 12.2% H_2_ evolution was observed compared to the original system, indicating inhibition of the cavity by the iron complex. However, addition of **22** did not lead to luminescence quenching of the **3c**, excluding the possibility of competitive electron transfer between the complexes and having exclusively competitive binding in the cavity.

**Figure 17:**
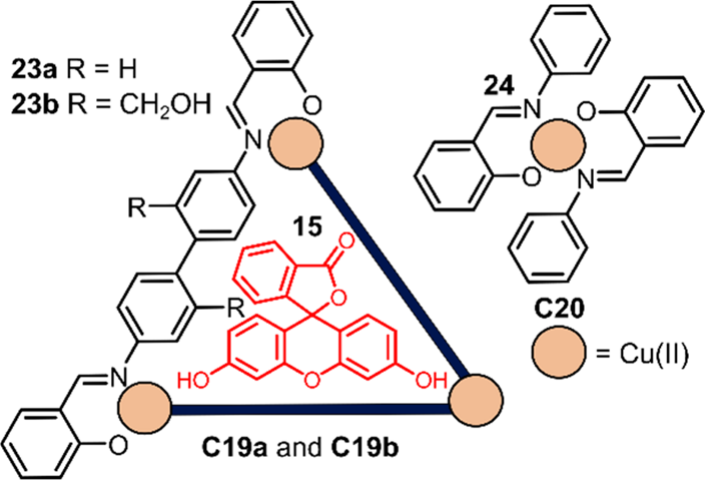
Schematic structure of the supramolecular triangles **C19a** and **C19b**, based on ligands **23a** and **23b**, respectively, that can encapsulate fluorescein **15**. Mononuclear reference complex **C20** based on ligand **24** is also shown which cannot encapsulate **15**.

Duan and co-workers furthermore investigated photocatalytic H_2_ evolution with redox-active host–guest complex [**15⊂**Cu^II^_3_**23a**_3_] ([**15⊂C19a**, see Figure 17).^140^ About 50% of the (excited) 15* are oxidatively quenched when bound in **C19a**. Cyclic voltammetry of the **C19a** revealed a reduction potential for Cu^I^/Cu^0^ of −0.75 V vs Ag/AgCl (at 1.0 mM **C19a**), indicating that proton reduction is feasible. Optimal results in photochemical H_2_ production were obtained with TEA as sacrificial electron donor in a H_2_O:EtOH mixture at pH = 12.5, obtaining up to TON of approximately 1200 mol of hydrogen per mole catalyst over a period of 20 h. Inhibition with ATP resulted in 80% less H_2_ production under optimal conditions, displaying the importance of binding **15** in the pocket of the **C19** to maximize H_2_ production. In comparison, mononuclear complex [Cu^II^**24**_2_] (**C20**), featured only a TON of 100 mol of hydrogen per mole catalyst under the same conditions. A more water-soluble analogue [Cu^II^_3_**23b**_3_] (**C19b**, see Figure 17) was synthesized, bearing two hydroxymethyl groups per ligand. Interestingly, the photocatalytic H_2_ evolution activity of **C19b** was very similar under the same conditions for **C19a** and did not decrease significantly in a 1:4 EtOH:H_2_O mixture, providing strategies for the development of highly efficient water-soluble homogeneous proton reduction catalysts.

**Figure 18:**
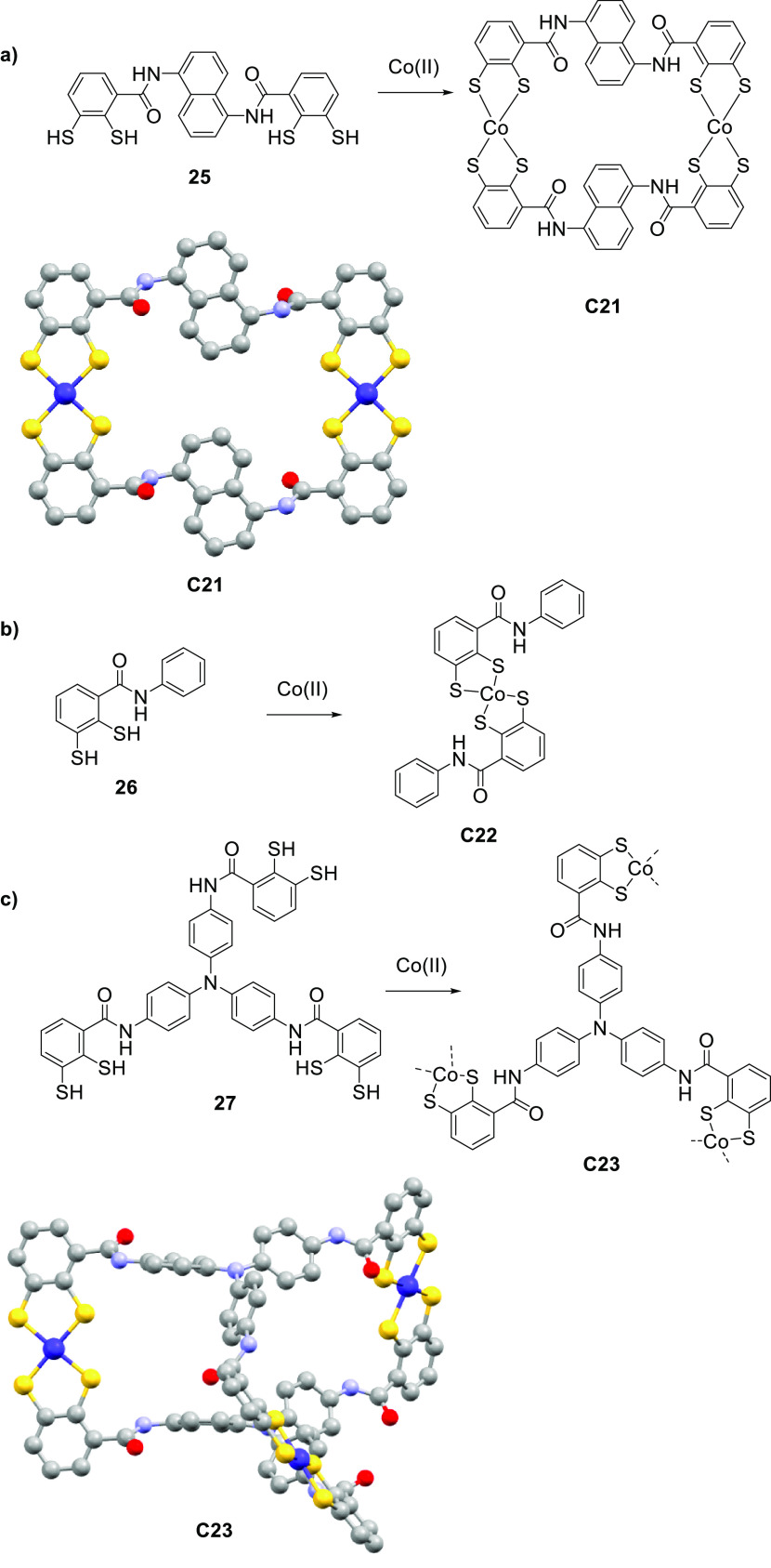
(a) Structure and crystal structure of **C21** and ligand **25**. (b) Structure of **C22** and ligand **26**. (c) Structure and crystal structure of **C23** and **27**. Atoms: C = gray, Co = purple, O = red, N = lilac, S = bright yellow.^141^

Zhao, Duan and co-workers prepared a negatively charged supramolecular square [Co_2_**25**_2_]^4–^ (**C21**) and studied supramolecular complex formation with Ru(bpy)_3_^2+^**3a**.^141^ The crystal structure of the host–guest complex indicated binding of two **3a** molecules to square **C21** (Figure 18a), which is stabilized by electrostatic interactions and hydrogen bonding while substituting the counterion. The host–guest binding of **C21** and two **3a** complexes was also confirmed by isothermal titration calorimetry (ITC). Based on fluorescence quenching experiments, the photocatalytic pathway was found to be similar as previous host–guest systems. The excited state of the photosensitizer is directly oxidatively quenched by the Co^II^ cornerstones. In this system, optimal conditions were obtained with AA as sacrificial electron donor, with up to 400 μL of H_2_ in 9 h with a corresponding initial TOF of 40 h^–1^. H_2_ evolution shows a linear relation with the concentration of **C21**, which is similar to previous systems.^135^ A mononuclear variant [Co**26**_2_]^2–^ (**C22**) was synthesized (Figure 18b), which under the same conditions only yielded trace amounts of H_2_. In addition, a triangular prismatic cage [Co_3_**27**_2_]^6–^ (**C23**) was synthesized (Figure 18c). ITC assays confirmed the host–guest formation of [**3a**_3_**⊂C23**], replacing all Et_4_N^+^ counterions. H_2_ evolution with **C23** was investigated, which under similar conditions as for **C21** yielded 350 μL of H_2_, which is slightly lower than **C21**.

#### 3.3.1 H_2_S Splitting

**Figure 19:**
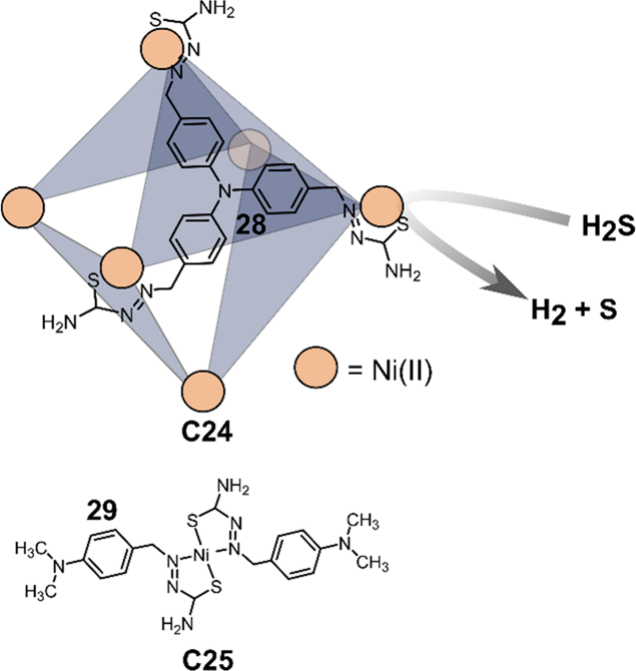
Schematic structure of triarylamine based ligand **28** in [Ni_6_**28**_4_] cage **C24** and mononuclear analogue **C25** based on ligand **29**, used for photochemical H_2_S splitting.

Reek and Duan prepared an octahedron Ni^II^-cage [Ni_6_**28**_4_] (**C24**) (Figure 19) for photochemical splitting of H_2_S into H_2_ and elemental sulfur.^142^**C24** bears tritopic triphenylamine ligands **28** with hydrazinecarbothioamide coordinating moieties, which are self-assembled *in situ* during cage formation. The photosensitizer fluorescein **15** can bind in the cavity, once more preassembling both PS and the catalytic center.^143^ Photochemical H_2_ evolution was performed using TEA as a sacrificial electron donor in a H_2_O:EtOH (1:1) mixture at pH = 12.6, obtaining an initial TOF of 1250 mol of hydrogen per mole of catalyst per hour and a TON of 25000 per mole of **C24**. As the 15* follows an oxidative quenching pathway because of the close proximity to the catalyst, the oxidized dye **15**^**+**^ can oxidize S^2–^ to elemental sulfur, and indeed formation of a yellow powder is observed during the reaction. Mononuclear complex [Ni**29**_2_] (**C25**) displays significantly lower yields in photochemical H_2_ evolution, and no elemental sulfur was formed, indicating that the S^2–^ was not oxidized under these conditions. In another experiment where glucosamine was added as competitive binder for the cavity in **C24**, also no sulfur was produced, demonstrating the importance of preorganization of PS and the catalyst.

**Figure 20:**
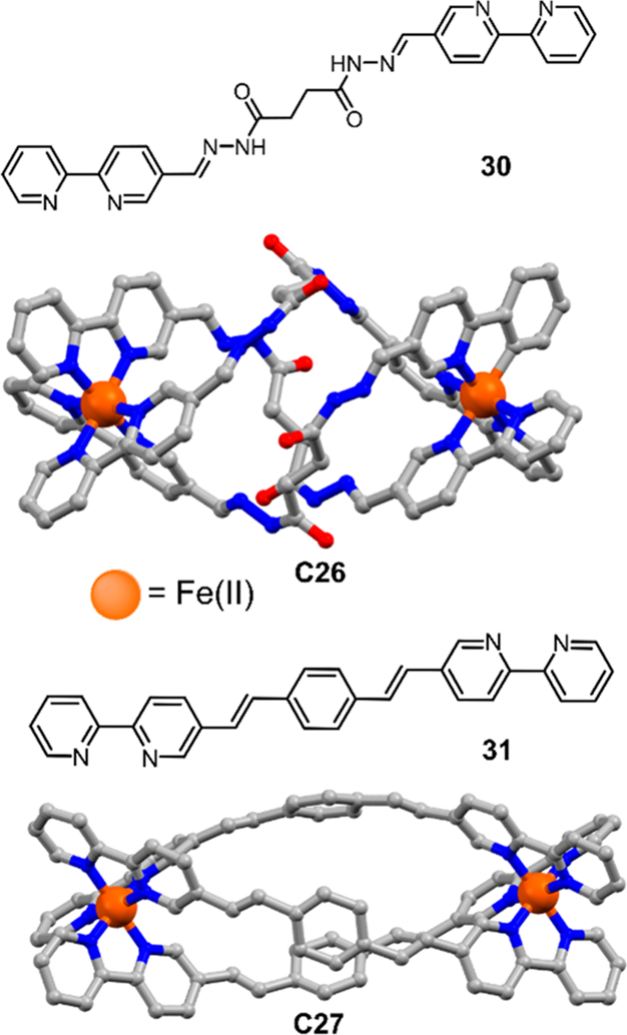
Crystal structures of [Fe_2_L_3_] **C26** and **C27**, and chemical structures of their linkers **30** and **31**. Atoms: C = gray, Fe = orange, O = red, *N* = blue.^144^

Recently, Jing, Duan, and co-workers synthesized helical capsule [Fe_2_**30**_3_] (**C26**) for simultaneous H_2_S splitting and hydrogenation of nitrobenzene (Figure 20).^144^ Both capsules contain Fe(bpy)_3_^2+^ units as metal nodes, representing the active catalyst. In the cavities, one molecule of fluorescein (**15**) can be encapsulated as PS. The reaction follows an oxidative quenching pathway. Photochemical H_2_ evolution was observed in a MeCN:H_2_O (1:1) solution at pH = 11.5. A TON of 276.7 and a TOF up to 19.7 h^–1^ were observed. A yellow powder was also obtained from the reaction, which could be characterized as elemental sulfur. Nitrobenzene is able to bind in the cavity of **C26**, resulting in hydrogenation to aniline in 99% yield under photocatalytic conditions. Larger substrates were also explored, e.g., 1-nitronaphthalene, 9-nitroanthracene, and 1-nitropyrene, requiring longer reaction time to reach 99% yield as the size of the substrate increases (up to 420 min for 1-nitropyrene). The longer reaction time is also in line with kinetic studies that observe an initial rate of 1.87 mM min^–1^ for nitrobenzene, compared to 0.28 mM min^–1^ for 1-nitropyrene. A larger substrate, 2,4,6-triphenylnitrobenzene, that is larger than the windows of the cavity of **C26**, could not be hydrogenated to the aniline product and only a small amount of hydrogen gas was observed.

The authors also further investigated the influence of the hydrazide groups as hydrogen bond donors for preorganization of the nitrobenzene substrates. Another capsule bearing a *p*-divinylbenzene unit [Fe_2_**31**_3_] (**C27**) was synthesized containing no hydrogen bonding motifs, yet a slightly larger opening (4.9 Å vs 3.8 Å for **C27**) and similar electrochemical properties. Interestingly, higher photocatalytic H_2_ production was obtained under the same optimal conditions as for **C26**, having the TON reach up to 416.7 and TOF of up to 41.7 h^–1^. As expected the reactivity in the hydrogenation reaction using **C27** was lower, needing a reaction time of 90 min to reach 99% for nitrobenzene and up to 560 min for 1-nitropyrene (compared to 420 min for **C26**), regardless of the larger opening size of the portal to the cavity. These results show that the larger window of **C27** is more favorable for photocatalytic H_2_ evolution, likely because of the easier complexation with **15**, whereas the hydrogenation of nitrobenzene is enhanced by hydrogen bonding using **C26** as a cage.

#### 3.3.2 Proton and CO_2_ Reduction

**Figure 21:**
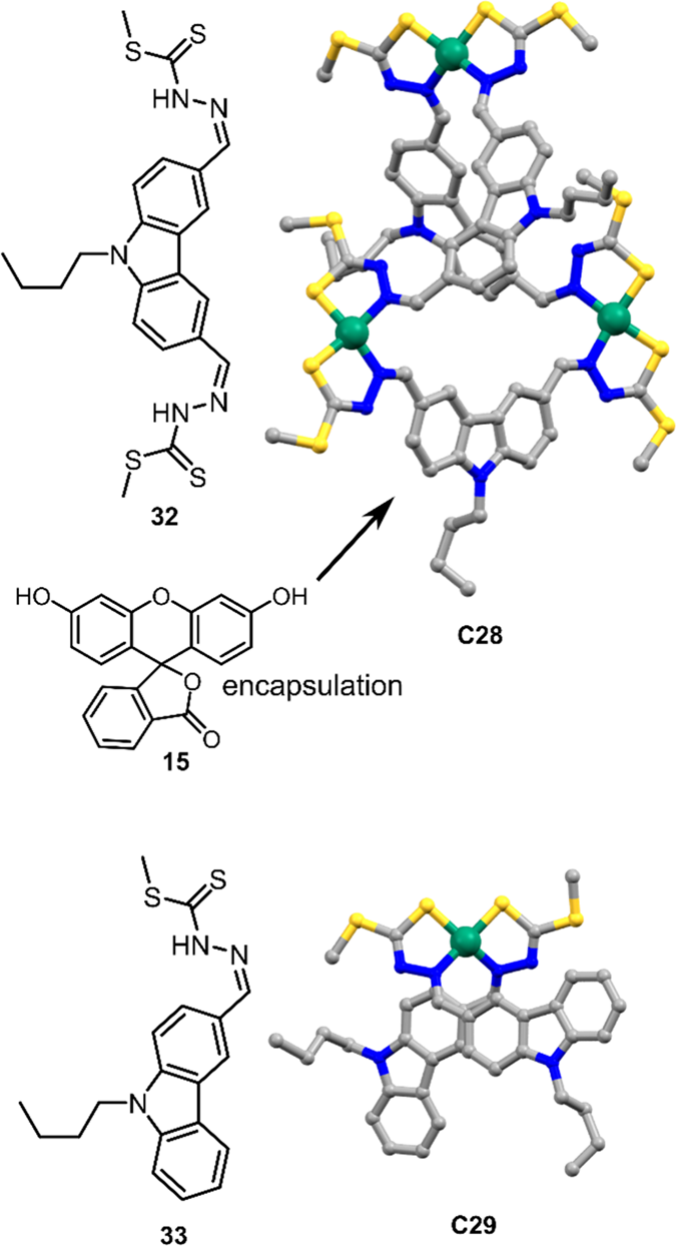
Crystal structures of **C28** and mononuclear complex **C29**, along with the chemical structures of their ligands **32** and **33**, respectively. **C28** is able to encapsulate **15** and perform photocatalyzed proton and CO_2_ reduction. Atoms: C = gray, Ni = green, N = blue, S = bright yellow.^145^

He and co-workers prepared a supramolecular triangle [Ni_3_**32**_3_] (**C28**), where the catalytic active site can be used for proton reduction and CO_2_ reduction to HCOO^–^ (Figure 21).^145^ Binding of PS **15** in the cavity of **C28** enables direct PET from 15* to **C28**, similarly to the systems discussed previously in this section. Optimal conditions were found using TEA as a sacrificial reductant in 1:1 MeCN:H_2_O solvent mixture at pH = 11. The initial TOF for H_2_ formation showed to be 160 h^–1^, with a total TON at 1250 per mole of catalyst over a period of 12 h. Under similar conditions in CO_2_ saturated solvent, HCOO^–^ production was observed in amounts increasing to 0.46 μmol (initial TOF = 0.8 mol of HCOO^–^ per mole of the catalyst per hour, TON = 9.3 per mole of catalyst over 12 h). Only trace amounts of H_2_ were formed during the reaction and no other byproducts, indicating the dominating pathway of CO_2_ reduction over H_2_ evolution.

Interestingly, the mononuclear complex [Ni**33**_2_] (**C29**) did undergo photocatalytic H_2_ evolution (0.93 mL over a period of 12 h) under similar conditions as **C28**, yet no HCOO^–^ was observed in the presence of CO_2_. The photocatalytic mechanism is anticipated to be the same as for **C28**, as the **15** molecules can bind via π–π stacking to the ligands of **C29** (as indicated by ^1^H NMR and NOESY experiments). With a difference in dihedral angle (23.8° for **C28** and 17.0° for **C29**), the authors concluded that the strained coordination in **C28** results in a more distorted coordination at the Ni centers which enables the activation of CO_2_.

### 3.4 Photoactive Cages with Encapsulated or Incorporated Catalysts

In this section, we discuss cages containing photoactive ligands with encapsulated catalysts.

#### 3.4.1 Encapsulated Catalysts

**Figure 22:**
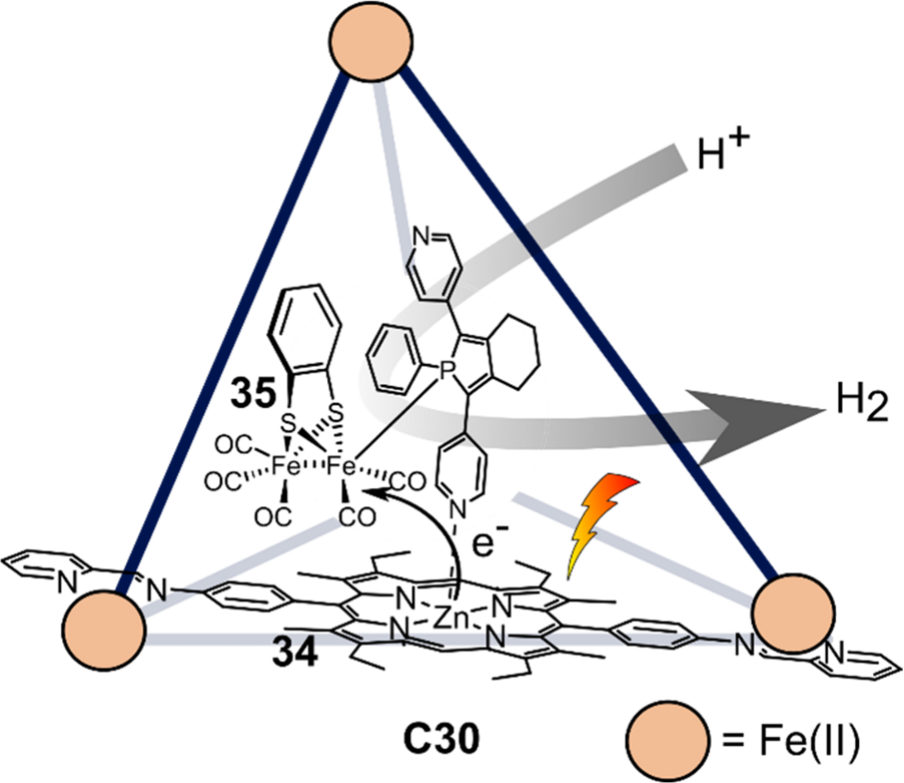
Schematic representation of [FeFe]-hydrogenase mimic **35** encapsulated into porphyrin **34**-based [Fe_4_**34**_6_] cage **C30**, which performs photochemical proton reduction.

Reek and co-workers utilized Nitschke-type cage [Fe_4_**34**_6_] (**C30**) with Zn(II) porphyrin ligands as host for a [FeFe]-hydrogenase mimic **35** containing pyridyl–phosphole ligands (Figure 22).^146^ The pyridyl units coordinate to the Zn(II) of the porphyrins and thereby enable catalyst binding inside of the cage. According to fluorescence quenching titration experiments, one catalyst molecule binds strongly to the cage. In the presence of TFA as a proton source and 4-mercaptobenzoic acid as a sacrificial electron donor, [**35⊂C30**] produced hydrogen under irradiation with an overall TON of 0.4. The system is limited to low acid concentrations in order to prevent pyridine protonation, which leads to catalyst dissociation. Importantly, encapsulation lowered the overpotential required for catalysis as indicated by electrochemical measurements. In addition, time-resolved spectroscopy showed fast PET from the host to the encapsulated catalyst at 0.5 ps, whereas charge recombination occurs in around 37 ps. Single-electron reduction of **35** was confirmed by time-resolved IR spectroscopy, with an overall quantum yield of 1%.

**Figure 23:**
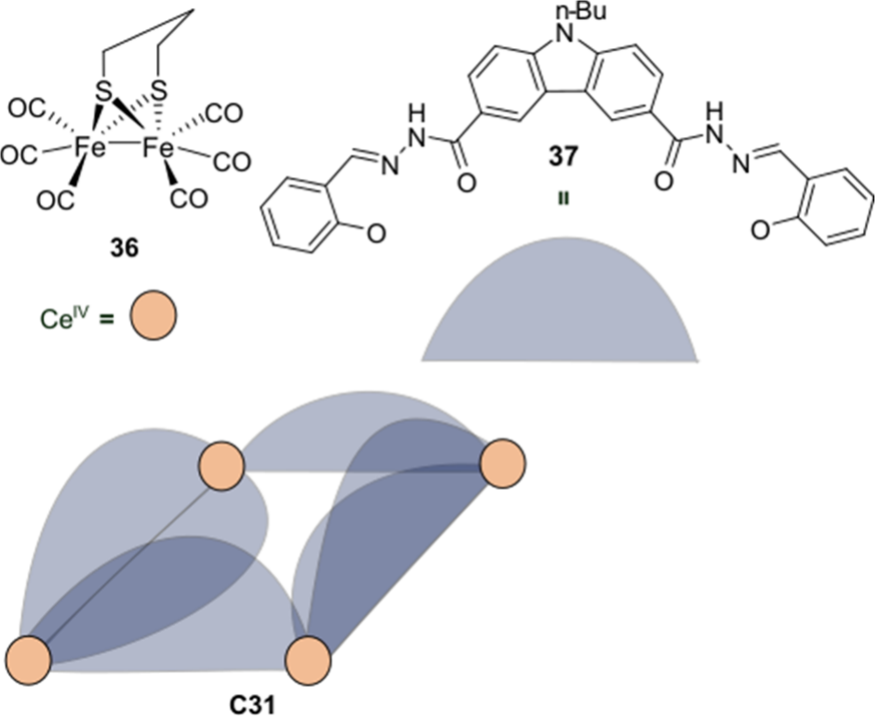
Schematic structure of cage **C31** based on carbazole-containing ligand **37** and nonacoordinate Ce^IV^ atoms that can bind FeFe-hydrogenase mimic **36** for proton reduction.

A second example using a [FeFe]-hydrogenase mimic **36** as catalyst was demonstrated by Duan and co-workers. Cage [Ce_4_**37**_6_] (**C31**) was synthesized using a carbazole photosensitizer in the ligand structure (Figure 23).^147^ Host–guest studies revealed that the proton reduction catalyst **36** binds to the cavity of **C31** in a 2:1 ratio. As seen previously in other host–guest systems, photoluminescence studies showed direct quenching of the photosensitizer, indicating PET from PS to **36**, facilitated by preorganization. Light-driven H_2_ evolution was carried out in a MeCN:DMF:H_2_O (8:1:1) mixture using N^i^Pr_2_ EtH•OAc as a sacrificial electron donor, obtaining a TON of 30 in 4 h with a TOF of 11 h^–1^ in the first hour. The free ligand **37** was used as reference, leading to trace amount of H_2_ production. Additionally, occupation of the cavity by ATP led to complete deactivation of photocatalytic H_2_ evolution, clearly demonstrating the necessity of preorganization. The authors did not mention if the Ce^IV^ ions could play a role in the photocatalytic H_2_ production and did not investigate their redox potential.

#### 3.4.2 Metal Nodes As Catalysts

Finally, it is possible to incorporate both light-harvesting and catalytic centers as building blocks in one cage. This strategy allows to preorganize both functions and at the same time leave the cavity available for substrate or cofactor binding.

##### 3.4.2.1 Proton Reduction

**Figure 24:**
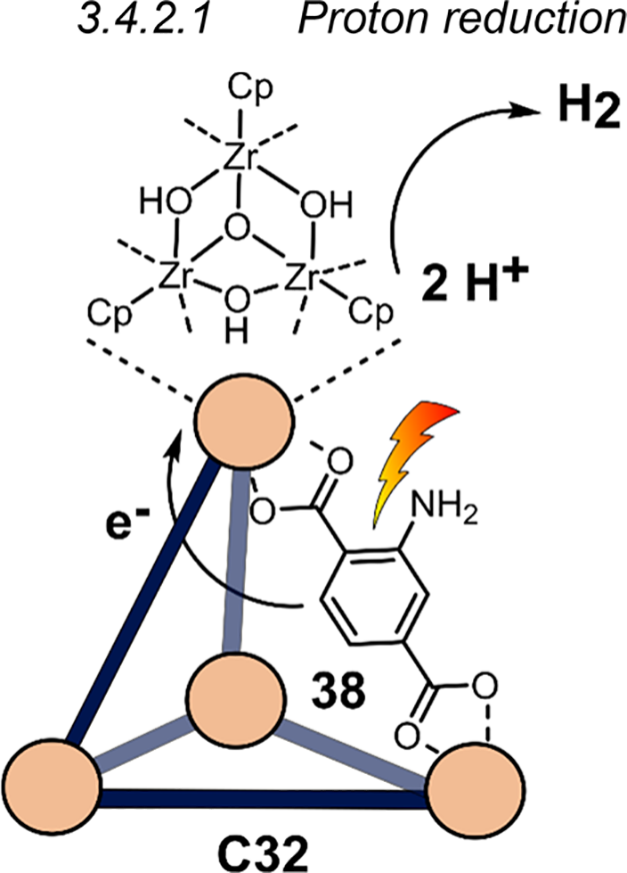
Schematic structure of tetrahedral cage **C32**, based on ligand **38**, used for photochemical proton reduction. The amino groups can be functionalized with Pt nanoparticles to further enhance hydrogen evolution.

Similarly to **C5** (Figure 9), an aniline derivative [(Zr_3_O(OH)_3_Cp_3_)_4_(**38**)_6_)]Cl_4_ (**C32**) was synthesized by Su and co-workers, exhibiting photocatalytic proton reduction using H_2_O as proton source (Figure 24).^148^ Analogous photoactive metal–organic framework (MOF) UiO-66-NH_2_ was used as a reference to compare the activity of **C32** to the corresponding MOF. Interestingly, H_2_ evolution using **C32** (510 μmol g^–1^ h^–1^) is more than 20 times higher than using that of UiO-66-NH_2_ (25 μmol g^–1^ h^–1^) in a 1:3 MeCN:H_2_O mixture. Photoluminescence studies revealed that the emission intensity in **C32** is significantly lower than in UiO-66-NH_2_, proposing more efficient charge transfer from the excited phenylamino group to the Zr-cluster in **C32**. H_2_ evolution could be improved further by supporting Pt NPs on the surface of **C32** as cocatalyst (Pt**/C32**), increasing the H_2_ evolution to an optimal 1058 μmol g^–1^ h^–1^ at 17 wt % Pt NP. Supporting Pt NPs on UiO-66-NH_2_ increased the H_2_ evolution to 62 μmol g^–1^ h^–1^, which is still ca. 17 times lower than the cage.

In a follow-up study, Su and co-workers investigated the effect of how Pt NPs are immobilized on the Zr-cage.^149^ In the previous study, it was reported that the NPs are immobilized on the surface after formation of the cage, while in this study the focus is on in situ immobilization during the formation of the cage (**Pt⊂C32**). H_2_ evolution appeared to be much higher for the in situ immobilized cage, having an H_2_ evolution rate of 10.8 × 10^3^ μmol g^–1^ h^–1^, compared to 1058 μmol g^–1^ h^–1^ of Pt/**C32**. The higher activity is ascribed to the shorter charge transfer distance to the internal Pt NPs in **Pt⊂C32** as they are more likely in the cavity, rather than the NPs being immobilized on the face of the cage in Pt/**C32**.

**Figure 25:**
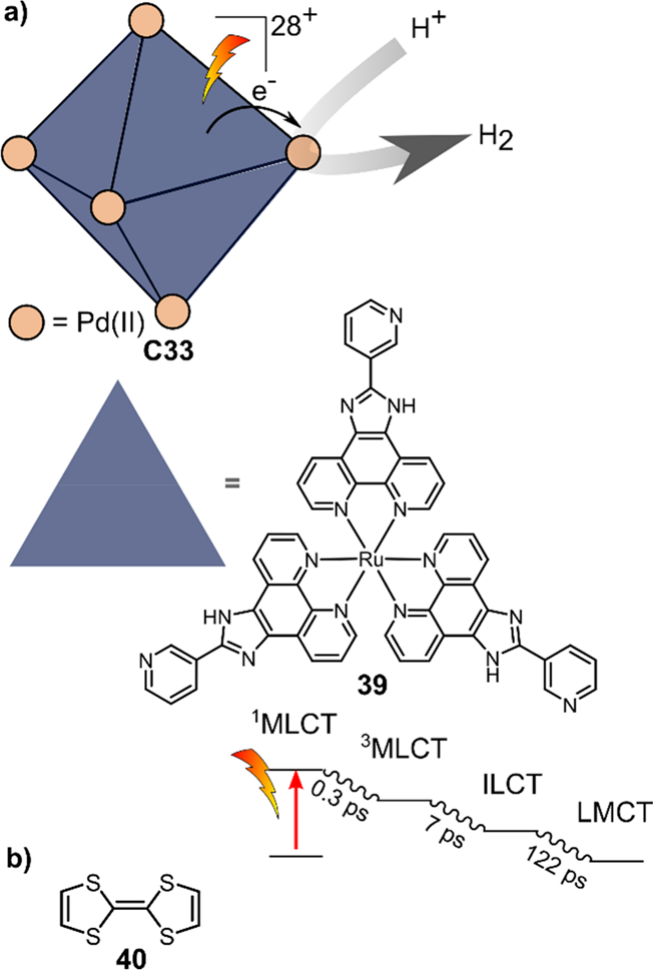
(a) Schematic structure of Pd_6_(RuL_3_)_8_**C33**, which catalyzes light-driven proton reduction and the electron transfer pathway to the catalytically active Pd node as indicated by ultrafast TA spectroscopy. (b) Structure of tetrathiafulvalene **40**, which can be encapsulated in the cage and acts as electron relay.

Su and co-workers synthesized the bimetallic molecular cage [Pd_6_**39**_8_]^28+^ (**C33**, see Figure 25a), with the ruthenium photosensitizer being used as ligand in the cage.^150^ Here the Pd(II) nodes of the cage serve as the active catalyst for proton reduction. Photocatalytic experiments were performed in a 9:1 DMSO:H_2_O mixture using TEOA as the sacrificial electron donor. In the first 3 h cycle, the H_2_ evolution rate of 380 μmol h^–1^ was obtained, declining gradually to ca. 150 μmol h^–1^ in the 16th 3 h cycle. The TOF decreases from 30 h^–1^ in the first cycle to 11 h^–1^ in the 16th cycle. The activity of **C33** in photo-driven proton reduction was seemingly higher by a factor of ca. 2.7 compared to previously reported Ru–Pd assemblies, indicating higher stability in the cage **C33** during proton reduction. This is confirmed by prolonged irradiation of the **C33**, where nanoparticles formed after ca. 100 h of irradiation while using other Ru–Pd assemblies, typically NPs were observed within 48 h of irradiation.

The authors used ultrafast TA to follow the rate of intramolecular electron transfer from the photosensitizer to the catalytic center in DMSO. The reaction is initiated by excitation of the Ru complex to the singlet metal-to-ligand charge transfer (^1^MLCT) state, which within 0.3 ps undergoes intersystem crossing to the ^3^MLCT state (Figure 25a). From here, two pathways are possible, either decay to the ground state, which results in phosphorescence, or intraligand charge transfer (ILCT) from the phenanthroline to the benzimidazole moiety, which occurs in 7 ps. Finally, the electron is transferred to the catalytic site by ligand-to-metal charge transfer (LMCT), which was observed to happen in 122 ps. This shows that electron transfer through covalently bound and coordinatively bound groups occurs fast enough to outcompete BET in the supramolecular sphere.

The same group also investigated the influence of adding electron mediator tetrathiafulvalene (**40**),^151^ which was encapsulated in the cavity of **C33** as a result of the hydrophobic effects (Figure 25b).^152,153^ A significant difference in photocatalytic activity was observed, generating up to 2680 μmol H_2_ with a corresponding TON of 1015 in 47 h by addition of 20 equiv of **40**, compared to 1597 μmol H_2_ (TON = 605) in 47 h when no **40** was added. Lowering the concentration of **40** to 10 equiv displays similar activity for the first 14 h, after which the activity readily declines, indicating that a sufficient amount of **40** not only enhances H_2_ formation but also improves the stability of the cage. Increasing the concentration of **40** to 40 equiv, however, decreases the initial activity in photocatalytic H_2_ formation due to competing or disturbing electron transfer relay from nonencapsulated **40** guests out of the cavity of the cage. The effect of preorganization was then investigated by using Pd(py)_4_^2+^ as proton reduction catalyst and Ru(bpy)_3_^2+^**3a** as PS in addition with **40**, which only obtained half of the activity that **C33** featured for the first 6 h, after which the activity depleted.

##### 3.4.2.2 CO_2_ Reduction

**Figure 26:**
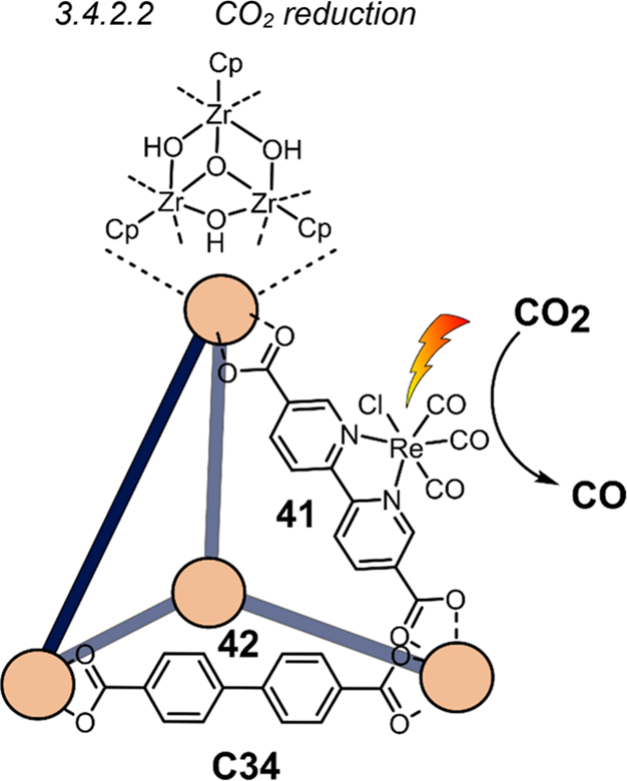
Schematic representation of photochemical CO_2_ reduction by catalyst **41** incorporated into Zr(IV)-tetrahedron **C34**.

Choi and co-workers incorporated a Re(I) CO_2_ reduction catalyst [Re(dcbpy)(CO)_3_]Cl **41** as a ligand in the zirconium-based cage structure [(Zr_3_O(OH)_3_Cp_3_)_4_(**41**)(**42**)_5_)]Cl_4_ (**C34**) (Figure 26).^100^ Catalyst **41** is mixed with nonfunctionalized dcbp ligand (**42**, dcbp = 5,5-dicarboxylatebiphenyl) in a 1:5 ratio. In photochemical CO_2_ reduction with TEA as a sacrificial reductant, an average TOF of 558 h^–1^ was obtained over a period of 24 h in CO_2_ saturated MeCN. Free ligand **41** under the same conditions showed a significantly lower activity, with a TOF of 131 h^–1^ after 2 h and 12 h^–1^ for over a period of 24 h. **C34** was also compared to its analogous MOF structure UiO-67, with incorporated **41** as ligands. For the MOF, an average TOF of 27 h^–1^ was obtained over 24 h. The significantly lower activity was explained by inaccessibility of active sites that are not near the surface of the MOF particles. Mass transport limitations within the MOF for sacrificial reductant TEA led to the limited availability of electrons, thus resulting in lower activity. Both this and the previous example clearly demonstrate the advantage of using the stable, accessible cage **C34** compared to the free ligand **41** and MOF analogue.

**Figure 27:**
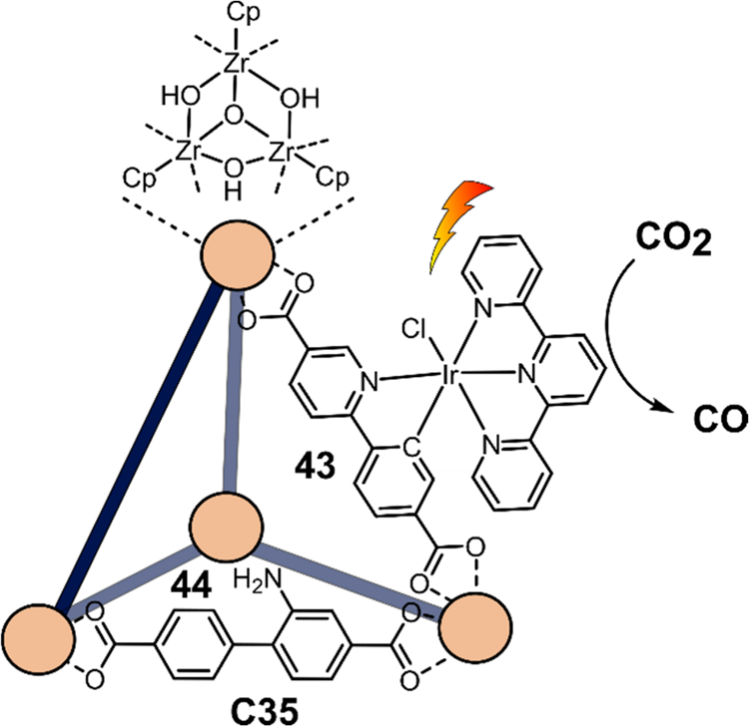
Schematic representation of tetrahedral cage **C35** based on ligands **43** and **44**, which performs photochemical CO_2_ reduction to CO in MeCN/H_2_O (4/1) with TEA as a sacrificial reductant.

Su and co-workers then incorporated [Ir(ppy)(tpy)Cl] **43** in the Zr(IV) tetrahedron by combination with biphenyl **43** to form [(Zr_3_O(OH)_3_ Cp_3_)_4_(**43**)(**44**)_5_)]Cl_4_ (**C35**) (Figure 27).^154^ Photocatalytic CO_2_ reduction with the cage was performed in MeCN using TEA as the sacrificial electron donor, reaching TON values per catalytic site of 20, with 96% selectivity toward CO. In comparison, a TON of 3.74 was obtained for molecular catalyst **43** under the same conditions. **43** shows little activity after 1.5 h, while **C35** retains 64% of its activity after 3 cycles of 5 h.

It was shown that in MeCN **C35** forms aggregates. Single **C35** cages can be obtained by dispersing the material in MeOH. Particle sizes were determined by dynamic light scattering (DLS) measurements to be 5.5 nm, which corresponds well to the value of 5.7 nm found by TEM. The CO generation using single **C35** increased the activity by 3.4-fold compared to the bulk **C35**, reaching a TON and TOF of 59 and 120 h^–1^, respectively. In order to gain insight into the role of the NH_2_ side groups of **44**, DFT calculations were performed. It was found that hydrogen bonding in the cage structure between the O of the ligating carboxylate and H of the NH_2_ stabilized the transition state, which does not occur in the mononuclear reference system due to the lack of surrounding ligands. This indicates that not only the stabilization of the catalyst enhances CO_2_ reduction but also the ligand scaffold of the cage promotes higher activity.

In the examples discussed in this section, the light-harvesting unit was installed as linker, and the metal node served as catalytic center. An alternative to this is the co-incorporation of both functions in the form of linkers. Current examples are mostly based on symmetric cages, however, heteroleptic coordination cages exhibiting multifunctionality are nowadays also accessible.^69,155,156^ Several strategies to prepare these cages have been reported; the most efficient include: (i) the use of shape-complementary ligands, (ii) donor-site engineering, or (iii) the hierarchical buildup of such cages. This type of more complicated structures allow new designs to combine light-harvesting units with catalytic centers.

## 4 Photoredox Catalysis for the Synthesis of Complex Molecules

The use of light in the synthesis of complex molecules has taken considerable interest because it can create new reaction pathways.^157^ Light can create highly reactive radical-type intermediates under mild conditions by excited-state single-electron transfer.^158−161^ In the past two decades, photocatalysis has seen a large interest from applied and fundamental research fields toward mechanistic studies and development of novel photocatalysts,^162−164^ dual photoredox catalysis,^54^ enantioselective photocatalysis,^41,165^ C–H functionalization,^53,166^ photochemical isomerizations,^167^ polymerizations,^168^ and technological development of flow chemistry.^169^ In this context, a plethora of new PSs as photoredox catalysts have been developed.^58,162,170^ In addition, new dual-type photoredox methodologies have been developed, combining photoredox events with for example organocatalysis,^171^ transition metal catalysis,^54^ electrocatalysis,^172^ and others.^173,174^ These dual-type strategies are based on generating highly reactive radical-type intermediates using light as a reagent, of which the reactivity is controlled by the introduction of the second component.

Supramolecular cages have been demonstrated to provide an interesting tool to control selectivity and activity in photoredox catalysis. Preorganization of the photoredox catalyst and the reactive molecule that quenches the excited state can be achieved inside the cage and should prevent the diffusion dependence of the PET. However, the same preorganization could also facilitate BET. In our discussion, we will focus on four strategies that have been applied to perform photoredox catalysis in the cavity of cages: (i) encapsulation of light-absorbing guests, (ii) formation of a host–guest charge transfer (CT) complex, (iii) incorporation of a PS in the linker, and (iv) incorporation of a PS in the metal node.

### 4.1 Encapsulation of Light-Absorbing Guests

**Figure 28:**
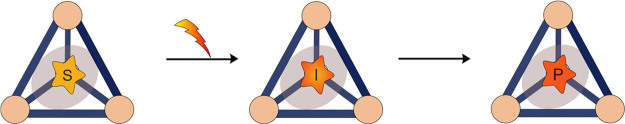
Schematic representation of an encapsulated photoactive guest in a photochemically inert host, and the reaction by excitation of the substrate (S) to intermediate (I), yielding product (P).

The unique microenvironment in the cavity of supramolecular cages has been extensively used to encapsulate various guests by means of electrostatic, hydrophobic, and van der Waals interactions.^24,175,176^ Similarly, light-absorbing molecules can be encapsulated in nonreactive hosts and undergo chemical reactions inside the cavities (Figure 28).

**Figure 29:**
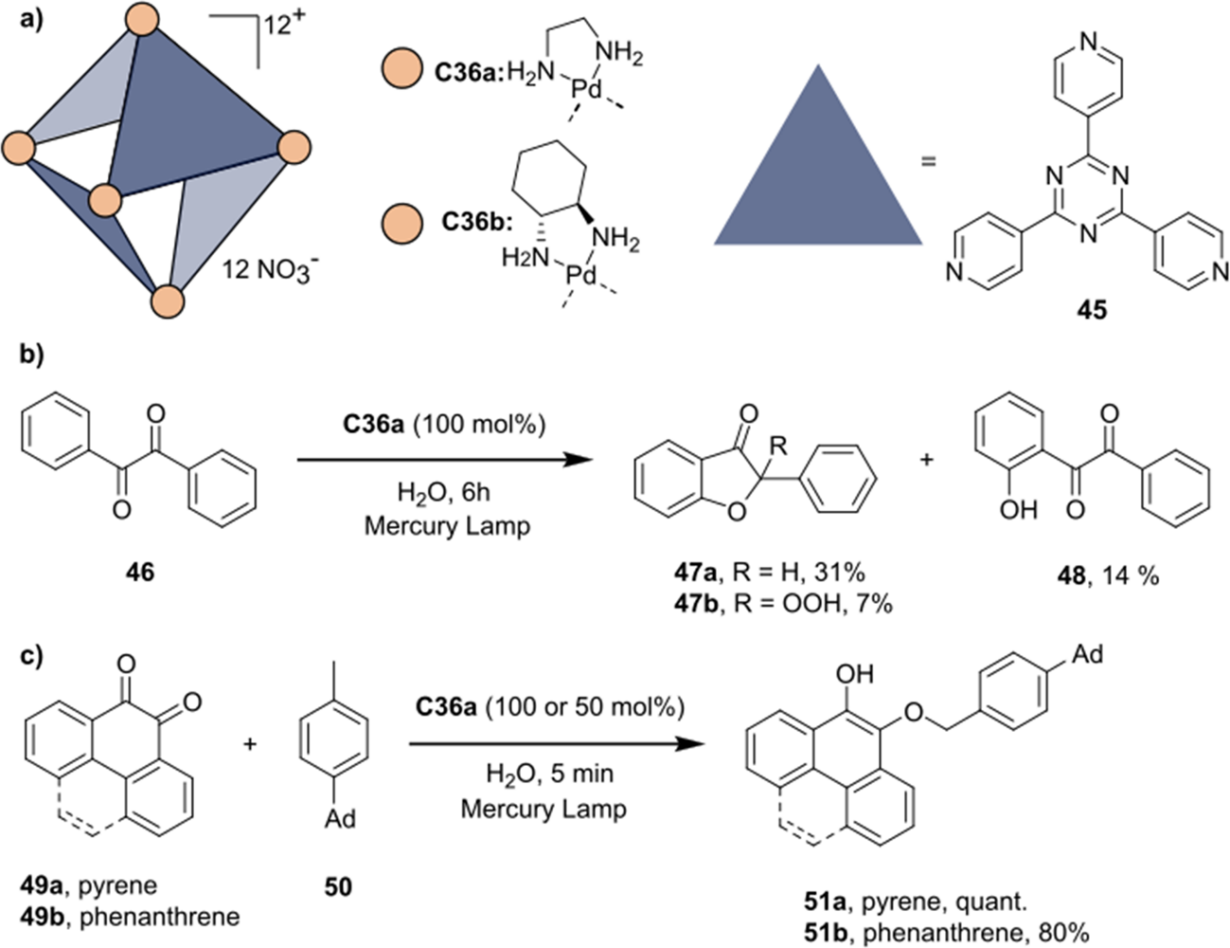
(a) Schematic structures of triazole **45** based Fujita cages with achiral (**C36a**) and chiral (**C36b**) Pd capped nodes. (b) **C36a** induced photochemical cyclization of α-diketones. (c) **C36a** induced photochemical radical coupling of quinones with benzylic carbons.^178,179^

Fujita and co-workers have applied this principle using [Pd_6_**45**_4_] (**45** = 2,4,6-tri(pyridine-4-yl)-1,3,5-triazine) cage **C36a** (Figure 29a).^177^ They found that two guest molecules of α-diketone **46** in H_2_O could be encapsulated in **C36a** (Figure 29b).^178^ After filtration of excess substrate, the newly formed host–guest complex was irradiated with a mercury lamp, forming three different reaction products: cyclized products **47a** and **47b** and OH-substituted **48**. However, in the absence of **C36a**, mainly products resulting from homolytic cleavage, such as benzaldehyde, were present in the mixture of compounds that formed. Therefore, the authors demonstrated that the cavity of **C36a** induces a spatial constraint by preorganization of the substrates that suppresses the reaction pathways that are dominant in bulk solution and enables different reactions. In addition, the same authors found that *ortho*-quinone **49a** and *p*-adamantyl toluene (**50**) in water could be selectively co-encapsulated in **C36a** in a 1:1:1 ratio.^179^ Remarkably, when only **49a** was exposed to **C36a**, barely any encapsulation was observed, indicating a strong positive cooperativity in binding. Upon photoirradiation (mercury lamp) of the host–guest complex, an hydrogen atom abstraction was proposed to lead to the formation of a benzylic radical species. Due to the encapsulation in the cage, this radical species favored the selective formation of the O-coupled 1,4-adduct **51a** (Figure 29c). The reaction in absence of **C36a** resulted in a complex mixture of products. Both of these studies show that the reactivity of the photoinduced reactions can be altered by the microenvironment created by the cavity of cages. However, because these substrates can also undergo reactions in absence of **C36a**, stochiometric amounts of cage are required for proper control of the reaction. However, using quinone **49b** instead of **49a** remarkably allowed lower **C36a** loading (50 mol %) due to rapid equilibration of the inclusion complexes to give the resulting coupled product **51b** in 80% yield under the same conditions. Yet the competition between reactions in and outside of the cavity still limit the catalytic application of this strategy.

**Figure 30:**
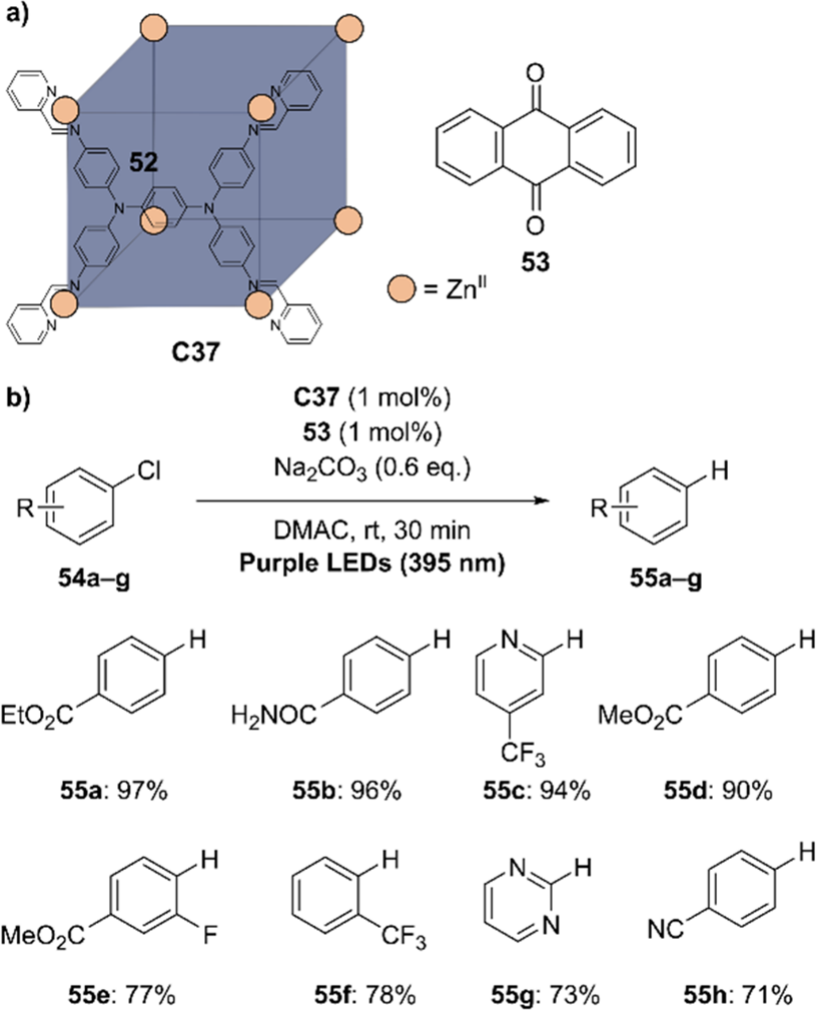
(a) Structures of cubic Zn_8_**52**_6_ C37 and anthraquinone **53** guest. (b) photochemical dechlorination of various chlorobenzene derivatives with **C37** and **53**.^180^

The group of Duan recently reported the self-assembly of [Zn_8_**52**_6_] **C37**, which contains triarylamine moieties in the cage walls (Figure 30a).^180^ The photocatalytic reactivity of the **C37** and anthraquinone **53** mixture was evaluated during the reduction of chlorobenzene derivatives **54a**–**g** (Figure 30b). Under optimized conditions, dehalogenation reactions proceed fast (30 min) in excellent to good yields of **55a**–**g** (97% to 71%). However, electron rich chlorobenzenes with higher reduction potentials could not be converted by this procedure.

Control experiments showed a significant drop of activity when **C37** was absent or replaced by either building block **52** or the Zn-salt. Na_2_CO_3_ accelerated the reaction, as was reported for similar photochemical dehalogenation reactions.^181^ Interestingly, the power of the light source showed a quadratic relation to the yield of the reaction after 20 min. This second-order dependence of photons in the rate law indicates that a two-photon process is occurring as the rate-determining step.

**Figure 31:**
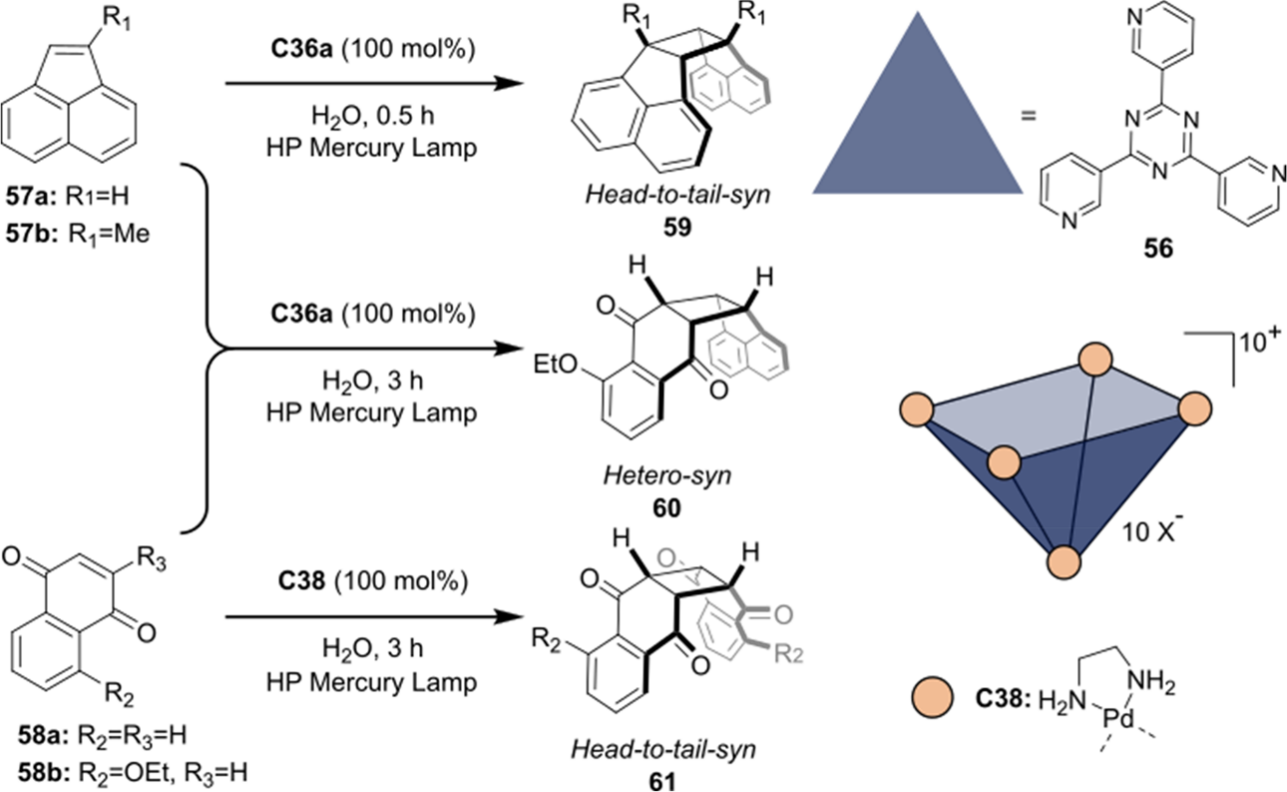
Highly selective photochemical homo- and hetero [2+2] cycloadditions in the cavity of cage **C36a** or bowl **C37**.^183^

**Figure 32:**
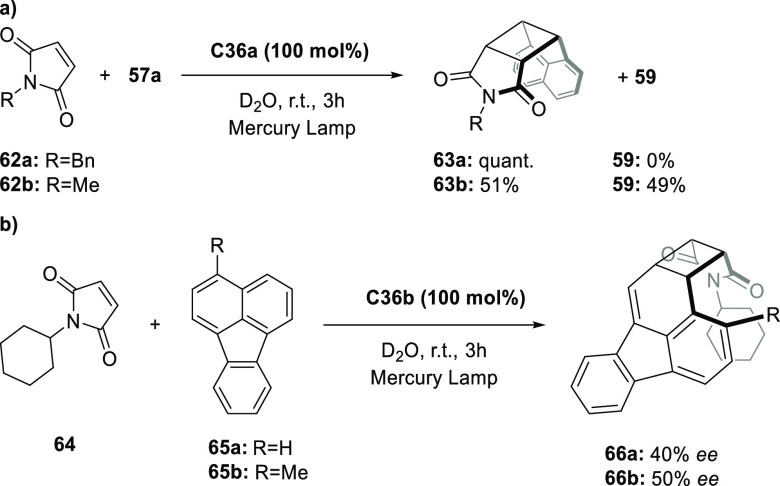
Photochemical hetero [2+2] cycloadditions of maleimides with (a) acenaphthylene **57a** in **C36a**, showing an induced fit, and (b) fluoranthene derivates **65a** and **65b** in **C36b**, providing chiral products with moderate ee’s.^184,185^

An ideal model reaction for photochemical cage catalysis is the thermally forbidden [2+2] cycloaddition. By making use of the excited state, the reaction becomes symmetrically allowed and can proceed in high conversions.^182^ Using cage **C36a** and similar bowl Pd_6_**56**_4_**C38** (**56** = 2,4,6-tri(pyridine-3-yl)-1,3,5-triazine), the Fujita group explored several [2+2] additions.^183^ Generally, an excess of alkene guests was added to the hosts in water, followed by filtration to remove unbound guests. The resulting solution was then irradiated with a mercury lamp to give the products encapsulated in the hosts, which were removed by extraction. In their first report they showed that the cavity of **C36a** gave quantitatively rise to only *syn* and head-to-tail isomers after the [2+2] additions of **57a**,**b** and **58a**,**b** (Figure 31) in both homo- and heterodimerization reactions (**59**–**61**).^183^ In the homodimerization of quinones **58a** and **58b**, the cavity of the bowl shaped **C38** appeared to position the substrates such that the (head-to-tail) *syn* products were formed in quantitative fashion. Later, they also reported the selective heteroadditions of usually photochemically inert maleimide **62a** with **57a** to give the corresponding product **63a** in high yields (Figure 32a).^184^ Host–guest interactions proved critical. Maleimide **62b** is poorly bound inside the cavity of **C36a**, and as a result homodimer **59** is also formed. Chiral cages, accessible by using Pd cornerstones based on chiral cyclohexane diamine (**C36b**), provide the opportunity to explore enantioselective photoconversion. Indeed, performing the [2+2] addition between maleimide **64** and fluoranthene **65a** or **65b** (Figure 32b) in the cavity of **C36b** provides a degree of stereo control over the photoinduced reaction (40 and 50% ee for **65a** and **65b**, respectively).^185^ Interestingly, the capping group based on chiral cyclohexane diamine only induces a small conformational change to the cavity with respect to the parent cage, yet it was already sufficient to convert the substrate with moderate ee, highlighting the potential of host induced (stereo) selectivity.

In this context, cyclodextrins are also frequently used for the [2+2] additions. Due to their natural chirality, they often provide excellent stereoselectivity when reactions are carried out inside these hollow structures, which have been used for various photochemical reactions. These examples are beyond the current scope but are nicely reviewed elsewhere.^186^

### 4.2 Host–Guest Charge Transfer Complexes

Electron–donor–acceptor (EDA) complexes may result from the interaction between electron-rich donors and electron-poor acceptors. The formation of such complexes gives rise to a new set of HOMO and LUMO orbitals resulting from their interaction. Due to the stabilizing effect of the noncovalent interaction, this may result in new charge transfer (CT) absorption bands that lie within the visible light energy. This can often be readily observed by a color change of the solution upon addition of guest to host. Irradiation of these CT bands may result in SET from the donor to acceptor, creating a radical pair. Typically, these radical pairs recombine within a few picoseconds, hampering the use in diffusion dependent chemistry.^187^ However, intercepting the radical pair with a fast chemical reaction from a preorganized substrate could provide a new paradigm for photochemistry.^188^

**Figure 33:**
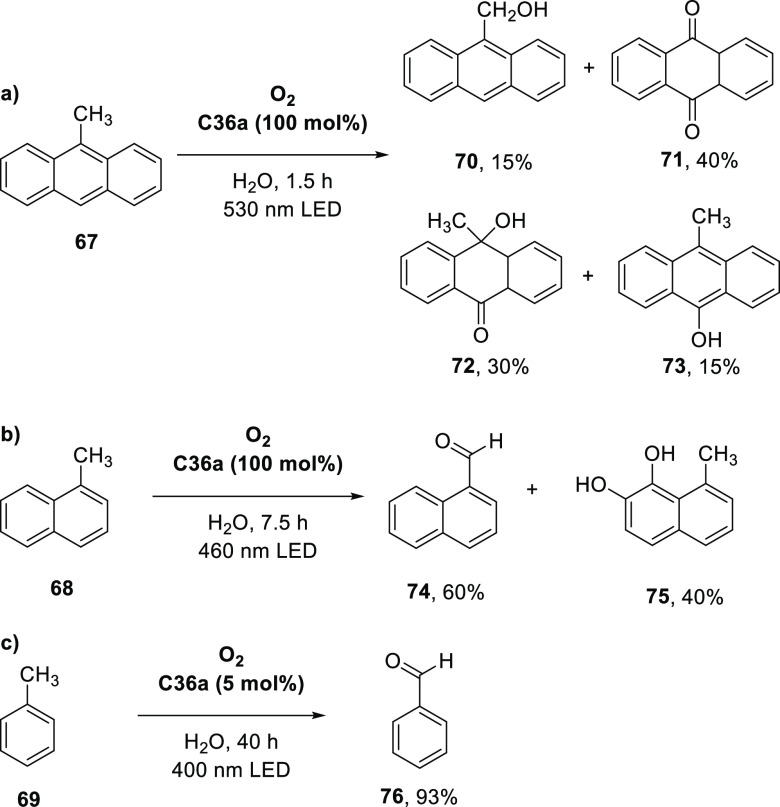
Oxidation of benzylic carbons via host–guest charge transfer complexes with **C36a** and electron rich aromatic rings (a) **67**, (b) **68**, and (c) **69**.^189^

The group of Dasgupta has investigated CT phenomena inside supramolecular cages in detail and provided evidence that such radical pairs can be intercepted. Using the Pd_6_L_4_**C36a** cage, they investigated the incarceration of electron-rich aromatic guests 9-methylanthracene (**67**), 1-methylnaphtalene (**68**), and toluene (**69**).^189^ The resulting host–guest complexes showed CT bands in the visible range, with **67⊂C36a** showing the most red-shifted absorption maximum (λ = 475 nm). Using TA measurements, the authors found that a radical cation is generated for all three guests, which decays in the picosecond range to a neutral radical species due to proton transfer with the aqueous solvent. The resulting benzylic radical then has a relatively long lifetime and, in the presence of O_2_, yields various oxidized products (Figure 33). For substrate **67**, this reaction with O_2_ can lead to substitutions in the central ring (**70**–**73**, see Figure 33a). However, for **68**, this addition is not possible, resulting in a smaller range of oxidized products (**74** and **75**, see Figure 33b). Remarkably, the photocatalyzed aerobic oxidation of toluene with 5 mol % **C36a** resulted in the selective formation of benzaldehyde (**76**) in 93% yield (Figure 33c). Using TA spectroscopy, the authors were able to study the proton transfer of the guests to the solvent. They found that in the case of **68** and **69**, the radical cation state decays with single time constants of 354 and 46 ps, respectively. However, in the case of **67**, they found two corresponding time constants for the deprotonation, namely 14 and 47 ps. The authors attributed these different time constants to two different conformations of the encapsulated guest molecules with different solvent exposure, as confirmed by temperature dependent ^1^H NMR studies. Therefore, they highlight that the specific preorganization of the substrate may be crucial for the reaction progress and to prevent BET processes.

**Figure 34:**
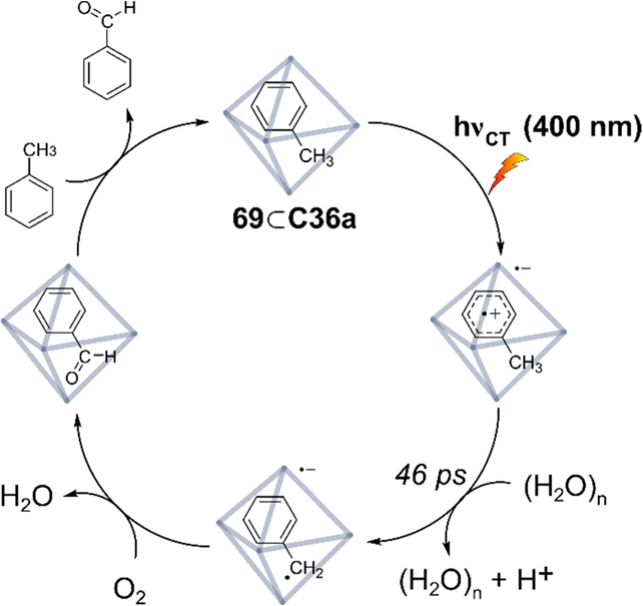
Proposed mechanism of the photoinduced oxidation of toluene to benzaldehyde by host–guest charge transfer complexes developed by Dasgupta and co-workers.^189^

The proposed mechanism of the photoinduced oxidation of toluene is shown in Figure 34. Upon host–guest complex formation, a CT band forms, which can be excited by 400 nm LEDs. This generates the corresponding radical pair, with the radical cation of toluene and radical anion of **C36a**. Before BET reproduces the ground-state system, proton transfer from the activated toluene to the aqueous solvent occurs fast (46 ps) to give the neutral benzylic radical. This radical species is long-lived and may be oxidized to the aldehyde by oxygen. The affinity for benzaldehyde in **C36a** is much smaller compared to toluene due to the increased polarity, which results in guest exchange. The decreased binding affinity in the product is crucial for catalysis in cages with low catalytic loading, which is exemplified in this oxidation.

**Figure 35:**
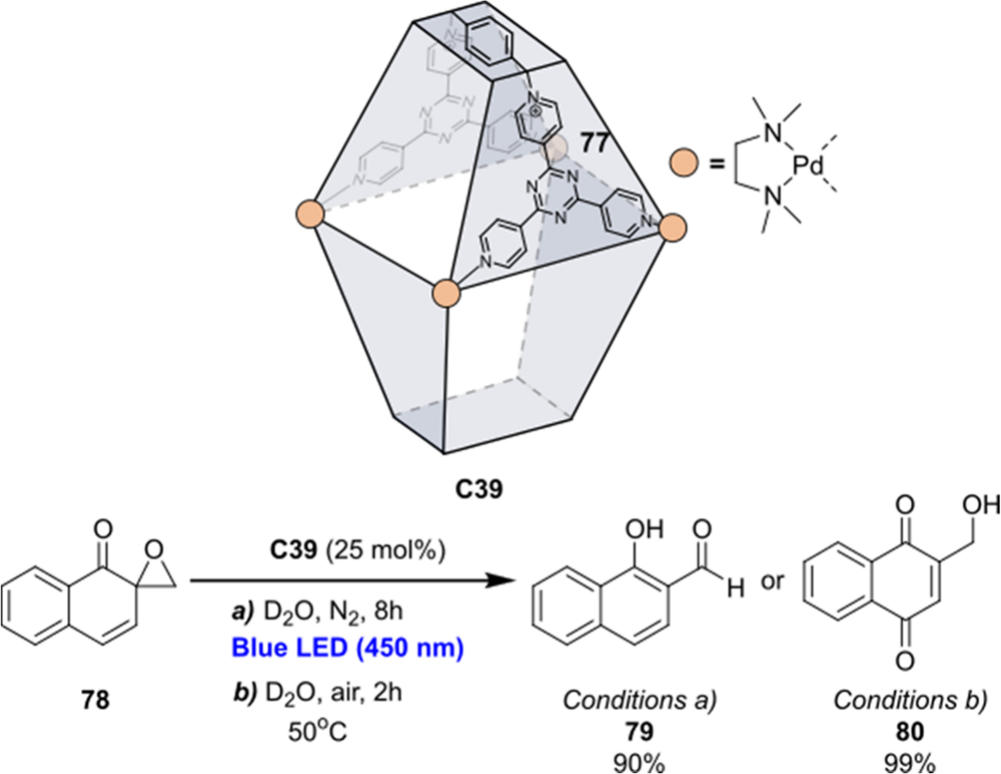
Condition-dependent reactions of spiroepoxy naphthalenone **78** in **C39**. Conditions: (a) photochemically induced epoxide ring opening to form **79**, (b) thermally induced aerobic Wacker-type oxidation to form **80**.

The group of Sun developed Pd_4_**77**_2_**C39**, containing a more spacious cavity than **C36a**, which showed promising photochromic and redox properties (Figure 35).^190^ Host–guest complexation between **C39** and spiro-epoxy naphthalenone **78** was investigated with ^1^H NMR, DOSY, and molecular mechanics computations, and the results suggested the encapsulation of up to four guests in **C39**. Interestingly, the UV–vis spectrum of the host–guest complex showed an additional CT band around 375 nm. Irradiation of the aqueous [**78**_4_**⊂C39**] with blue LEDs (λ_max_ = 450 nm, 6 W) under inert atmosphere for 8 h afforded the ring-opened aldehyde **79** in 90% yield. In the absence of **C30**, product **79** is formed in significantly lower yields. However, irradiation of **78** in chloroform with purple LEDs (λ_max_ = 390 nm) quantitatively yielded **79**, indicating that the presence of **C39** allows for the use of lower energy light due to the formation of a CT complex. Interestingly, when aqueous **(78)**_**4**_**⊂C39** is heated to 50 °C under air in the dark, a Wacker-like process occurs that provides **80** quantitatively in 2 h. Therefore, the host–guest **(78)**_**4**_**⊂C39** complex allows for a condition-controlled environment that provides either **79** under irradiation or **80** thermally.

**Figure 36:**
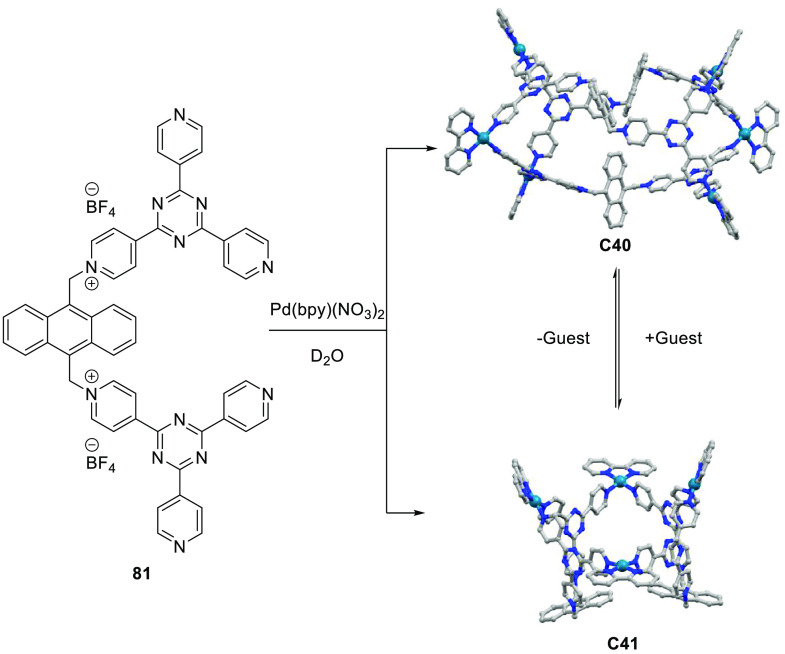
Self-assembly of guest-adaptive capsule **C40** (Pd_6_**81**_3_) and bowl **C41** (Pd_4_**81**_2_) that forms upon addition of guest to **C40**.^191^

The same group replaced the central benzene group in **77** by anthracene (**81**) in **C40** to increase the absorption properties of the cage. Interestingly, they found that exposure of flat aromatic guests such as 4-nitrothioanisole (**82a**) induce a structural change from Pd_6_**81**_3_ capsule **C40** to Pd_4_**81**_2_ bowl **C41**, as determined by ^1^H NMR studies and single-crystal X-ray diffraction measurements (Figure 36).^191^ Reversibility of this system was also demonstrated by removing the guests, resulting in the reformation of capsule **C40**. In addition to the structural change of **C40** to **C41**, the photophysical properties also changed upon guest encapsulation. A color change from yellow to red occurred upon exposure of **C40** to a solution of 10 equiv of 4-methoxythioanisole (**82b**) in D_2_O, which was shown by UV–vis studies to be an enhanced blue-light absorption (λ > 425 nm). These absorption changes were attributed to the formation of a CT band between the electron-rich guest and the electron-poor triazine moiety of the host.

**Figure 37:**
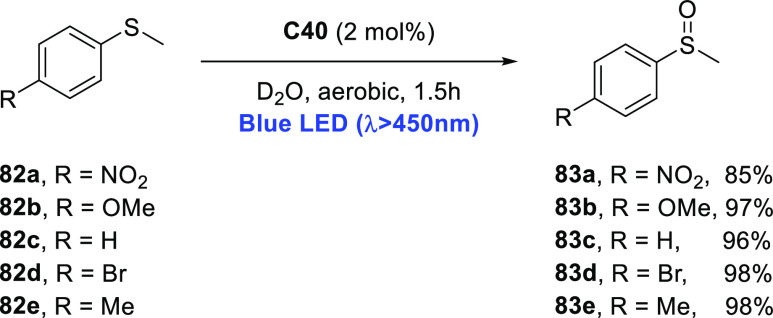
Photocatalytic oxidation of thioanisoles to corresponding sulfoxides by guest-adaptive capsule **C40**/bowl **C41** hosts.^191^

The group of Sun performed photocatalytic oxidations with oxygen from the air by irradiating a solution of thioanisoles (**82a**–**e**) and capsule **C40** with blue LEDs (Figure 37). This efficiently yielded the corresponding sulfoxides (**83a**–**e**), whereas overoxidation to the sulfone barely occurred (≤1%). Control experiments revealed that the reaction does not proceed in absence of cage, air, or light.

**Figure 38:**
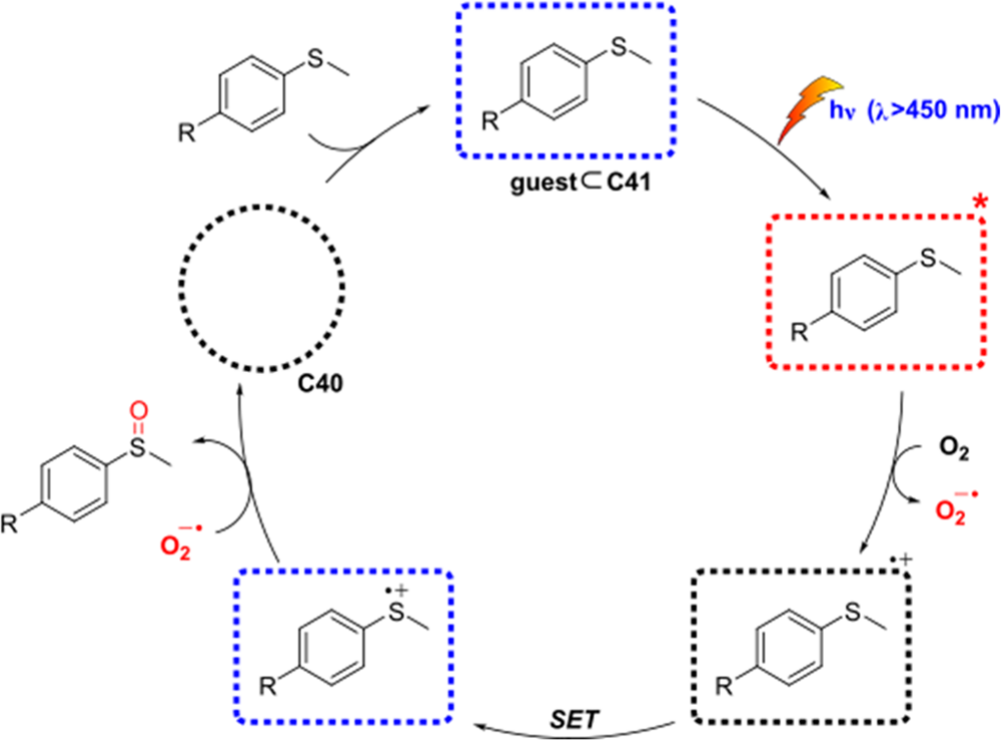
Proposed mechanism of the photoinduced oxidation of sulfides to sulfoxides catalyzed by guest-adaptive capsule **C40**/bowl **C41** hosts. Circles indicate capsule **C40** and squares indicate bowl **C41**.^191^

To shine light on the nature of the oxidant, the authors added NaN_3_ (^1^O_2_ quencher) and benzoquinone (O_2_^.–^ capturing agent) to the reaction mixture. They found that NaN_3_ does not inhibit catalysis, whereas the addition of benzoquinone results in significantly reduced catalytic activity. Based on these experiments, the authors concluded that the superoxide anion is responsible for the oxidation of the thioanisoles. Therefore, authors proposed a mechanism for this photocatalytic oxidation (Figure 38), which first involves binding of the guest in capsule **C40**, inducing structural change to bowl **C41**, subsequently, visible light excitation results in effective electron transfer by the host–guest complex to facilitate the oxidation of sulfides to sulfoxides by the superoxide anion. Then, due to the increased hydrophilicity and poor shape complementarity of the sulfoxides, the product is replaced by a new substrate. Finally, after full conversion of the sulfides, capsule **C40** can be recovered due to the poor binding of sulfoxides in either capsule **C40** or bowl **C41**. The authors thus presented an elegant enzyme-mimicking photocatalytic system that undergoes structural and photophysical changes upon guest binding, however, the exact function of the CT complex is not yet fully clarified.

**Figure 39:**
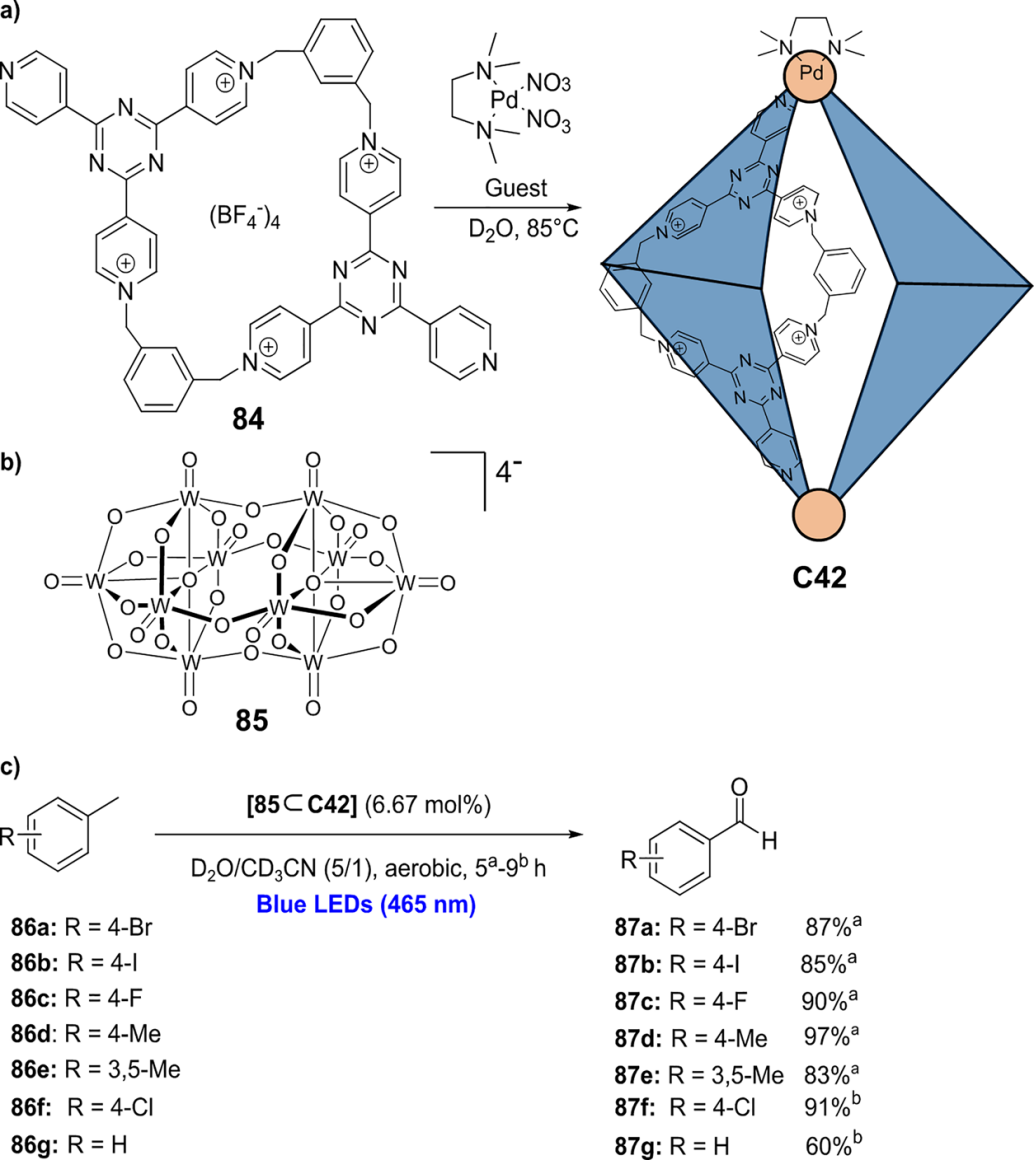
(a) Guest-assisted self-assembly of **84** to form [Pd_2_**84**_2_] cage **C42** in quantitative fashion. (b) Photoredox active guest W_10_O_32_**85**, which may be used as templating guest for the quantitative formation of [**85⊂C42**]. (c) Photocatalyzed aerobic oxidation of toluene derivatives with [**85⊂C42**] under blue LED irradiation. (*a*) reaction time = 5 h. (*b*) reaction time = 9 h.^192^

In a recent study, the group of Sun studied the self-assembly of flexible cage Pd_2_**84**_2_**C42**, based on macrocycle **84**.^192^ Self-assembly gave rise to a mixture of complexes in absence of templating guests, but the presence of a template such as decatungstate **85** resulted in the clean formation of [**85⊂C42**] self-assemblies (Figure 39a). Interestingly, by adding **85** (Figure 39b) as a template, quantitative formation of [**85⊂C42**] was observed based on ^1^H NMR, ESI-MS, and XRD analyses. According to the crystal structures, the cavity of **C42** adapts its size, depending on the nature of the guest. Because **84** is flexible due to presence of the CH_2_-linkers, there is still space inside the cavity of [**85⊂C42**] to bind more guest molecules. Moreover, a CT interaction between host **C42** and guest **85** was evident from the UV–vis spectrum, which allows for the excitation at longer wavelength light (λ = 465 nm) than is possible for free **85**. The authors found that toluene derivatives **86a**–**g** undergo oxidation by air to the respective aldehydes **87a**–**g** in good yield and with high selectivity (Figure 39c). Even the di- and trimethyl substituted derivatives (**87e** and **87f**) mainly underwent a single oxidation (87% and 83%, respectively), although the di- and trialdehydes were also present in the product mixtures. Control experiments revealed that only **84**, **C42**, or **85** cannot perform photocatalysis under the used conditions because they do not absorb the used 465 nm light. However, when **84** and **85** are exposed together to the photocatalytic conditions, the aldehyde is produced (36% yield), indicating that a charge transfer interaction is occurring. Thus, the design of flexible cage **C42** allows for the encapsulation of large guests, such as polyoxometalates (e.g., **85**), which opens new opportunities toward the encapsulation of these interesting materials that are used frequently in photocatalysis and as redox mediators.^193−195^

### 4.3 Photoactive Cages

The design of supramolecular coordination cages that possess light-absorbing functionalities is interesting for molecular sensing and photocatalysis. Toward these ends, several cages have been synthesized that either contain light-absorbing bridging ligands or metals ions.^37,39^ With these self-assembled structures in hand, photocatalysis should be feasible in a controlled and efficient manner due to the spacial constraints presented by the cages. Additionally, the photophysical properties undergo changes when the PSs are placed in close and constraint proximity, which have recently been reviewed.^196^ In this section, our discussion will focus on the application of these systems in organic photoredox catalysis.

#### 4.3.1 Photosensitizer Installed on the Linkers of the Cage

**Figure 40:**
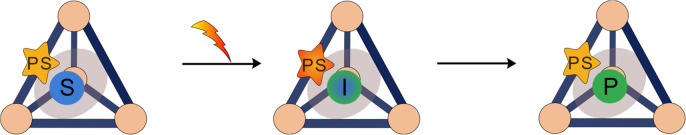
Schematic representation of a supramolecular cage containing a photoactive linker. The excitation of the photosensitizer (PS) starts the reaction by transforming substrate (S) to intermediate (I), yielding product (P).

One way of producing a light-absorbing supramolecular cage is by selecting desired organic linker groups. The cavity presented by the cage can be used to control substrate concentration as well as provide preorganization during photochemical reactions performed with such cages (Figure 40).

**Figure 41:**
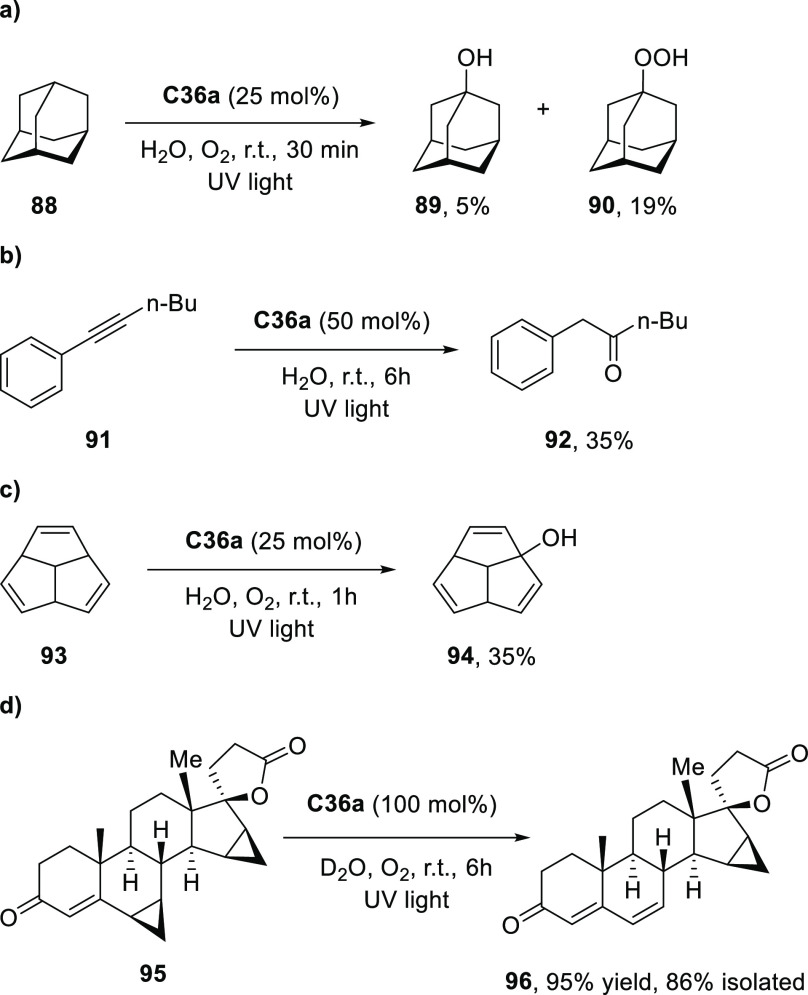
Photochemical reactions in cage **C36a** upon irradiation of the corresponding host–guest complex with UV light (a) oxidation of adamantane, (b) hydration of aryl alkynes, (c) oxidation of triquinacene, and (d) demethylenation of steroid **95**.^197−200^

In 2004, the group of Fujita were the first to show that photoactive cages allowed light-driven chemical reactions after sensitization. Their **C36a** cage showed absorbance in de UV-region (λ<370 nm) and was able to encapsulate photo inert adamantane (**88**) in D_2_O. Irradiation of the resulting host–guest [**88**_4_**⊂C36a**] under aerobic conditions yielded oxidized products **89** and **90** in a total of 24% yield (Figure 41a). This regioselective oxidation thus showed one turnover per host molecule, indicating that catalytic turnover was not achieved. Interestingly, irradiating the host–guest complex in absence of O_2_ resulted in the formation of **89** and a long-lived blue solution, likely resulting from the one-electron-reduced host. This was substantiated by the EPR spectrum of the blue solution, which contained a radical species (*g* = 2.002) at cryogenic and room temperatures, indicative of an organic radical. To elucidate the nature of the oxidant, the authors performed ^18^O-labeling experiments with either H_2_O or O_2_, which both resulted in the incorporation of ^18^O in the products. The authors thus concluded that the mechanism must include the electron transfer from **88** to **C36a** after excitation, generating the radical pair. However, the radical cation of **88** may then react with either O_2_ or H_2_O to give rise to **90** or **89**, respectively, whereas **C36a**^**.–**^ is only regenerated in the presence of O_2_. However, a clear conclusion on the reason for the single turnover was not reported.

Similarly, cage **C36a** was used by the same group for photochemically induced anti-Markovnikov hydration of aryl alkynes.^198^ They found that aryl alkyne **91** in D_2_O formed a host–guest complex [**91**_2_**⊂C36a**] that undergoes a light-induced hydration (Figure 41b). Similarly to the previously discussed approach, cage **C36a** is excited by irradiating UV light (λ < 370 nm), after which a PET occurs with the substrate. According to the authors, **C36a** accepts an electron from **91**, which is then attacked at the anti-Markovnikov position by H_2_O from the solvent. The anti-Markovnikov selectivity was high, as the generated radical at the benzylic position is more stable. BET from **C36a** to the benzylic radical intermediate, paired with a protonation, resulted in formation of the benzyl ketone **92**. It is noteworthy that benzyl ketones are unstable under UV conditions, often resulting in α-cleavage of the carbonyl group. However, **C36a** acted as a protective shell, preventing undesired further reactivity of the product.

The group of Fujita showed that also triquinacene (**93**) could undergo selective oxidation in the presence of **C36a** under similar conditions (Figure 41c).^199^ The authors found **94** as the sole reaction product after UV irradiation of the host–guest complex [**93**_4_**⊂C36a**] for 1 h in aerobic environments. The authors found that the reaction does not proceed under anaerobic conditions, thus nominating dissolved O_2_ as the oxygen source. The proposed mechanism is similar to the previously discussed photoreactions, starting with excitation of **C36a**, resulting in PET from **93** to **C36a**. The radical cation is then intercepted by O_2_ to give the peroxy-radical, which is degraded to the more stable alcohol in **94**. Whereas the previous reactions showed no more than one turnover per cage, a yield of 35% was reported in this case, showing promise for catalytic conversion.

In 2019, the Fujita group demonstrated that using **C36a** also resulted in higher turnovers.^200^ They found that aryl- or vinyl-substituted cyclopropanes such as steroid **95** could be demethylenated in the cavity of **C36a** (100 mol %) under UV light irradiation in high yields (Figure 41d). After full conversion of the starting material, demethylenated **96** was found to be present in 95% ^1^H NMR yield and was isolated in 86% yield. Besides **96**, also hydrated formaldehyde CH_2_(OD)_2_ was found to be present in the reaction mixture. The authors expanded the scope to two vinyl- or aryl-substituted cyclopropanes, which also underwent the desired transformation in good yields. Mechanistic studies showed that a similar mechanism is operating as previously described. However, the authors showed that NO_2_^–^ was produced by performing Griess’ method,^201,202^ whereas the presence of O_2_ was not required for the reaction to occur. Therefore, they proposed that the cyclopropane ring opening is assisted by a NO_3_^–^ counteranion, which is subsequently reduced by the cage to the NO_2_^–^ anion. The authors showed that the resulting NO_2_^–^ counterion containing **C36a** could be used a total of 10 times by adding more substrate each time. After consumption of 10 NO_3_^–^ counterions, no more turnovers could occur due to product inhibition. Therefore, TON = 10 could be achieved due to the stoichiometric consumption of the counterions.

**Figure 42:**
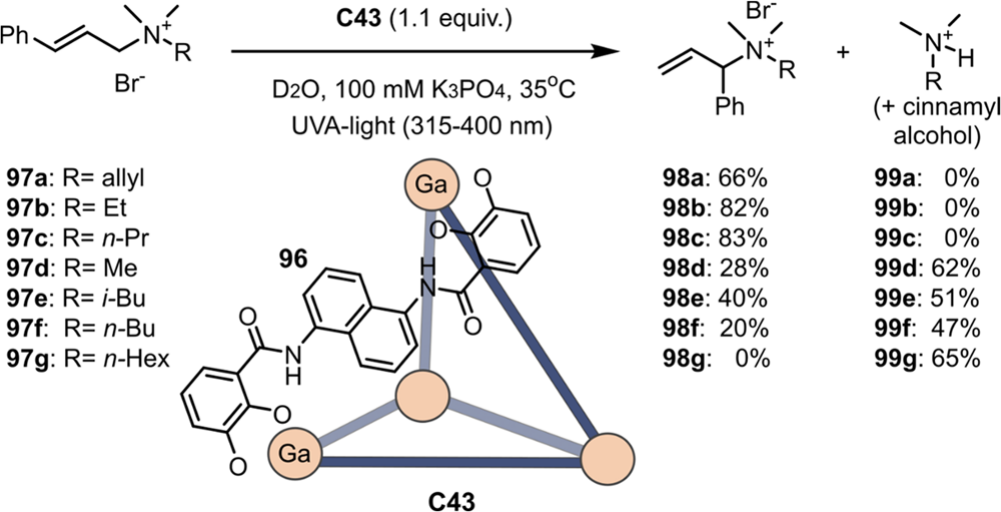
Photoinduced 1,3-rearragement of phenyl allylic quaternary amines inside the cavity of K_12_ Ga_4_**96**_6_ (**C43**) cage.^203^

**Figure 43:**
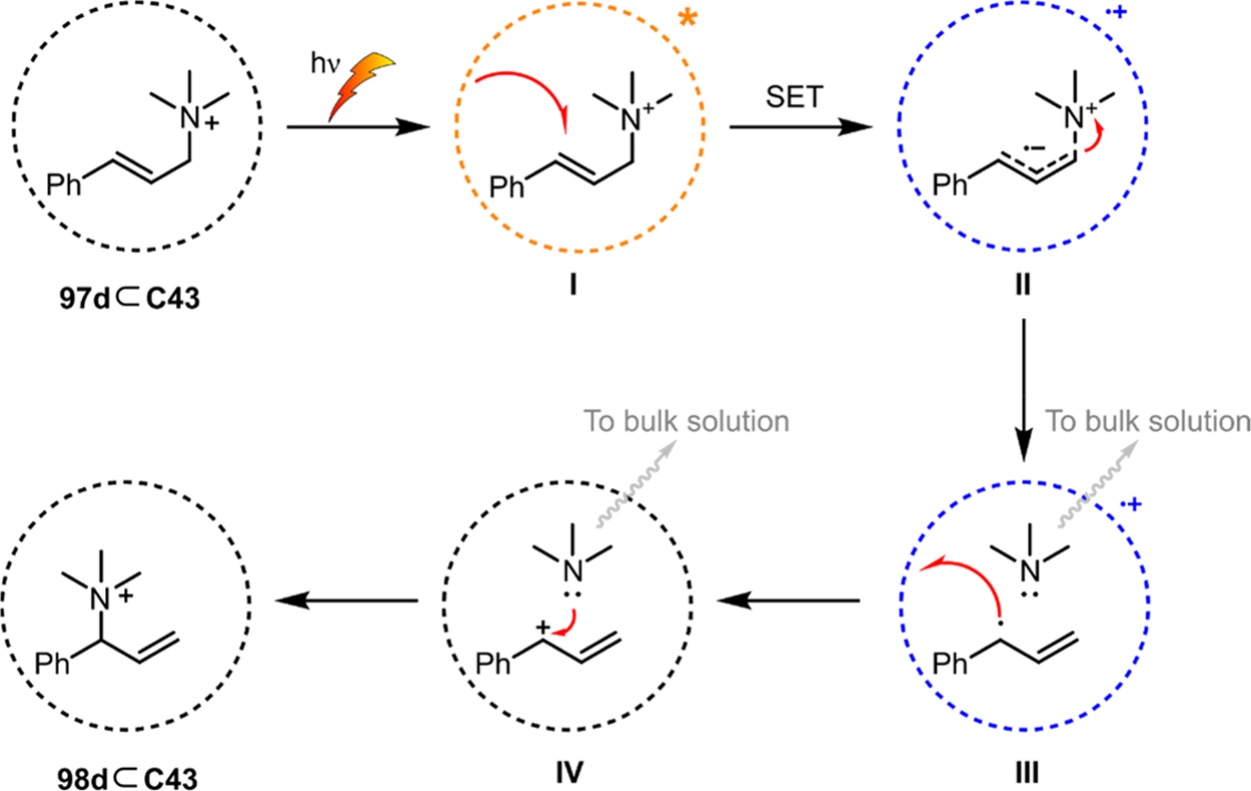
Proposed mechanism for the photoinduced 1,3-rearrangement of cinnamyl ammonium ions in the cavity of **C43**. Diffusion out of the cavity is indicated by gray arrows.

The group of Raymond have used a similar strategy to carry out photoreactions within their [K_12_Ga_4_**96**_6_] (**C43**) cage (Figure 42).^203^ The cage absorbs UV-A light (325 nm), which could induce photo processes with a suitable substrate. Because **C43** is anionic, it efficiently encapsulates cationic species such as cinnamyl ammonium ions (**97a**–**g**). Instead of the hypothesized [2+2]-cycloaddition of model substrate **97a**, the authors observed a 1,3-rearrangement upon irradiation with UV-A light under reaction conditions to give **98a**. This rearrangement does not occur when the cavity of **C43** is occupied if the cage is absent or if no light is present, indicating that a photoinduced reaction occurs inside the cavity of **C43**. The authors observed that the binding affinity plays a key role: when log(*K*_int_) > 2 (**97a**–**c**), no side products are observed, however, when log(*K*_int_) < 2 (**97d**–**g**), cinnamyl alcohol and tertiary amines (**99d**–**g**) are formed. The authors showed with use of TA, UV–vis, and fluorescence measurements that indeed **C43** is excited by UV-A-light, followed by PET to the encapsulated substrate. Based on this, the authors postulated that this PET occurs in **I** from host to guest (Figure 43), generating the corresponding radical pair in **II**. Then, the amine functionality is released, forming a stabilized allyl radical in **III**. Then the amine can either stay encapsulated or escape the cavity, depending on the binding affinity. The allylic radical in **C43** performs BET, leaving an allyl cation **IV**, which is either attacked by the amine to yield the corresponding product **98d⊂C41**. Interestingly, if the amine is released from the cavity, solvent water may attack the allyl cation but leads to the formation of cinnamyl alcohol, i.e., the nucleophilic attack on the outer carbon.

**Figure 44:**
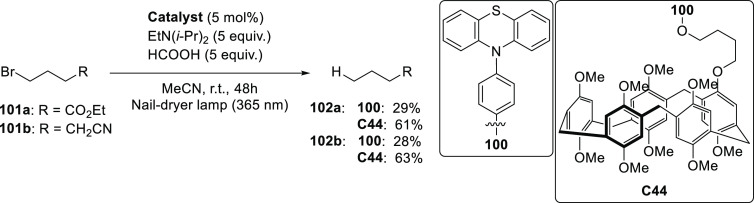
Comparison between preorganized and nonpreorganized photocatalyzed reductive dehalogenations of alkyl bromides with phenothiazines.^204^

More recently, pillar[5]arenes were functionalized with a *N*-phenyl-phenothiazine (**100**) photoredox catalyst by Schmidt and Esser (Figure 44).^204^ Deprotecting a single methoxy group to give a phenol moiety opened up **100** functionalization on the pillar[5]arene to give cavitand **C44**. This functionalized cavitand showed increased photoredox activity during reductive dehalogenations of alkyl bromides.^205^ With Stern–Volmer fluorescence quenching studies, the authors showed that the excited state of **C44** is quenched more efficiently by **101a** and **101b**, in comparison with **100**. In addition, the authors also performed catalytic experiments with substrates that could not bind in the pillar[5]arene cavity. In that case, no significant yield difference was observed between **C44** and **100**, indicating that the cavity induces substrate preorganization, which accelerates the reaction. Similarly, the pillar[5]arenes were also used in polymer-based heterogeneous photoredox catalysis and provided substrate selectivity for substrates that bound in the cavity.^206^ Therefore, it seems that the cavity of pillar[5]arenes does not change the reaction outcome of the bound substrates, but it does show increased efficiency for the photochemical conversion.

**Figure 45:**
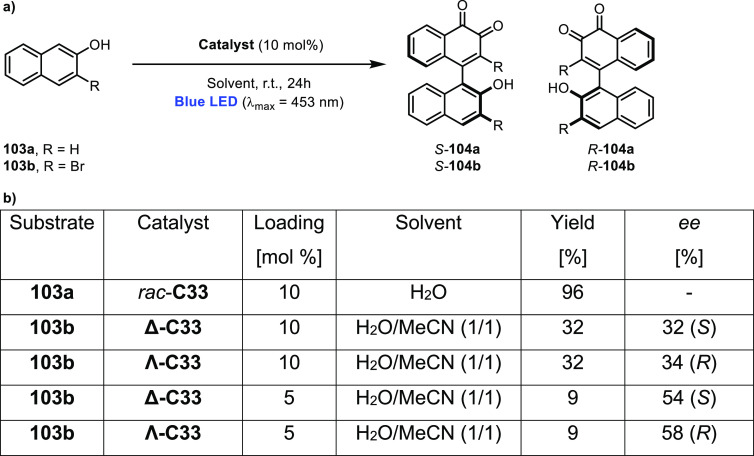
(a) Oxidative dimerization of 2-naphthol derivatives promoted by **C33** in regio- and stereoselective fashion. (b) Reaction conditions and outcomes of the oxidative dimerization of 2-naphthol derivatives.^207^

The group of Su has shown that photoreactions can be carried out with stereoselectivity in the cavity of cages.^207^ They designed ruthenium based **C33**, which can be made enantiopure (**Λ**-**C33** and **Δ**-**C33**) depending on the absolute conformation of the Ru(II) centers, which has been shown to promote [2+2] cycloadditions and H_2_ formation.^150,208,209^ The authors found that naphthol derivatives (**103a** and **103b**) could be encapsulated by **C33**, as was evident from the ^1^H NMR (titration) experiments which suggest that up to ten guests can be encapsulated by **C33**. Upon irradiation of a [**103a⊂C33**] solution in H_2_O under aerobic conditions with blue LEDs, oxidative dimerization to **104a** occurred quantitatively (Figure 45a). The authors showed that **C33** could be recycled after extraction of the product from the cavity. Dimerization with free ligand **39** in MeOH or with **3a** in water were inefficient (<23% yield), indicating that the encapsulation of **103a** promotes the dimerization to **104a**. However, **104a** rapidly racemized in solution and, therefore, bromo-substituted **103b** was selected to determine the stereoselectivity of the reaction. Due to poor solubility of **103b** in water, a mixture of MeCN:H_2_O (1:1 v/v) solution was selected as the reaction medium. Photocatalysis with **Δ**-**C33** preferentially formed the *S*-**104b**, whereas **Λ**-**C33** gave mainly *R*-**104b** in modest yields and ee, which varied with the loading of **C33** (Figure 45b). A lower loading (5 mol %) of **C33** afforded better enantioselectivity because it ensures that a sufficient amount of substrates **103b** are bound in the cavity. However, the overall conversion decreased due to a lower loading because there are more free substrates **103b** that react inefficiently. Clearly, the chirality induced by homochiral **Λ-C33** or **Δ-C33** can be transferred, likely due to preorganization of the substrates inside the homochiral cavity. Therefore, the authors established the first photoactive chiral cage that is able to promote regio- and stereoselectivity in photoredox conversions of encapsulated guests.

**Figure 46:**
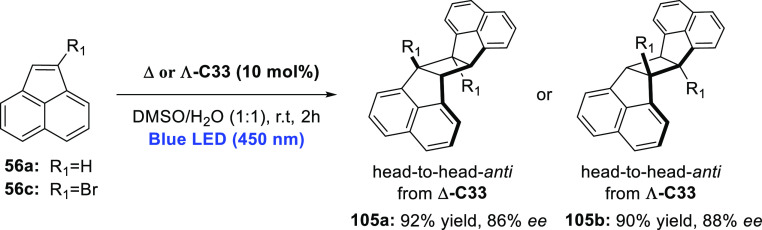
Enantioselective [2+2] cycloaddition of acenaphthylenes **56a** and **56c**, induced by the cavity of **Δ-C33** or **Λ-C33** to give the optically active head-to-head anti-dimers.

Cage **C33** was also shown to promote the selectivity of [2+2] cycloaddition reactions.^209^ As a model reaction, the dimerization of acenaphthylenes **57a** and **57c** was studied (Figure 46). Besides high diastereoselective control using a racemic mixture, with enantiopure **Δ-C33** or **Λ-C33** enantioselective control was also achieved to form the desired *anti*-head-to-head stereoisomers **105a** and **105b** in up to 88% ee. Kinetic studies revealed that the rate of the photochemical reaction is enhanced by an order of magnitude, when compared to a molecular catalyst. The enhancement may be attributed to the pre-organization effect of the molecular cage. Additionally, TA spectroscopy was used to reveal three processes in the picosecond range: (i) ISC to the triplet state in 0.28 ps, (ii) intraligand charge-transfer (ILCT) in 8.54 ps, and (iii) EnT from C33* to **56a** in 145 ps. This EnT rate again proved to be an order of magnitude faster than that of free Ru-based ligand **39**, which supports the cage effect on rate enhancement.

**Figure 47:**
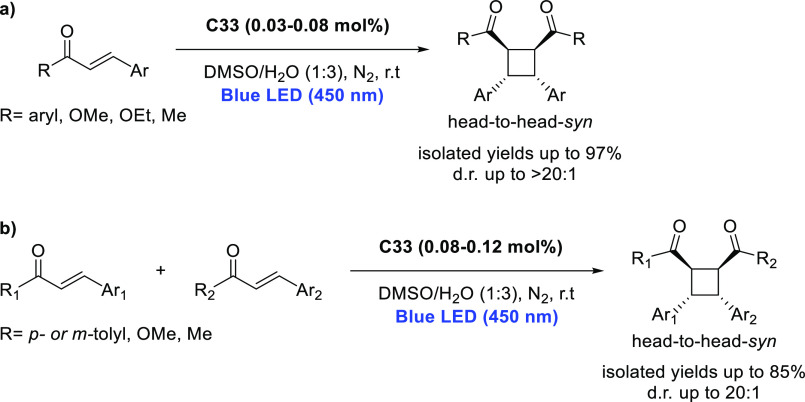
Regioselective photocatalyzed [2+2] cycloadditions by racemic **C33** for (a) homocoupling and (b) heterocoupling.

The group of Su then explored the scope of the [2+2] cycloaddition in the cavity of **C33**.^208^ Homocoupling [2+2] additions showed a high tolerance for α,β-unsaturated ketones and esters containing aromatic substituents on the β position. Generally, the transformations occurred preferably to form the *syn*-isomer in high diastereoselective fashion (up to >20:1) and with isolated yields up to 97% (Figure 47a). Heterocoupling also occurred for similar substrates in similar diastereoselectivity (up to 20:1) in moderate to good yields (40–85%) (Figure 47b). However, the reported scope was limited and may be attributed to two factors: (i) the inherent molecular suitability of the reactants to undergo heterocoupling, and (ii) the coencapsulation efficiency of the two reactants in the supramolecular host. Therefore, the steric and electronic properties of the substrates play a crucial role in the outcome of the photocatalyzed [2+2] heterocoupling.

By making use of a cobalt-based molecular cluster with sulfonylcalix[4]arenes, the group of Cui incorporated light-absorbing anthraquinone derivatives in the backbone of self-assembled cages.^210^ These cages were also able to promote [2+2] cycloadditions of several chalcones with a preference for the head-to-head *syn*-diastereomer. Although the degree of diastereoselective control was moderate (up to 13:1), it showed significant improvement over the control experiments (7.8:1). This work shows that organic photosensitizers can also be utilized for effective [2+2] cycloadditions inside the cavity of a supramolecular sphere.

#### 4.3.2 Photosensitizers Installed on the Metal Nodes of the Cage

**Figure 48:**
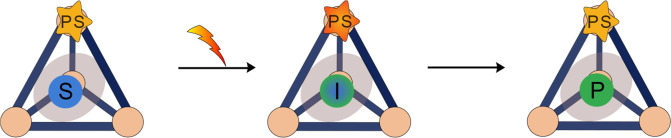
Schematic representation of a supramolecular cage containing a photoactive metal node. The excitation of the photosensitizer (PS) starts the reaction by transforming substrate (S) to intermediate (I), yielding product (P).

Transition-metal-based photoredox catalysts are often used, and their incorporation in cages has been established.^39^ Subsequently, supramolecular cages have been developed that contain these photoredox catalysts as their metal nodes. Performing photocatalytic conversions in their cavities makes use of the emergent properties which may result in increased activity, new reactivity, and induced selectivity (Figure 48).

**Figure 49:**
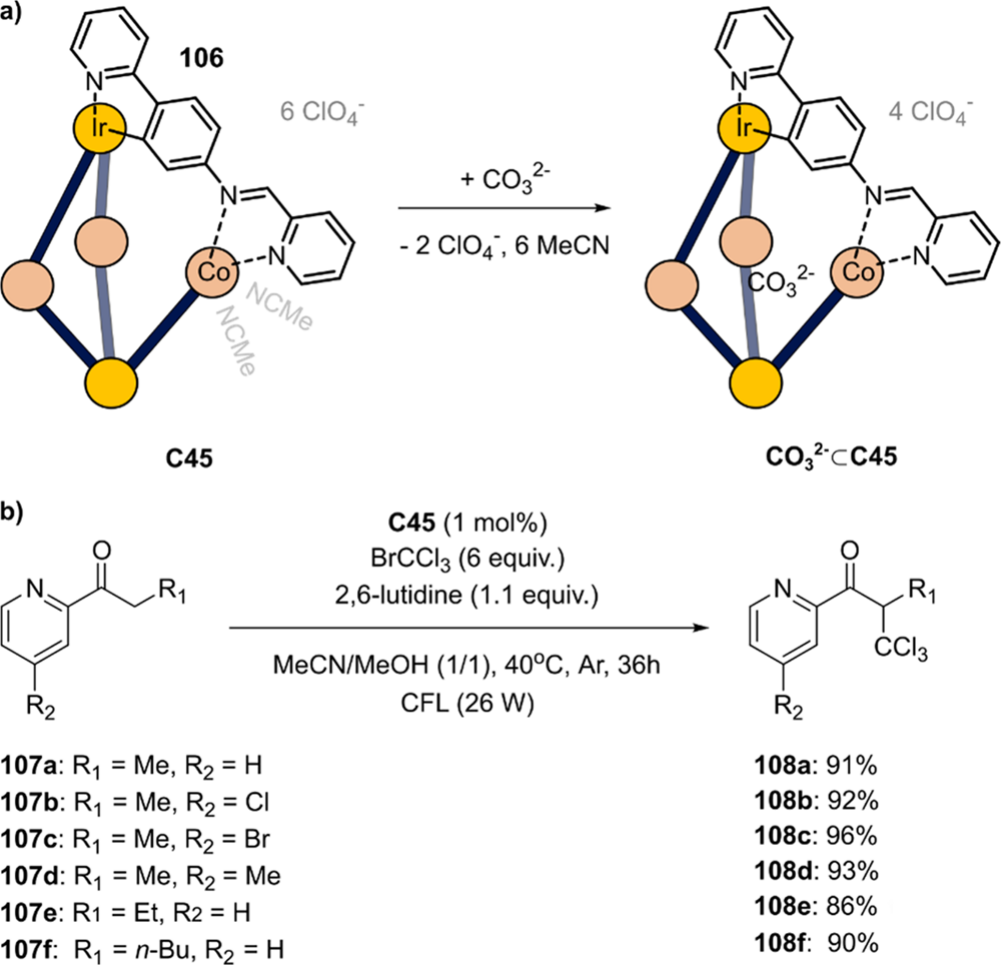
(a) Cage to cage conversion of cage **C45** induced by carbonate anions in solution. (b) Photocatalytic activity of **C45** for the trichloromethylation on the α-carbonyl position of 2-acylpyridines.^211^

The groups of He and Duan developed heterometallic Ir_2_ Co_3_ cage (**C45**) based on common photoredox catalyst *fac*-Ir^III^(ppy)_3_ (Figure 49a).^211^ Self-assembly with 2 equiv of the NH_2_ substituted *fac*-Ir^III^(ppy)_3_, 6 equiv of 2-formylpyridine and 3 equiv of Co^II^(ClO_4_)_2_ in acetonitrile afforded **C45**. Two labile MeCN ligands surround each Co(II) atom in **C45**, which can be easily exchanged to afford vacant sites and give **C45** dual functionality character. Upon addition of CO_3_^2–^ to a solution of **C45**, the MeCN ligands are released and carbonate is encapsulated to form [**CO**_**3**_^**2–**^**⊂C45**].

Photocatalytic trichloromethylation of 2-acylpyridines **107a**–**f** to the respective products **108a**–**f** could be achieved with **C45** in high yields (Figure 49b).^211^ Control experiments showed that the individual components did not result in significant conversion of model substate **107a**. Based on this, the authors propose that the Co^II^ sites should be in close proximity to the photosensitizers for efficient turnover, highlighting the dual-character of **C45**. Interestingly, **CO**_**3**_**⊂C45** did not result in significant catalytic activity (6% yield of **108a**) under the same conditions. The authors therefore designed a photoactive cage with dual-functional character that is responsive to external CO_3_^2–^.

**Figure 50:**
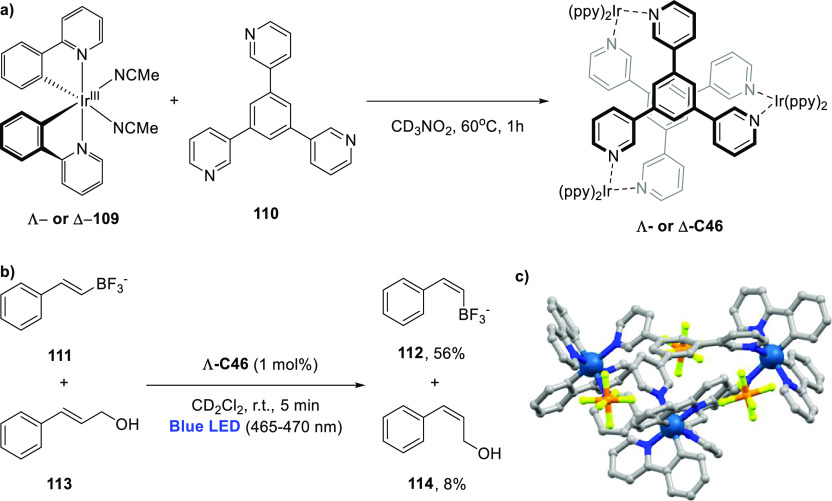
Enantiopure **C46** cage (a) self-assembly, (b) substrate selectivity of the photocatalytic *E*–*Z* isomerization between **111** and **113** by **Λ-C46**. (c) Crystal structure of **Λ-C46** atoms: C = gray, F = lime green, N = dark blue, P = orange, Ir = navy blue.^212^

The group of Fujita also developed a cage using the Ir^III^(ppy)_3_ catalyst as metal node. Self-assembly of 3 equiv of enantiomerically pure Ir-precursor **109** with 2 equiv of tripodal ligand **110** afforded **Λ**- or **Δ**-**C46** (Figure 50a). Although a cavity is formed in the center, it appears to be too narrow to bind a guest. Instead, three binding pockets near the ppy ligands are formed at the edges of the complex, which bind anions such as PF_6_^–^. These pockets were used to perform a photoinduced *E*–*Z* isomerization of *E*-trifluoro styryl borate **111** to the *Z*-isomer **112** Within 5 min, the photo stationary state (*E*:*Z* = 44:56) was reached. Substrate selectivity was also demonstrated by using the anionic **111** and the neutral analogue **113** (which produces **114**, see Figure 50b). It is, however, still unclear what the effect of **C46** on the stereoselectivity is of transformations in the small (see Figure 50c) binding pockets.

#### 4.3.3 Supramolecular Cages as Light-Harvesting Antennae

**Figure 51:**
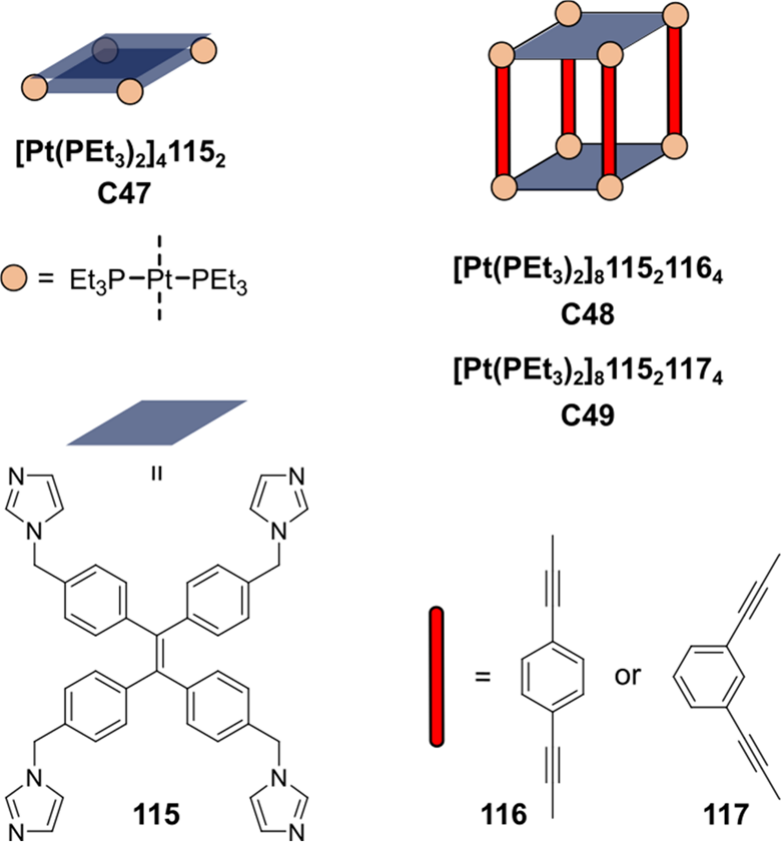
Self-assembled coordination cages **C47**–**C49** containing **115** units investigated for aggregation induced emission.

Besides using coordination cages as hosts to encapsulate substrates, they can also serve as artificial mimic of natural light-harvesting antennae. The group of Mukherjee developed a series of prismatic Pt-based cages with tetraphenylethylene (**115**) top and bottoms and different pillar linkers 1,4-diethynylbenzene (**116**) and 1,3-diethynylbenzene (**117**) (Figure 51).^213^ All three cages (**C47**–**C49**) were characterized by ^1^H-, ^31^P NMR, DOSY, ESI-MS, and DFT, which indicated the formation of a single product that corresponded to the desired stoichiometry. Although **115** does not show luminescence in solution, it has a strong aggregation induced emission (AIE).^214−216^ This is caused by the restricted rotation of the phenyl rings in the aggregate state because of their intermolecular favorable π–π interactions. Accordingly, **C47** already showed a 6-fold increased emission intensity in solution (MeCN) compared to **115** due to short pillar length of **C47**, which hinders rotation. On the other hand, to form emissive aggregates of **C48** and **C49**, H_2_O had to be added to the MeCN solution gradually up to H_2_O:MeCN (9:1). Aggregate formation enhanced the fluorescence emission intensity of **C48** and **C49** at 540 nm up to 25- and 16-fold, respectively.

**Figure 52:**
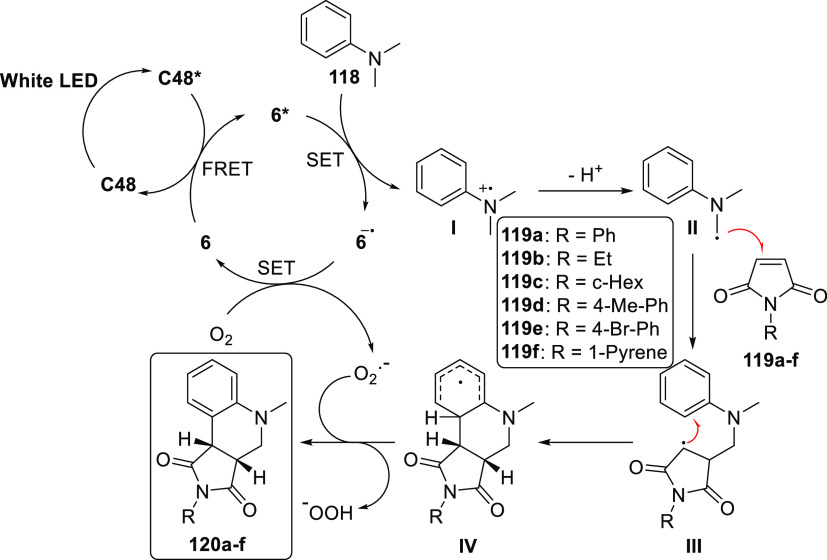
Proposed reaction mechanism for the light-harvesting **C48**:**RhB** complex in the photoinduced oxidative cyclization between *N*,*N*-methyl aniline **118** and maleimides **119a**–**f**.^213^

Rhodamine B (**6**) was then selected as acceptor dye as it shows absorption in the same wavelengths as the emission maxima of **C48** and **C49**. The addition of **6** to either **C48** or **C49** resulted in the unaltered UV–vis spectrum of the cages with the added absorption spectrum of **6**. Therefore, the authors suggest that the components have no interactions in the ground state, and thus the cages do not encapsulate **6**. Fluorescence studies and time-correlated single photon counting techniques (TCSPC) showed that FRET occurred efficiently between **C48** or **C49** and **6** (77% or 58%, respectively). Therefore, catalytic transformations were investigated using LH system **C48:6** (5:1) for a photoinduced radical cascade cyclization between *N*,*N*-dimethylaniline **118** and *N*-substituted maleimides **119a**–**f** using white LEDs at rt for 12 h (Figure 52). The reaction is initiated by the oxidation of the amine by excited **6**, to form radical cation **I**, which loses a proton to form carbon-centered radical **II**. The maleimide reacts with this radical to form a C–C bond (**III**), which then ring closes to make dearomatized **IV**. After sequential oxidation by superoxide from air and deprotonation, this gives products **120a**–**f** in high yield (74–99%). The same reaction conditions in absence of **C48** with **6**, 1 or 5 mol %, resulted in poor conversion (5% or 14%, respectively). Accordingly, the authors conclude that **C48** acts as LH system that collects higher energy photons and performs FRET to **6**, increasing the activity of the photocatalytic transformation.

To summarize, four general strategies have been successfully exploited to promote photochemical transformations inside the cavity of cages. The group of Fujita has performed the pioneering work with **C36a** by using UV light to promote photochemical transformations of various encapsulated substrates. The unique interaction between host and guest has been exploited to excite the emerging CT complexes that give rise to radical reactivity of the guest by the groups of Dasgupta and Sun. Increased interest of the chemical community in photoredox catalysis has stimulated the development of cages that contain photoredox moieties either in the linker or in the node. These hosts can subsequently be used to promote photoredox-type transformations of encapsulated guests, giving rise to unique reactivity. Finally, the group of Su has shown that stereoselectivity can be induced in the cavity of homochiral **C33**, encouraging future research to pursue different kinds of photoredox chemistry inside of the cavities of chiral molecular cages.

## 5 Toward Implementation in Devices

Up to now, light-driven catalysis utilizing coordination cages has been almost exclusively taking place in homogeneous solution or at least in suspension. As mentioned in the introduction, the ultimate goal of artificial photosynthesis is to combine a light-driven oxidation reaction (e.g., water oxidation) with light-driven reduction (e.g., proton reduction) in one material or device. Likewise, for organic synthesis, it would be beneficial to be able to recycle the cage catalysts or use them in a continuous setup.^217^ Therefore, immobilization of cages on a heterogeneous support might be useful, as it would allow easier separation of products from the cage catalysts. In this final part, we summarize strategies for immobilization of cages on (electrode) surfaces. The strategies discussed may be used both for the construction of cage-based DS-PECs, and (more generally) for integration of cage catalysts in flow reactors and for developing catalyst recycling strategies.

**Figure 53:**
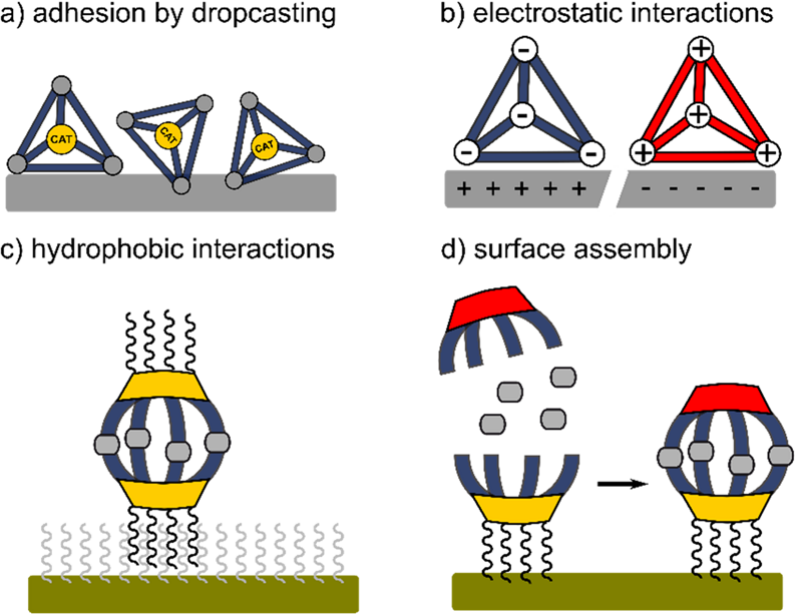
Different strategies for cage immobilization on solid support: (a) drop-casting, (b) electrostatic interactions (e.g., on charged polymers or alumina), (c) hydrophobic interactions with SAM on gold, (d) stepwise assembly on gold surface by first installing an SAM of cavitand then followed by metal-mediated assembly of the cage.

In recent literature, several methods have been developed to immobilize coordination cages on different heterogeneous supports such as gold,^218,219^ alumina,^220^ and polymers.^221^ Depending on the purpose and the nature of the support, different strategies are used as depicted in Figure 53.

The most simple approach of immobilization is to drop-cast a suspension of the cage on a solid material, e.g., an electrode. Similar strategies have been used to drop cast MOFs for electrochemical studies.^222^ Qi et al. have drop-casted **C35** dispersed in 500 μL water/0.5 wt % naphthol aqueous solution/ethanol mixed solvent (V/V/V = 1:2:2) on glassy carbon electrodes in order to perform cyclic voltammetry to investigate electrochemical properties of the cage catalyst.^154^ Although this strategy may be useful for electrochemical analysis, such films are typically unstable and will not remain functional for long-term operation.

Another, more synthetically demanding, strategy is the construction of cages on a surface. Au surfaces can generally be functionalized with small molecules by immobilization of thiols and dithiols in the form of self-assembled monolayers (SAM).^223^ Reinhoudt and co-workers utilized this strategy to construct coordination cages based on various cavitands and Pd(II) or Pt(II) as connecting nodes on Au(111) surfaces.^218,219^ First, Au(111) was prefunctionalized with a SAM of a cavitand containing long alkylchains terminated with thiols by soaking the substrate in a solution of the cavitand (0.1 mM) in EtOH:CHCl_3_ (3:1). In a second step, the cages are constructed via metal mediated self-assembly with metal complex M(dppp)(OTf)_2_ (M = Pd(II) or Pt(II), dppp = diphenylphosphine-propyl) and a second (different) cavitand (Figure 53d). Here, the bidentate capping ligand dppp ensures the formation of discrete cages instead of coordination polymers. Analysis of the cage-functionalized Au surface by atomic force microscopy (AFM) revealed two distinct species in ratio 1:1 of 2 and 4 nm size, respectively. While the latter was attributed to the self-assembled coordination cages, the smaller species most likely is the SAM only. Apparently, only 50% of the pre-immobilized SAM was accessible for the construction of surface cages in this method.

As an alternative strategy, the same group prepared homoleptic, pre-assembled cages from a cavitand containing alkylthiolate chains and M(dppp) (Figure 53c). Making use of hydrophobic interactions of the long alkyl chains, these cages could be immobilized on Au surfaces that were prefunctionalized with a SAM of simple alkylthiolate molecules. This strategy allows the immobilization of preformed cages and can in principle also be applied to heteroleptic cages with one type of ligand decorated with alkyl chains. Dalcanale and co-workers followed a similar strategy to assemble and immobilize cages on Si surfaces.^224,225^

**Figure 54:**
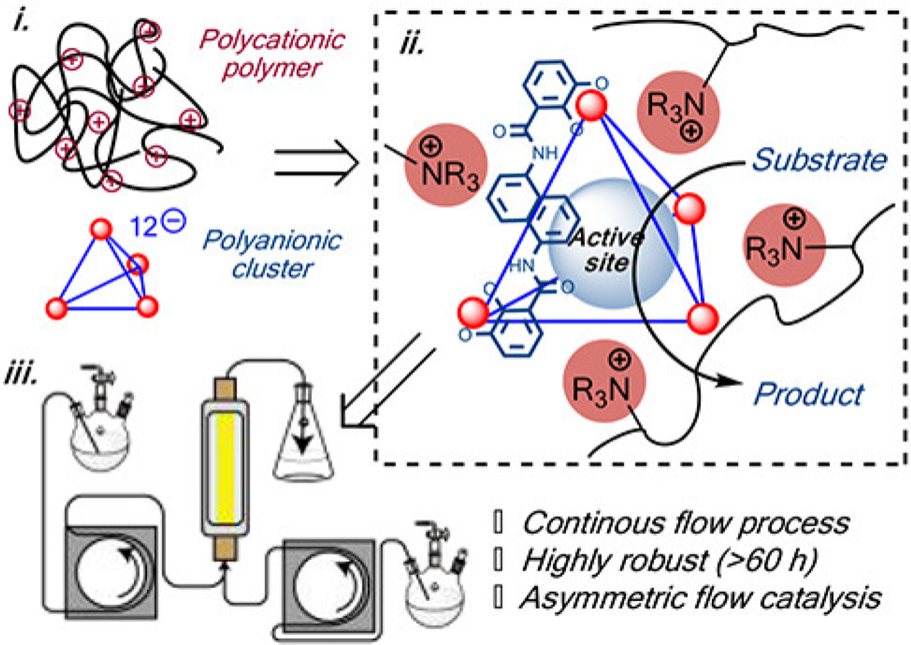
Coordination cage immobilized on polymer via electrostatic interactions used in continuous flow setup for catalysis. Adapted with permission from ref (221). Copyright 2020 American Chemical Society.

A different approach is making use of electrostatic interactions between cage and support (Figure 53b). Raymond and co-workers utilized electrostatic interactions to immobilize anionic cage **C41** on a cross-linked polymer with cationic functionalities (Figure 54).^221^ The cage-functionalized polymer was employed as heterogeneous catalyst for aza-Prins and aza-Cope reactions in a continuous-flow setup. Interestingly, the heterogenized cage catalyst displayed enhanced activity and significantly increased robustness (>60 h) compared to the corresponding homogeneous system in solution.

Nitschke and co-workers developed a general protocol to immobilize cages on acidic or basic alumina. The cavities of the adsorbed cages were still accessible for guest encapsulation. As proof-of-principle, the group demonstrated the separation of Diels–Alder reagents by two immobilized cages.^220^

In summary, the immobilization of coordination cages on various heterogeneous support materials has been reported. Raymond and Nitschke have demonstrated the ability of immobilized cages to act as catalysts under continuous flow conditions, in the case of Raymond even with higher performance than the homogeneous analogues. Despite the high potential, the use of such immobilized cages in catalytic applications is very limited to date. Further research in this direction, including electrochemical applications, is required to bridge the gap to application. For cage catalyst recycling, a wider scope of approaches may be available, for example, the formation of polymers with cages as building blocks. As discussed in section 3.2.3, Wisser and co-workers utilized the polymer strategy to connect Rh spheres with ditopic imidazole ligands to form polymer particles. This approach not only resulted in stabilization of the Rh catalysts but also allowed better recycling of the heterogenized catalyst.

## 6 Conclusion and Outlook

We have reviewed the application of supramolecular coordination cages in light-driven catalysis and have discussed various strategies. By focusing on artificial photosynthesis and photocatalysis for synthesis in separate sections, a broad overview of the opportunities provided by supramolecular coordination cages is presented. The differences in goals between artificial photosynthesis and photo(redox)catalysis implies that the supramolecular cage strategies are rather different. The literature examples in both sections are organized based on the position of the PS within the supramolecular construct.

Supramolecular cages provide an interesting platform for artificial photosynthesis to organize different required components in close proximity to optimize for function. As highlighted in Figure 4, several strategies are possible, which in general work well for homogeneous reactions. The redox potentials of PS and catalyst can be fine-tuned by supramolecular interactions between host and guest, which can also lower the overpotentials for the desired half-reaction. The same interactions also stabilize the catalytic intermediates, which allows for a more efficient process as the time difference of photophysical processes and catalysis can be bridged more easily. For the overall functioning of the assembly within a device, directional electron transfer becomes crucial. Supramolecular cages with encapsulated catalysts that allow directional electron transfer are therefore promising, especially if such a light-absorbing cage can be immobilized on an electrode surface.

In organic photocatalysis, supramolecular cages can be used to induce selectivity, increase the rate, and change the outcome of the photochemical reactions. This is achieved by pre-organization effects (leading to high local concentration) and spatial constraints induced by the supramolecular cavity. Additionally, due to unique host–guest CT interactions, lower energy light may be used, offering multiple advantages. Moreover, a chiral cavity is able to transfer the chirality to the reaction products, which allows for enantioselective photoredox catalysis. However, it is key to understand the ground-state host–guest equilibria and their interactions to achieve the maximum impact from the supramolecular strategy.

Herein, we have shown that supramolecular coordination cages are attractive reaction vessels for photocatalytic methodologies. Although the design and synthesis of new (photoactive) cages is ongoing work and much progress has already been achieved, the use of these novel cages for photocatalysis often still remains underexplored. Moreover, fundamental studies aiming at a more detailed understanding of (photoinduced) electron and energy transfer processes in confined space should provide more insight into the fundamental mechanistic steps. Until now, pump/probe spectroscopic techniques have shown to be very insightful when it comes to characterizing short-lived intermediates and following the electron transfer pathway, thus providing the desired insight. Eventually, both the ground-state properties of the host–guest system as well as the excited-state reactivity need to be well understood to allow efficient excited-state transformations by design in a rational way.

Interestingly, photoredox catalysis and artificial photosynthesis have recently received enormous interest from the chemical community, yet these communities operate rather independently from one another. Although the goals are largely different, there may be some general lessons to learn, and mutual inspiration is likely. Charge directionality, preventing charge recombination and stabilization of instable intermediates are such common goals. Some of these challenges can be met by execution with and/or inside the cavities of supramolecular hosts. This will expand the scope of photochemically active cages and in turn will help to understand and design even more efficient strategies.

Most of the cages presented in this review are homoleptic cages with only one type of ligand. In some cases, ligand mixtures have been used in a statistical mixture. The application of heteroleptic cages in which different ligand building blocks are used, potentially each with distinct positions and functionality, provides a next level of organization. Pioneering work on construction of such heteroleptic structures has been performed by the groups of Stang, Fujita, and Clever, and has been summarized in a recent review.^69^ Finally, the immobilization of supramolecular cages on a heterogeneous support like alumina or polymers may lead to recyclable and long-term stable photocatalysts based on coordination cages. Immobilizing cages on electrode surfaces or semiconductors will allow their implementation on (photo)-electrochemical devices. These steps are without doubt required to bring this field a step closer to application.

## References

[ref1] Ravelli D.; Dondi D.; Fagnoni M.; Albini A. Photocatalysis. A Multi-Faceted Concept for Green Chemistry. Chem. Soc. Rev. 2009, 38, 1999–2011. 10.1039/b714786b. 19551179

[ref2] Candish L.; Collins K. D.; Cook G. C.; Douglas J. J.; Gómez-Suárez A.; Jolit A.; Keess S. Photocatalysis in the Life Science Industry. Chem. Rev. 2022, 122, 2907–2980. 10.1021/acs.chemrev.1c00416. 34558888

[ref3] Pitre S. P.; Overman L. E. Strategic Use of Visible-Light Photoredox Catalysis in Natural Product Synthesis. Chem. Rev. 2022, 122, 1717–1751. 10.1021/acs.chemrev.1c00247. 34232019

[ref4] Lechner V. M.; Nappi M.; Deneny P. J.; Folliet S.; Chu J. C. K.; Gaunt M. J. Visible-Light-Mediated Modification and Manipulation of Biomacromolecules. Chem. Rev. 2022, 122, 1752–1829. 10.1021/acs.chemrev.1c00357. 34546740

[ref5] Berardi S.; Drouet S.; Francàs L.; Gimbert-Suriñach C.; Guttentag M.; Richmond C.; Stoll T.; Llobet A. Molecular Artificial Photosynthesis. Chem. Soc. Rev. 2014, 43, 7501–7519. 10.1039/C3CS60405E. 24473472

[ref6] Keijer T.; Bouwens T.; Hessels J.; Reek J. N. H. Supramolecular Strategies in Artificial Photosynthesis. Chem. Sci. 2021, 12, 50–70. 10.1039/D0SC03715J. PMC8179670 34168739

[ref7] McEvoy J. P.; Brudvig G. W. Water-Splitting Chemistry of Photosystem II. Chem. Rev. 2006, 106, 4455–4483. 10.1021/cr0204294. 17091926

[ref8] Berg J. M.; Tymoczko J. L.; Gatto G. J.; Stryer L. Biochemistry, 8th ed.; W.H. Freeman & Company: New York, 2015; pp 215–245.

[ref9] Ward M. D.; Raithby P. R. Functional Behaviour from Controlled Self-Assembly: Challenges and Prospects. Chem. Soc. Rev. 2013, 42, 1619–1636. 10.1039/C2CS35123D. 22797247

[ref10] Conn M. M.; Rebek J. Self-Assembling Capsules. Chem. Rev. 1997, 97, 1647–1668. 10.1021/cr9603800. 11851461

[ref11] Cook T. R.; Stang P. J. Recent Developments in the Preparation and Chemistry of Metallacycles and Metallacages via Coordination. Chem. Rev. 2015, 115, 7001–7045. 10.1021/cr5005666. 25813093

[ref12] Pullen S.; Clever G. H. Mixed-Ligand Metal-Organic Frameworks and Heteroleptic Coordination Cages as Multifunctional Scaffolds - A Comparison. Acc. Chem. Res. 2018, 51, 3052–3064. 10.1021/acs.accounts.8b00415. 30379523 PMC6437652

[ref13] Sun Y.; Chen C.; Liu J.; Stang P. J. Recent Developments in the Construction and Applications of Platinum-Based Metallacycles and Metallacages via Coordination. Chem. Soc. Rev. 2020, 49, 3889–3919. 10.1039/D0CS00038H. 32412574 PMC7846457

[ref14] Pan M.; Wu K.; Zhang J. H.; Su C. Y. Chiral Metal-Organic Cages/Containers (MOCs): From Structural and Stereochemical Design to Applications. Coord. Chem. Rev. 2019, 378, 333–349. 10.1016/j.ccr.2017.10.031.

[ref15] Chen L. J.; Yang H. B.; Shionoya M. Chiral Metallosupramolecular Architectures. Chem. Soc. Rev. 2017, 46, 2555–2576. 10.1039/C7CS00173H. 28452389

[ref16] Würthner F.; You C. C.; Saha-Möller C. R. Metallosupramolecular Squares: From Structure to Function. Chem. Soc. Rev. 2004, 33, 133–146. 10.1039/B300512G. 15026818

[ref17] Zhang Y. Y.; Gao W. X.; Lin L.; Jin G. X. Recent Advances in the Construction and Applications of Heterometallic Macrocycles and Cages. Coord. Chem. Rev. 2017, 344, 323–344. 10.1016/j.ccr.2016.09.010.

[ref18] Caulder D. L.; Raymond K. N. Supermolecules by Design. Acc. Chem. Res. 1999, 32, 975–982. 10.1021/ar970224v.

[ref19] Chen L.; Chen Q.; Wu M.; Jiang F.; Hong M. Controllable Coordination-Driven Self-Assembly: From Discrete Metallocages to Infinite Cage-Based Frameworks. Acc. Chem. Res. 2015, 48, 201–210. 10.1021/ar5003076. 25517043

[ref20] Smulders M. M. J.; Riddell I. A.; Browne C.; Nitschke J. R. Building on Architectural Principles for Three-Dimensional Metallosupramolecular Construction. Chem. Soc. Rev. 2013, 42, 1728–1754. 10.1039/C2CS35254K. 23032789

[ref21] Fujita M.; Tominaga M.; Hori A.; Therrien B. Coordination Assemblies from a Pd(II)-Cornered Square Complex. Acc. Chem. Res. 2005, 38, 369–378. 10.1021/ar040153h. 15835883

[ref22] Otte M. Size-Selective Molecular Flasks. ACS Catal. 2016, 6, 6491–6510. 10.1021/acscatal.6b01776.

[ref23] Ward M. D.; Hunter C. A.; Williams N. H. Coordination Cages Based on Bis(Pyrazolylpyridine) Ligands: Structures, Dynamic Behavior, Guest Binding, and Catalysis. Acc. Chem. Res. 2018, 51, 2073–2082. 10.1021/acs.accounts.8b00261. 30085644

[ref24] Rizzuto F. J.; von Krbek L. K. S.; Nitschke J. R. Strategies for Binding Multiple Guests in Metal-Organic Cages. Nat. Rev. Chem. 2019, 3, 204–222. 10.1038/s41570-019-0085-3.

[ref25] Wiester M. J.; Ulmann P. A.; Mirkin C. A. Enzyme Mimics Based Upon Supramolecular Coordination Chemistry. Angew. Chem., Int. Ed. 2011, 50, 114–137. 10.1002/anie.201000380. 20922725

[ref26] Leenders S. H. A. M.; Gramage-Doria R.; de Bruin B.; Reek J. N. H. Transition Metal Catalysis in Confined Spaces. Chem. Soc. Rev. 2015, 44, 433–448. 10.1039/C4CS00192C. 25340992

[ref27] Brown C. J.; Toste F. D.; Bergman R. G.; Raymond K. N. Supramolecular Catalysis in Metal-Ligand Cluster Hosts. Chem. Rev. 2015, 115, 3012–3035. 10.1021/cr4001226. 25898212

[ref28] Morimoto M.; Bierschenk S. M.; Xia K. T.; Bergman R. G.; Raymond K. N.; Toste F. D. Advances in Supramolecular Host-Mediated Reactivity. Nat. Catal. 2020, 3, 969–984. 10.1038/s41929-020-00528-3.

[ref29] Jongkind L. J.; Caumes X.; Hartendorp A. P. T.; Reek J. N. H. Ligand Template Strategies for Catalyst Encapsulation. Acc. Chem. Res. 2018, 51, 2115–2128. 10.1021/acs.accounts.8b00345. 30137959 PMC6148444

[ref30] Koblenz T. S.; Wassenaar J.; Reek J. N. H. Reactivity within a Confined Self-Assembled Nanospace. Chem. Soc. Rev. 2008, 37, 247–262. 10.1039/B614961H. 18197342

[ref31] Mouarrawis V.; Plessius R.; van der Vlugt J. I.; Reek J. N. H. Confinement Effects in Catalysis Using Well-Defined Materials and Cages. Front. Chem. 2018, 6, 623 10.3389/fchem.2018.00623. 30622940 PMC6308152

[ref32] Jans A. C. H.; Caumes X.; Reek J. N. H. Gold Catalysis in (Supra)Molecular Cages to Control Reactivity and Selectivity. ChemCatChem. 2019, 11, 287–297. 10.1002/cctc.201801399. 30854145 PMC6391950

[ref33] Grommet A. B.; Feller M.; Klajn R. Chemical Reactivity under Nanoconfinement. Nat. Nanotechnol. 2020, 15, 256–271. 10.1038/s41565-020-0652-2. 32303705

[ref34] Huang B.; Mao L.; Shi X.; Yang H. B. Recent Advances and Perspectives on Supramolecular Radical Cages. Chem. Sci. 2021, 12, 13648–13663. 10.1039/D1SC01618K. 34760150 PMC8549795

[ref35] Jin Y.; Zhang Q.; Zhang Y.; Duan C. Electron Transfer in the Confined Environments of Metal-Organic Coordination Supramolecular Systems. Chem. Soc. Rev. 2020, 49, 5561–5600. 10.1039/C9CS00917E. 32643720

[ref36] Regeni I.; Chen B.; Frank M.; Baksi A.; Holstein J. J.; Clever G. H. Coal-Tar Dye-Based Coordination Cages and Helicates. Angew. Chem., Int. Ed. 2021, 60, 5673–5678. 10.1002/anie.202015246. PMC7986857 33245206

[ref37] Jing X.; He C.; Zhao L.; Duan C. Photochemical Properties of Host-Guest Supramolecular Systems with Structurally Confined Metal-Organic Capsules. Acc. Chem. Res. 2019, 52, 100–109. 10.1021/acs.accounts.8b00463. 30586276

[ref38] Jiao Y.; Zuo Y.; Yang H.; Gao X.; Duan C. Photoresponse within Dye-Incorporated Metal-Organic Architectures. Coord. Chem. Rev. 2021, 430, 213648 10.1016/j.ccr.2020.213648.

[ref39] Rota Martir D.; Zysman-Colman E. Photoactive Supramolecular Cages Incorporating Ru(II) and Ir(III) Metal Complexes. Chem. Commun. 2019, 55, 139–158. 10.1039/C8CC08327D. 30511080

[ref40] Rota Martir D.; Zysman-Colman E. Supramolecular Iridium(III) Assemblies. Coord. Chem. Rev. 2018, 364, 86–117. 10.1016/j.ccr.2018.03.016.

[ref41] Großkopf J.; Kratz T.; Rigotti T.; Bach T. Enantioselective Photochemical Reactions Enabled by Triplet Energy Transfer. Chem. Rev. 2022, 122, 1626–1653. 10.1021/acs.chemrev.1c00272. 34227803

[ref42] Santoro A.; Bella G.; Cancelliere A. M.; Serroni S.; Lazzaro G.; Campagna S. Photoinduced Electron Transfer in Organized Assemblies-Case Studies. Molecules 2022, 27, 2713 10.3390/molecules27092713. 35566062 PMC9102318

[ref43] Balzani V.; Credi A.; Venturi M. Photoprocesses. Curr. Opin. Chem. Biol. 1997, 1, 506–513. 10.1016/S1367-5931(97)80045-2. 9667884

[ref44] Berg J. M.; Tymoczko J. L.; Gatto G. J.; Stryer L. Biochemistry, 8th ed.; W.H. Freeman & Company: New York, 2015; pp 565–588.

[ref45] Cha H. G.; Choi K. S. Combined Biomass Valorization and Hydrogen Production in a Photoelectrochemical Cell. Nat. Chem. 2015, 7, 328–333. 10.1038/nchem.2194. 25803471

[ref46] Collinson S. R.; Thielemans W. The Catalytic Oxidation of Biomass to New Materials Focusing on Starch, Cellulose and Lignin. Coord. Chem. Rev. 2010, 254, 1854–1870. 10.1016/j.ccr.2010.04.007.

[ref47] Weber R. S.; Ramasamy K. K. Electrochemical Oxidation of Lignin and Waste Plastic. ACS Omega 2020, 5, 27735–27740. 10.1021/acsomega.0c03989. 33163755 PMC7643066

[ref48] Bruggeman D. F.; Laporte A. A. H.; Detz R. J.; Mathew S.; Reek J. N. H. Aqueous Biphasic Dye-Sensitized Photosynthesis Cells for TEMPO-Based Oxidation of Glycerol. Angew. Chem., Int. Ed. 2022, 61, e202200175 10.1002/anie.202200175. PMC9401026 35266261

[ref49] Pellegrin Y.; Odobel F. Sacrificial Electron Donor Reagents for Solar Fuel Production. C. R. Chim. 2017, 20, 283–295. 10.1016/j.crci.2015.11.026.

[ref50] Pinaud B. A.; Benck J. D.; Seitz L. C.; Forman A. J.; Chen Z.; Deutsch T. G.; James B. D.; Baum K. N.; Baum G. N.; Ardo S.; et al. Technical and Economic Feasibility of Centralized Facilities for Solar Hydrogen Production via Photocatalysis and Photoelectrochemistry. Energy Environ. Sci. 2013, 6, 1983–2002. 10.1039/c3ee40831k.

[ref51] Fujishima A.; Honda K. Electrochemical Photolysis of Water at a Semiconductor Electrode. Nature 1972, 238, 37–38. 10.1038/238037a0. 12635268

[ref52] Grätzel M. Photoelectrochemical Cells. Nature 2001, 414, 338–344. 10.1038/35104607. 11713540

[ref53] Holmberg-Douglas N.; Nicewicz D. A. Photoredox-Catalyzed C-H Functionalization Reactions. Chem. Rev. 2022, 122, 1925–2016. 10.1021/acs.chemrev.1c00311. 34585909 PMC8939264

[ref54] Chan A. Y.; Perry I. B.; Bissonnette N. B.; Buksh B. F.; Edwards G. A.; Frye L. I.; Garry O. L.; Lavagnino M. N.; Li B. X.; Liang Y.; et al. Metallaphotoredox: The Merger of Photoredox and Transition Metal Catalysis. Chem. Rev. 2022, 122, 1485–1542. 10.1021/acs.chemrev.1c00383. 34793128 PMC12232520

[ref55] Prier C. K.; Rankic D. A.; MacMillan D. W. C. Visible Light Photoredox Catalysis with Transition Metal Complexes: Applications in Organic Synthesis. Chem. Rev. 2013, 113, 5322–5363. 10.1021/cr300503r. 23509883 PMC4028850

[ref56] Narayanam J. M. R.; Stephenson C. R. J. Visible Light Photoredox Catalysis: Applications in Organic Synthesis. Chem. Soc. Rev. 2011, 40, 102–113. 10.1039/B913880N. 20532341

[ref57] Shaw M. H.; Twilton J.; MacMillan D. W. C. Photoredox Catalysis in Organic Chemistry. J. Org. Chem. 2016, 81, 6898–6926. 10.1021/acs.joc.6b01449. 27477076 PMC4994065

[ref58] Romero N. A.; Nicewicz D. A. Organic Photoredox Catalysis. Chem. Rev. 2016, 116, 10075–10166. 10.1021/acs.chemrev.6b00057. 27285582

[ref59] Strieth-Kalthoff F.; James M. J.; Teders M.; Pitzer L.; Glorius F. Energy Transfer Catalysis Mediated by Visible Light: Principles, Applications, Directions. Chem. Soc. Rev. 2018, 47, 7190–7202. 10.1039/C8CS00054A. 30088504

[ref60] Zhao M.; Ou S.; Wu C. de. Porous Metal-Organic Frameworks for Heterogeneous Biomimetic Catalysis. Acc. Chem. Res. 2014, 47, 1199–1207. 10.1021/ar400265x. 24499017

[ref61] Kluwer A. M.; Kapre R.; Hartl F.; Lutz M.; Spek A. L.; Brouwer A. M.; van Leeuwen P. W. N. M.; Reek J. N. H. Self-Assembled Biomimetic [2Fe2S]-Hydrogenase-Based Photocatalyst for Molecular Hydrogen Evolution. Proc. Natl. Acad. Sci. U.S.A. 2009, 106, 10460–10465. 10.1073/pnas.0809666106. 19164541 PMC2705529

[ref62] Nguyen Q. N. N.; Xia K. T.; Zhang Y.; Chen N.; Morimoto M.; Pei X.; Ha Y.; Guo J.; Yang W.; Wang L. P.; et al. Source of Rate Acceleration for Carbocation Cyclization in Biomimetic Supramolecular Cages. J. Am. Chem. Soc. 2022, 144, 11413–11424. 10.1021/jacs.2c04179. 35699585

[ref63] Zhao L.; Jing X.; Li X.; Guo X.; Zeng L.; He C.; Duan C. Catalytic Properties of Chemical Transformation within the Confined Pockets of Werner-Type Capsules. Coord. Chem. Rev. 2019, 378, 151–187. 10.1016/j.ccr.2017.11.005.

[ref64] Bowman-James K. Alfred Werner Revisited: The Coordination Chemistry of Anions. Acc. Chem. Res. 2005, 38, 671–678. 10.1021/ar040071t. 16104690

[ref65] Feng L.; Wang K. Y.; Day G. S.; Zhou H. C. The Chemistry of Multi-Component and Hierarchical Framework Compounds. Chem. Soc. Rev. 2019, 48, 4823–4853. 10.1039/C9CS00250B. 31359020

[ref66] Saha S.; Regeni I.; Clever G. H. Structure Relationships between Bis-Monodentate Ligands and Coordination Driven Self-Assemblies. Coord. Chem. Rev. 2018, 374, 1–14. 10.1016/j.ccr.2018.06.010.

[ref67] Decker G. E.; Lorzing G. R.; Deegan M. M.; Bloch E. D. MOF-Mimetic Molecules: Carboxylate-Based Supramolecular Complexes as Molecular Metal-Organic Framework Analogues. J. Mater. Chem. A 2020, 8, 4217–4229. 10.1039/C9TA12497G.

[ref68] Chakrabarty R.; Mukherjee P. S.; Stang P. J. Supramolecular Coordination: Self-Assembly of Finite Two- and Three-Dimensional Ensembles. Chem. Rev. 2011, 111, 6810–6918. 10.1021/cr200077m. 21863792 PMC3212633

[ref69] Pullen S.; Tessarolo J.; Clever G. H. Increasing Structural and Functional Complexity in Self-Assembled Coordination Cages. Chem. Sci. 2021, 12, 7269–7293. 10.1039/D1SC01226F. 34163819 PMC8171321

[ref70] Piskorz T. K.; Martí-Centelles V.; Young T. A.; Lusby P. J.; Duarte F. Computational Modeling of Supramolecular Metallo-Organic Cages-Challenges and Opportunities. ACS Catal. 2022, 5806–5826. 10.1021/acscatal.2c00837. 35633896 PMC9127791

[ref71] Jelfs K. E. Computational Modeling to Assist in the Discovery of Supramolecular Materials. Ann. N.Y. Acad. Sci. 2021, 1–14. 10.1039/9781839163319-00001. PMC10091946 36251351

[ref72] Pluth M. D.; Raymond K. N. Reversible Guest Exchange Mechanisms in Supramolecular Host-Guest Assemblies. Chem. Soc. Rev. 2007, 36, 161–171. 10.1039/B603168B. 17264920

[ref73] Galan A.; Ballester P. Stabilization of Reactive Species by Supramolecular Encapsulation. Chem. Soc. Rev. 2016, 45, 1720–1737. 10.1039/C5CS00861A. 26797259

[ref74] Ye S.; Ding C.; Liu M.; Wang A.; Huang Q.; Li C. Water Oxidation Catalysts for Artificial Photosynthesis. Adv. Mater. 2019, 31, 1902069 10.1002/adma.201902069. 31495962

[ref75] Matheu R.; Garrido-Barros P.; Gil-Sepulcre M.; Ertem M. Z.; Sala X.; Gimbert-Suriñach C.; Llobet A. The Development of Molecular Water Oxidation Catalysts. Nat. Rev. Chem. 2019, 3, 331–341. 10.1038/s41570-019-0096-0.

[ref76] Duan L.; Fischer A.; Xu Y.; Sun L. Isolated Seven-Coordinate Ru(IV) Dimer Complex with [HOHOH]^−^ Bridging Ligand as an Intermediate for Catalytic Water Oxidation. J. Am. Chem. Soc. 2009, 131, 10397–10399. 10.1021/ja9034686. 19601625

[ref77] Duan L.; Bozoglian F.; Mandal S.; Stewart B.; Privalov T.; Llobet A.; Sun L. A Molecular Ruthenium Catalyst with Water-Oxidation Activity Comparable to That of Photosystem II. Nat. Chem. 2012, 4, 418–423. 10.1038/nchem.1301. 22522263

[ref78] Duan L.; Wang L.; Li F.; Li F.; Sun L. Highly Efficient Bioinspired Molecular Ru Water Oxidation Catalysts with Negatively Charged Backbone Ligands. Acc. Chem. Res. 2015, 48, 2084–2096. 10.1021/acs.accounts.5b00149. 26131964

[ref79] Shaffer D. W.; Xie Y.; Concepcion J. J. O-O Bond Formation in Ruthenium-Catalyzed Water Oxidation: Single-Site Nucleophilic Attack vs. O-O Radical Coupling. Chem. Soc. Rev. 2017, 46, 6170–6193. 10.1039/C7CS00542C. 28861558

[ref80] Tong L.; Duan L.; Xu Y.; Privalov T.; Sun L. Structural Modifications of Mononuclear Ruthenium Complexes: A Combined Experimental and Theoretical Study on the Kinetics of Ruthenium-Catalyzed Water Oxidation. Angew. Chem., Int. Ed. 2011, 50, 445–449. 10.1002/anie.201005141. 21154545

[ref81] Kärkäs M. D.; Verho O.; Johnston E. v.; Åkermark B. Artificial Photosynthesis: Molecular Systems for Catalytic Water Oxidation. Chem. Rev. 2014, 114, 11863–12001. 10.1021/cr400572f. 25354019

[ref82] Hessels J.; Detz R. J.; Koper M. T. M.; Reek J. N. H. Rational Design Rules for Molecular Water Oxidation Catalysts Based on Scaling Relationships. Chem.—Eur. J. 2017, 23, 16413–16418. 10.1002/chem.201702850. 28836700

[ref83] Li F.; Zhang B.; Li X.; Jiang Y.; Chen L.; Li Y.; Sun L. Highly Efficient Oxidation of Water by a Molecular Catalyst Immobilized on Carbon Nanotubes. Angew. Chem., Int. Ed. 2011, 50, 12276–12279. 10.1002/anie.201105044. 22028099

[ref84] Li B.; Li F.; Bai S.; Wang Z.; Sun L.; Yang Q.; Li C. Oxygen Evolution from Water Oxidation on Molecular Catalysts Confined in the Nanocages of Mesoporous Silicas. Energy Environ. Sci. 2012, 5, 8229–8233. 10.1039/c2ee22059h.

[ref85] Bhunia A.; Johnson B. A.; Czapla-Masztafiak J.; Sá J.; Ott S. Formal Water Oxidation Turnover Frequencies from MIL-101(Cr) Anchored Ru(bda) Depend on Oxidant Concentration. Chem. Commun. 2018, 54, 7770–7773. 10.1039/C8CC02300J. PMC6040278 29926035

[ref86] Fang T.; Fu L. Z.; Zhou L. L.; Zhan S. Z.; Chen S. Electrochemical-Driven Water Reduction Catalyzed by a Water Soluble Cobalt(III) Complex with Schiff Base Ligand. Electrochim. Acta 2015, 178, 368–373. 10.1016/j.electacta.2015.07.180.

[ref87] Rai S.; Majee K.; Raj M.; Pahari A.; Patel J.; Padhi S. K. Electrocatalytic Proton and Water Reduction by a Co(III) Polypyridyl Complex. Polyhedron 2019, 159, 127–134. 10.1016/j.poly.2018.11.053.

[ref88] Stoll T.; Castillo C. E.; Kayanuma M.; Sandroni M.; Daniel C.; Odobel F.; Fortage J.; Collomb M. N. Photo-Induced Redox Catalysis for Proton Reduction to Hydrogen with Homogeneous Molecular Systems Using Rhodium-Based Catalysts. Coord. Chem. Rev. 2015, 304–305, 20–37. 10.1016/j.ccr.2015.02.002.

[ref89] Zhang P.; Wang M.; Na Y.; Li X.; Jiang Y.; Sun L. Homogeneous Photocatalytic Production of Hydrogen from Water by a Bioinspired [Fe_2_S_2_] Catalyst with High Turnover Numbers. Dalton Trans. 2010, 39, 1204–1206. 10.1039/B923159P. 20104346

[ref90] Teets T. S.; Nocera D. G. Photocatalytic Hydrogen Production. Chem. Commun. 2011, 47, 9268–9274. 10.1039/c1cc12390d. 21647489

[ref91] Mazzeo A.; Santalla S.; Gaviglio C.; Doctorovich F.; Pellegrino J. Recent Progress in Homogeneous Light-Driven Hydrogen Evolution Using First-Row Transition Metal Catalysts. Inorg. Chim. Acta 2021, 517, 119950 10.1016/j.ica.2020.119950.

[ref92] Li Y.; Rauchfuss T. B. Synthesis of Diiron(I) Dithiolato Carbonyl Complexes. Chem. Rev. 2016, 116, 7043–7077. 10.1021/acs.chemrev.5b00669. 27258046 PMC4933964

[ref93] Schilter D.; Camara J. M.; Huynh M. T.; Hammes-Schiffer S.; Rauchfuss T. B. Hydrogenase Enzymes and Their Synthetic Models: The Role of Metal Hydrides. Chem. Rev. 2016, 116, 8693–8749. 10.1021/acs.chemrev.6b00180. 27353631 PMC5026416

[ref94] Gloaguen F.; Rauchfuss T. B. Small Molecule Mimics of Hydrogenases: Hydrides and Redox. Chem. Soc. Rev. 2009, 38, 100–108. 10.1039/B801796B. 19088969 PMC3462221

[ref95] Montzka S. A.; Dlugokencky E. J.; Butler J. H. Non-CO_2_ Greenhouse Gases and Climate Change. Nature 2011, 476, 43–50. 10.1038/nature10322. 21814274

[ref96] Kalamaras E.; Maroto-Valer M. M.; Andresen J. M.; Wang H.; Xuan J. Thermodynamic Analysis of the Efficiency of Photoelectrochemical CO2 Reduction to Ethanol. Energy Procedia 2019, 158, 767–772. 10.1016/j.egypro.2019.01.204.

[ref97] Tamaki Y.; Morimoto T.; Koike K.; Ishitani O. Photocatalytic CO2 Reduction with High Turnover Frequency and Selectivity of Formic Acid Formation Using Ru(II) Multinuclear Complexes. Proc. Natl. Acad. Sci. U.S.A. 2012, 109, 15673–15678. 10.1073/pnas.1118336109. 22908243 PMC3465408

[ref98] Takeda H.; Koike K.; Inoue H.; Ishitani O. Development of an Efficient Photocatalytic System for CO2 Reduction Using Rhenium(I) Complexes Based on Mechanistic Studies. J. Am. Chem. Soc. 2008, 130, 2023–2031. 10.1021/ja077752e. 18205359

[ref99] Gholamkhass B.; Mametsuka H.; Koike K.; Tanabe T.; Furue M.; Ishitani O. Architecture of Supramolecular Metal Complexes for Photocatalytic CO_2_ Reduction: Ruthenium-Rhenium Bi- and Tetranuclear Complexes. Inorg. Chem. 2005, 44, 2326–2336. 10.1021/ic048779r. 15792468

[ref100] Lee H. S.; Jee S.; Kim R.; Bui H. T.; Kim B.; Kim J. K.; Park K. S.; Kim W.; et al. A Highly Active, Robust Photocatalyst Heterogenized in Discrete Cages of Metal-Organic Polyhedra for CO_2_ Reduction. Energy Environ. Sci. 2020, 13, 519–526. 10.1039/C9EE02619C.

[ref101] Appel A. M.; Bercaw J. E.; Bocarsly A. B.; Dobbek H.; Dubois D. L.; Dupuis M.; Ferry J. G.; Fujita E.; Hille R.; Kenis P. J. A.; et al. Frontiers, Opportunities, and Challenges in Biochemical and Chemical Catalysis of CO_2_ Fixation. Chem. Rev. 2013, 113, 6621–6658. 10.1021/cr300463y. 23767781 PMC3895110

[ref102] Ma G.; Yan H.; Zong X.; Ma B.; Jiang H.; Wen F.; Li C. Photocatalytic Splitting of H_2_S to Produce Hydrogen by Gas-Solid Phase Reaction. Chin. J. Catal. 2008, 29, 313–315. 10.1016/S1872-2067(08)60029-7.

[ref103] Oladipo H.; Yusuf A.; Al Jitan S.; Palmisano G. Overview and Challenges of the Photolytic and Photocatalytic Splitting of H_2_S. Catal. 2021, 380, 125–137. 10.1016/j.cattod.2021.03.021.

[ref104] Wang H. Hydrogen Production from a Chemical Cycle of H_2_S Splitting. Int. J. Hydrog. Energy 2007, 32, 3907–3914. 10.1016/j.ijhydene.2007.05.030.

[ref105] Babauta J. T.; Atci E.; Ha P. T.; Lindemann S. R.; Ewing T.; Call D. R.; Fredrickson J. K.; Beyenal H. Localized Electron Transfer Rates and Microelectrode-Based Enrichment of Microbial Communities within a Phototrophic Microbial Mat. Front. Microbiol. 2014, 5, 11 10.3389/fmicb.2014.00011. 24478768 PMC3902354

[ref106] Schulze M.; Kunz V.; Frischmann P. D.; Würthner F. A Supramolecular Ruthenium Macrocycle with High Catalytic Activity for Water Oxidation That Mechanistically Mimics Photosystem II. Nat. Chem. 2016, 8, 576–583. 10.1038/nchem.2503. 27219702

[ref107] Kunz V.; Schulze M.; Schmidt D.; Wurthner F. Trinuclear Ruthenium Macrocycles: Toward Supramolecular Water Oxidation Catalysis in Pure Water. ACS Energy Lett. 2017, 2, 288–293. 10.1021/acsenergylett.6b00560.

[ref108] Meza-Chincha A. L.; Schindler D.; Natali M.; Würthner F. Effects of Photosensitizers and Reaction Media on Light-Driven Water Oxidation with Trinuclear Ruthenium Macrocycles. ChemPhotoChem. 2021, 5, 173–183. 10.1002/cptc.202000133.

[ref109] Xu Y.; Duan L.; Tong L.; Åkermark B.; Sun L. Visible Light-Driven Water Oxidation Catalyzed by a Highly Efficient Dinuclear Ruthenium Complex. Chem. Commun. 2010, 46, 6506–6508. 10.1039/c0cc01250e. 20697637

[ref110] Berardi S.; Francàs L.; Neudeck S.; Maji S.; Benet-Buchholz J.; Meyer F.; Llobet A. Efficient Light-Driven Water Oxidation Catalysis by Dinuclear Ruthenium Complexes. ChemSusChem 2015, 8, 3688–3696. 10.1002/cssc.201500798. 26423045

[ref111] Wang L.; Duan L.; Tong L.; Sun L. Visible Light-Driven Water Oxidation Catalyzed by Mononuclear Ruthenium Complexes. J. Catal. 2013, 306, 129–132. 10.1016/j.jcat.2013.06.023.

[ref112] Noll N.; Würthner F. A Calix[4]Arene-Based Cyclic Dinuclear Ruthenium Complex for Light-Driven Catalytic Water Oxidation. Chem.—Eur. J. 2021, 27, 444–450. 10.1002/chem.202004486. 33241573 PMC7839772

[ref113] Schindler D.; Meza-Chincha A. L.; Roth M.; Würthner F. Structure-Activity Relationship for Di- up to Tetranuclear Macrocyclic Ruthenium Catalysts in Homogeneous Water Oxidation. Chem.—Eur. J. 2021, 27, 16938–16946. 10.1002/chem.202100549. 33909302 PMC9290496

[ref114] Liang L.; Luo D.; Zuo T.; Zhou X. P.; Li D. Control over the Synthesis of Homovalent and Mixed-Valence Cubic Cobalt-Imidazolate Cages. Chem. Commun. 2019, 55, 5103–5106. 10.1039/C9CC02083G. 30968892

[ref115] Luo D.; Zhou X. P.; Li D. Beyond Molecules: Mesoporous Supramolecular Frameworks Self-Assembled from Coordination Cages and Inorganic Anions. Angew. Chem., Int. Ed. 2015, 54, 6190–6195. 10.1002/anie.201501081. 25850862

[ref116] Luo D.; Wang X. Z.; Yang C.; Zhou X. P.; Li D. Self-Assembly of Chiral Metal-Organic Tetartoid. J. Am. Chem. Soc. 2018, 140, 118–121. 10.1021/jacs.7b11285. 29235858

[ref117] Chen Z. Y.; Long Z. H.; Wang X. Z.; Zhou J. Y.; Wang X. S.; Zhou X. P.; Li D. Cobalt-Based Metal-Organic Cages for Visible-Light-Driven Water Oxidation. Inorg. Chem. 2021, 60, 10380–10386. 10.1021/acs.inorgchem.1c00907. 34171190

[ref118] Liu G.; Ju Z.; Yuan D.; Hong M. In Situ Construction of a Coordination Zirconocene Tetrahedron. Inorg. Chem. 2013, 52, 13815–13817. 10.1021/ic402428m. 24279331

[ref119] Liu G.; di Yuan Y.; Wang J.; Cheng Y.; Peh S. B.; Wang Y.; Qian Y.; Dong J.; Yuan D.; Zhao D. Process-Tracing Study on the Postassembly Modification of Highly Stable Zirconium Metal-Organic Cages. J. Am. Chem. Soc. 2018, 140, 6231–6234. 10.1021/jacs.8b03517. 29723472

[ref120] Liu G.; Zeller M.; Su K.; Pang J.; Ju Z.; Yuan D.; Hong M. Controlled Orthogonal Self-Assembly of Heterometal-Decorated Coordination Cages. Chem.—Eur. J. 2016, 22, 17345–17350. 10.1002/chem.201604264. 27778381

[ref121] El-Sayed E. S. M.; Yuan Y. D.; Zhao D.; Yuan D. Zirconium Metal-Organic Cages: Synthesis and Applications. Acc. Chem. Res. 2022, 55, 1546–1560. 10.1021/acs.accounts.1c00654. 35579616

[ref122] Ji C.; Wang W.; El-Sayed E. S. M.; Liu G.; Si Y.; Su K.; Ju Z.; Wu F.; Yuan D. A High-Efficiency Dye-Sensitized Pt(II) Decorated Metal-Organic Cage for Visible-Light-Driven Hydrogen Production. Appl. Catal., B 2021, 285, 119782 10.1016/j.apcatb.2020.119782.

[ref123] Drake T.; Ji P.; Lin W. Site Isolation in Metal-Organic Frameworks Enables Novel Transition Metal Catalysis. Acc. Chem. Res. 2018, 51, 2129–2138. 10.1021/acs.accounts.8b00297. 30129753

[ref124] Deshmukh M. S.; Mane V. S.; Kumbhar A. S.; Boomishankar R. Light-Driven Hydrogen Evolution from Water by a Tripodal Silane Based CoII_6_L^1^_8_ Octahedral Cage. Inorg. Chem. 2017, 56, 13286–13292. 10.1021/acs.inorgchem.7b02074. 29043789

[ref125] Gagliardi C. J.; Westlake B. C.; Kent C. A.; Paul J. J.; Papanikolas J. M.; Meyer T. J. Integrating Proton Coupled Electron Transfer (PCET) and Excited States. Coord. Chem. Rev. 2010, 254, 2459–2471. 10.1016/j.ccr.2010.03.001.

[ref126] Sravan Kumar K.; Mane V. S.; Yadav A.; Kumbhar A. S.; Boomishankar R. Photochemical Hydrogen Evolution from Water by a 1D-Network of Octahedral Ni_6_L_8_ Cages. Chem. Commun. 2019, 55, 13156–13159. 10.1039/C9CC06286F. 31617513

[ref127] Meng W.; Breiner B.; Rissanen K.; Thoburn J. D.; Clegg J. K.; Nitschke J. R. Self-Assembled M_8_L_6_ Cubic Cage That Selectively Encapsulates Large Aromatic Guests. Angew. Chem., Int. Ed. 2011, 50, 3479–3483. 10.1002/anie.201100193. 21394866

[ref128] Tanaka S.; Nakazono T.; Yamauchi K.; Sakai K. Photochemical H_2_ Evolution Catalyzed by Porphyrin-Based Cubic Cages Singly and Doubly Encapsulating PtCl_2_ (4,4’-dimethyl-2,2’-bipyridine). Chem. Lett. 2017, 46, 1573–1575. 10.1246/cl.170692.

[ref129] Tinker L. L.; McDaniel N. D.; Curtin P. N.; Smith C. K.; Ireland M. J.; Bernhard S. Visible Light Induced Catalytic Water Reduction without an Electron Relay. Chem.—Eur. J. 2007, 13, 8726–8732. 10.1002/chem.200700480. 17654456

[ref130] Gotico P.; Herrero C.; Protti S.; Quaranta A.; Sheth S.; Fallahpour R.; Farran R.; Halime Z.; Sircoglou M.; Aukauloo A.; Leibl W. Proton-Controlled Action of an Imidazole as Electron Relay in a Photoredox Triad. Photochem. Photobiol. Sci. 2022, 21, 247–259. 10.1007/s43630-021-00163-2. 34988933

[ref131] Ghosh A. C.; Legrand A.; Rajapaksha R.; Craig G. A.; Sassoye C.; Balázs G.; Farrusseng D.; Furukawa S.; Canivet J.; Wisser F. M. Rhodium-Based Metal-Organic Polyhedra Assemblies for Selective CO_2_ Photoreduction. J. Am. Chem. Soc. 2022, 144, 3626–3636. 10.1021/jacs.1c12631. 35179874

[ref132] Wisser F. M.; Berruyer P.; Cardenas L.; Mohr Y.; Quadrelli E. A.; Lesage A.; Farrusseng D.; Canivet J. Hammett Parameter in Microporous Solids as Macroligands for Heterogenized Photocatalysts. ACS Catal. 2018, 8, 1653–1661. 10.1021/acscatal.7b03998.

[ref133] Jing X.; He C.; Yang Y.; Duan C. A Metal-Organic Tetrahedron as a Redox Vehicle to Encapsulate Organic Dyes for Photocatalytic Proton Reduction. J. Am. Chem. Soc. 2015, 137, 3967–3974. 10.1021/jacs.5b00832. 25738748

[ref134] Wang H.; Li L.; Li X.; He C. Encapsulation of Organic Dyes within an Electron-Deficient Redox Metal-Organic Tetrahedron for Photocatalytic Proton Reduction. Isr. J. Chem. 2019, 59, 273–279. 10.1002/ijch.201800145.

[ref135] Yang L.; Jing X.; He C.; Chang Z.; Duan C. Redox-Active M_8_L_6_ Cubic Hosts with Tetraphenylethylene Faces Encapsulate Organic Dyes for Light-Driven H_2_ Production. Chem.—Eur. J. 2016, 22, 18107–18114. 10.1002/chem.201601447. 27699903

[ref136] Van Arman S. A.; Zimmet A. J.; Murray I. E. A Hantzsch Amido Dihydropyridine as a Transfer Hydrogenation Reagent for α,β-Unsaturated Ketones. J. Org. Chem. 2016, 81, 3528–3532. 10.1021/acs.joc.6b00041. 27083498

[ref137] Chen Q. A.; Gao K.; Duan Y.; Ye Z. S.; Shi L.; Yang Y.; Zhou Y. G. Dihydrophenanthridine: A New and Easily Regenerable NAD(P)H Model for Biomimetic Asymmetric Hydrogenation. J. Am. Chem. Soc. 2012, 134, 2442–2448. 10.1021/ja211684v. 22239152

[ref138] Zhao L.; Wang J.; Wu P.; He C.; Guo X.; Duan C. DHPA-Containing Cobalt-Based Redox Metal-Organic Cyclohelicates as Enzymatic Molecular Flasks for Light-Driven H_2_ Production. Sci. Rep. 2017, 7, 14347 10.1038/s41598-017-14728-8. 29085048 PMC5662590

[ref139] Yang L.; He C.; Liu X.; Zhang J.; Sun H.; Guo H. Supramolecular Photoinduced Electron Transfer between a Redox-Active Hexanuclear Metal-Organic Cylinder and an Encapsulated Ruthenium(II) Complex. Chem.—Eur. J. 2016, 22, 5253–5260. 10.1002/chem.201504975. 26923780

[ref140] Zhao L.; Wei J.; Zhang F.; He C.; Zheng S.; Duan C. Redox-Active Copper Triangles as an Enzymatic Molecular Flask for Light-Driven Hydrogen Production. RSC Adv. 2017, 7, 48989–48993. 10.1039/C7RA09285G.

[ref141] Cai J.; Zhao L.; Wei J.; He C.; Long S.; Duan C. Negatively Charged Metal-Organic Hosts with Cobalt Dithiolene Species: Improving PET Processes for Light-Driven Proton Reduction through Host-Guest Electrostatic Interactions. Chem. Commun. 2019, 55, 8524–8527. 10.1039/C9CC03871J. 31257393

[ref142] Jing X.; Yang Y.; He C.; Chang Z.; Reek J. N. H.; Duan C. Control of Redox Events by Dye Encapsulation Applied to Light-Driven Splitting of Hydrogen Sulfide. Angew. Chem., Int. Ed. 2017, 129, 11921–11925. 10.1002/ange.201704327. 28722265

[ref143] la Cour A.; Findeisen M.; Hazell A.; Hazell R.; Zdobinsky G. Metal(II) N_2_S_2_ Schiff-Base Complexes Incorporating Pyrazole or Isoxazole (M = Ni, Cu or Zn). Spinstates, Racemization Kinetics and Electrochemistry. J. Chem. Soc., Dalton Trans. 1997, 1997, 121–128. 10.1039/a602281b.

[ref144] Yang Y.; Li H.; Jing X.; Wu Y.; Shi Y.; Duan C. Dye-Loaded Metal-Organic Helical Capsules Applied to the Combination of Photocatalytic H_2_S Splitting and Nitroaromatic Hydrogenation. Chem. Commun. 2022, 58, 807–810. 10.1039/D1CC06166F. 34928273

[ref145] Yu H.; He C.; Xu J.; Duan C.; Reek J. N. H. Metal-Organic Redox Vehicles to Encapsulate Organic Dyes for Photocatalytic Protons and Carbon Dioxide. Inorg. Chem. Front. 2016, 3, 1256–1263. 10.1039/C6QI00211K.

[ref146] Nurttila S. S.; Becker R.; Hessels J.; Woutersen S.; Reek J. N. H. Photocatalytic Hydrogen Evolution by a Synthetic [FeFe] Hydrogenase Mimic Encapsulated in a Porphyrin Cage. Chem.—Eur. J. 2018, 24, 16395–16406. 10.1002/chem.201803351. 30117602 PMC6282596

[ref147] He C.; Wang J.; Zhao L.; Liu T.; Zhang J.; Duan C. A Photoactive Basket-like Metal-Organic Tetragon Worked as an Enzymatic Molecular Flask for Light Driven H_2_ Production. Chem. Commun. 2013, 49, 627–629. 10.1039/C2CC37853A. 23223366

[ref148] Sun M.; Wang Q. Q.; Qin C.; Sun C. Y.; Wang X. L.; Su Z. M. An Amine-Functionalized Zirconium Metal-Organic Polyhedron Photocatalyst with High Visible-Light Activity for Hydrogen Production. Chem.—Eur. J. 2019, 25, 2824–2830. 10.1002/chem.201805663. 30575148

[ref149] Sun M.; Sun C.; Wang X.; Su Z. Promoting Visible-Light-Driven Hydrogen Production of a Zirconium-Based Metal-Organic Polyhedron Decorated by Platinum Nanoparticles with Different Spatial Locations. Catal. Commun. 2020, 137, 105930 10.1016/j.catcom.2020.105930.

[ref150] Chen S.; Li K.; Zhao F.; Zhang L.; Pan M.; Fan Y.-Z.; Guo J.; Shi J.; Su C.-Y. A Metal-Organic Cage Incorporating Multiple Light Harvesting and Catalytic Centres for Photochemical Hydrogen Production. Nat. Commun. 2016, 7, 13169 10.1038/ncomms13169. 27827376 PMC5105156

[ref151] Bendikov M.; Wudl F.; Perepichka D. F. Tetrathiafulvalenes, Oligoacenenes, and Their Buckminsterfullerene Derivatives: The Brick and Mortar of Organic Electronics. Chem. Rev. 2004, 104, 4891–4945. 10.1021/cr030666m. 15535637

[ref152] Wu K.; Li K.; Chen S.; Hou Y.; Lu Y.; Wang J.; Wei M.; Pan M.; Su C. The Redox Coupling Effect in a Photocatalytic Ru^II^-Pd ^II^ Cage with TTF Guest as Electron Relay Mediator for Visible-Light Hydrogen-Evolving Promotion. Angew. Chem., Int. Ed. 2020, 59, 2639–2643. 10.1002/anie.201913303. 31758622

[ref153] Yoshizawa M.; Kumazawa K.; Fujita M. Room-Temperature and Solution-State Observation of the Mixed-Valence Cation Radical Dimer of Tetrathiafulvalene, [(TTF)2]^+•^, within a Self-Assembled Cage. J. Am. Chem. Soc. 2005, 127, 13456–13457. 10.1021/ja053508g. 16190683

[ref154] Qi X.; Zhong R.; Chen M.; Sun C.; You S.; Gu J.; Shan G.; Cui D.; Wang X.; Su Z. Single Metal-Organic Cage Decorated with an Ir(III) Complex for CO_2_ Photoreduction. ACS Catal. 2021, 11, 7241–7248. 10.1021/acscatal.1c01974.

[ref155] Zheng Y. R.; Zhao Z.; Wang M.; Ghosh K.; Pollock J. B.; Cook T. R.; Stang P. J. A Facile Approach toward Multicomponent Supramolecular Structures: Selective Self-Assembly via Charge Separation. J. Am. Chem. Soc. 2010, 132, 16873–16882. 10.1021/ja106251f. 21053935 PMC3016897

[ref156] Zhu H.; Li Q.; Shi B.; Ge F.; Liu Y.; Mao Z.; Zhu H.; Wang S.; Yu G.; Huang F.; Stang P. J. Dual-Emissive Platinum(II) Metallacage with a Sensitive Oxygen Response for Imaging of Hypoxia and Imaging-Guided Chemotherapy. Angew. Chem., Int. Ed. 2020, 59, 20208–20214. 10.1002/anie.202009442. 32710650

[ref157] Melchiorre P. Introduction: Photochemical Catalytic Processes. Chem. Rev. 2022, 122, 1483–1484. 10.1021/acs.chemrev.1c00993. 35078320

[ref158] Kwon K.; Simons R. T.; Nandakumar M.; Roizen J. L. Strategies to Generate Nitrogen-Centered Radicals That May Rely on Photoredox Catalysis: Development in Reaction Methodology and Applications in Organic Synthesis. Chem. Rev. 2022, 122, 2353–2428. 10.1021/acs.chemrev.1c00444. 34623809 PMC8792374

[ref159] Chang L.; An Q.; Duan L.; Feng K.; Zuo Z. Alkoxy Radicals See the Light: New Paradigms of Photochemical Synthesis. Chem. Rev. 2022, 122, 2429–2486. 10.1021/acs.chemrev.1c00256. 34613698

[ref160] Allen A. R.; Noten E. A.; Stephenson C. R. J. Aryl Transfer Strategies Mediated by Photoinduced Electron Transfer. Chem. Rev. 2022, 122, 2695–2751. 10.1021/acs.chemrev.1c00388. 34672526 PMC9272681

[ref161] Juliá F.; Constantin T.; Leonori D. Applications of Halogen-Atom Transfer (XAT) for the Generation of Carbon Radicals in Synthetic Photochemistry and Photocatalysis. Chem. Rev. 2022, 122, 2292–2352. 10.1021/acs.chemrev.1c00558. 34882396

[ref162] Cheung K. P. S.; Sarkar S.; Gevorgyan V. Visible Light-Induced Transition Metal Catalysis. Chem. Rev. 2022, 122, 1543–1625. 10.1021/acs.chemrev.1c00403. 34623151 PMC9017709

[ref163] Tay N. E. S.; Lehnherr D.; Rovis T. Photons or Electrons? A Critical Comparison of Electrochemistry and Photoredox Catalysis for Organic Synthesis. Chem. Rev. 2022, 122, 2487–2649. 10.1021/acs.chemrev.1c00384. 34751568 PMC10021920

[ref164] Murray P. R. D.; Cox J. H.; Chiappini N. D.; Roos C. B.; McLoughlin E. A.; Hejna B. G.; Nguyen S. T.; Ripberger H. H.; Ganley J. M.; Tsui E.; et al. Photochemical and Electrochemical Applications of Proton-Coupled Electron Transfer in Organic Synthesis. Chem. Rev. 2022, 122, 2017–2291. 10.1021/acs.chemrev.1c00374. 34813277 PMC8796287

[ref165] Genzink M. J.; Kidd J. B.; Swords W. B.; Yoon T. P. Chiral Photocatalyst Structures in Asymmetric Photochemical Synthesis. Chem. Rev. 2022, 122, 1654–1716. 10.1021/acs.chemrev.1c00467. 34606251 PMC8792375

[ref166] Capaldo L.; Ravelli D.; Fagnoni M. Direct Photocatalyzed Hydrogen Atom Transfer (HAT) for Aliphatic C-H Bonds Elaboration. Chem. Rev. 2022, 122, 1875–1924. 10.1021/acs.chemrev.1c00263. 34355884 PMC8796199

[ref167] Neveselý T.; Wienhold M.; Molloy J. J.; Gilmour R. Advances in the E → Z Isomerization of Alkenes Using Small Molecule Photocatalysts. Chem. Rev. 2022, 122, 2650–2694. 10.1021/acs.chemrev.1c00324. 34449198

[ref168] Corbin D. A.; Miyake G. M. Photoinduced Organocatalyzed Atom Transfer Radical Polymerization (O-ATRP): Precision Polymer Synthesis Using Organic Photoredox Catalysis. Chem. Rev. 2022, 122, 1830–1874. 10.1021/acs.chemrev.1c00603. 34842426 PMC9815475

[ref169] Buglioni L.; Raymenants F.; Slattery A.; Zondag S. D. A.; Noël T. Technological Innovations in Photochemistry for Organic Synthesis: Flow Chemistry, High-Throughput Experimentation, Scale-up, and Photoelectrochemistry. Chem. Rev. 2022, 122, 2752–2906. 10.1021/acs.chemrev.1c00332. 34375082 PMC8796205

[ref170] Glaser F.; Wenger O. S. Recent Progress in the Development of Transition-Metal Based Photoredox Catalysts. Coord. Chem. Rev. 2020, 405, 213129 10.1016/j.ccr.2019.213129.

[ref171] Nicewicz D. A.; MacMillan D. W. C. Merging Photoredox Catalysis With Organocatalysis: The Direct Asymmetric Alkylation of Aldehydes. Science 2008, 322, 77–80. 10.1126/science.1161976. 18772399 PMC2723798

[ref172] Barham J. P.; König B. Synthetic Photoelectrochemistry. Angew. Chem., Int. Ed. 2020, 59, 11732–11747. 10.1002/anie.201913767. PMC7383880 31805216

[ref173] Özgen F. F.; Runda M. E.; Schmidt S. Photo-Biocatalytic Cascades: Combining Chemical and Enzymatic Transformations Fueled by Light. ChemBioChem. 2021, 22, 790–806. 10.1002/cbic.202000587. 32961020 PMC7983893

[ref174] Lang X.; Zhao J.; Chen X. Cooperative Photoredox Catalysis. Chem. Soc. Rev. 2016, 45, 3026–3038. 10.1039/C5CS00659G. 27094803

[ref175] Busschaert N.; Caltagirone C.; van Rossom W.; Gale P. A. Applications of Supramolecular Anion Recognition. Chem. Rev. 2015, 115, 8038–8155. 10.1021/acs.chemrev.5b00099. 25996028

[ref176] Ma X.; Zhao Y. Biomedical Applications of Supramolecular Systems Based on Host-Guest Interactions. Chem. Rev. 2015, 115, 7794–7839. 10.1021/cr500392w. 25415447

[ref177] Fujita M.; Oguro D.; Miyazawa M.; Oka H.; Yamaguchi K.; Ogura K. Self-Assembly of Ten Molecules into Nanometre-Sized Organic Host Frameworks. Nature 1995, 378, 469–471. 10.1038/378469a0.

[ref178] Furusawa T.; Kawano M.; Fujita M. The Confined Cavity of a Coordination Cage Suppresses the Photocleavage of α-Diketones to Give Cyclization Products through Kinetically Unfavorable Pathways. Angew. Chem., Int. Ed. 2007, 46, 5717–5719. 10.1002/anie.200701250. 17591741

[ref179] Yamaguchi T.; Fujita M. Highly Selective Photomediated 1,4-Radical Addition to *o*-Quinones Controlled by a Self-Assembled Cage. Angew. Chem., Int. Ed. 2008, 47, 2067–2069. 10.1002/anie.200705139. 18247442

[ref180] Yuan J.; Wei Z.; Shen K.; Yang Y.; Liu M.; Jing X.; Duan C. Encapsulating Electron-Deficient Dyes into Metal-Organic Capsules to Achieve High Reduction Potentials. Dalton Trans. 2022, 51, 10860–10865. 10.1039/D2DT01166B. 35781472

[ref181] Studer A.; Curran D. P. Organocatalysis and C-H Activation Meet Radical- and Electron-Transfer Reactions. Angew. Chem., Int. Ed. 2011, 50, 5018–5022. 10.1002/anie.201101597. 21523871

[ref182] Herndon W. C. The Theory of Cycloaddition Reactions. Chem. Rev. 1972, 72, 157–179. 10.1021/cr60276a003.

[ref183] Yoshizawa M.; Takeyama Y.; Kusukawa T.; Fujita M. Cavity-Directed, Highly Stereoselective [2 + 2] Photodimerization of Olefins within Self-Assembled Coordination Cages. Angew. Chem. Int. Ed 2002, 41, 1347–1349. 10.1002/1521-3773(20020415)41:8<1347::AID-ANIE1347>3.0.CO;2-X. 19750759

[ref184] Yoshizawa M.; Takeyama Y.; Okano T.; Fujita M. Cavity-Directed Synthesis within a Self-Assembled Coordination Cage: Highly Selective [2 + 2] Cross-Photodimerization of Olefins. J. Am. Chem. Soc. 2003, 125, 3243–3247. 10.1021/ja020718+. 12630879

[ref185] Nishioka Y.; Yamaguchi T.; Kawano M.; Fujita M. Asymmetric [2 + 2] Olefin Cross Photoaddition in a Self-Assembled Host with Remote Chiral Auxiliaries. J. Am. Chem. Soc. 2008, 130, 8160–8161. 10.1021/ja802818t. 18540605

[ref186] Crini G. Review: A History of Cyclodextrins. Chem. Rev. 2014, 114, 10940–10975. 10.1021/cr500081p. 25247843

[ref187] Gera R.; Dasgupta J. Photochemistry Using a Host-Guest Charge Transfer Paradigm: DMABN as a Dynamical Probe of Ground and Excited States. Phys. Chem. Chem. Phys. 2021, 23, 9280–9284. 10.1039/D1CP00370D. 33885087

[ref188] Gera R.; Das A.; Jha A.; Dasgupta J. Light-Induced Proton-Coupled Electron Transfer inside a Nanocage. J. Am. Chem. Soc. 2014, 136, 15909–15912. 10.1021/ja509761a. 25333866

[ref189] Das A.; Mandal I.; Venkatramani R.; Dasgupta J. Ultrafast Photoactivation of C-H Bonds inside Water-Soluble Nanocages. Sci. Adv. 2019, 5, eaav4806 10.1126/sciadv.aav4806. 30801018 PMC6386559

[ref190] Cai L. X.; Li S. C.; Yan D. N.; Zhou L. P.; Guo F.; Sun Q. F. Water-Soluble Redox-Active Cage Hosting Polyoxometalates for Selective Desulfurization Catalysis. J. Am. Chem. Soc. 2018, 140, 4869–4876. 10.1021/jacs.8b00394. 29534562

[ref191] Yan D. N.; Cai L. X.; Cheng P. M.; Hu S. J.; Zhou L. P.; Sun Q. F. Photooxidase Mimicking with Adaptive Coordination Molecular Capsules. J. Am. Chem. Soc. 2021, 143, 16087–16094. 10.1021/jacs.1c06390. 34553600

[ref192] Yan D. N.; Cai L. X.; Hu S. J.; Zhou Y. F.; Zhou L. P.; Sun Q. F. An Organo-Palladium Host Built from a Dynamic Macrocyclic Ligand: Adaptive Self-Assembly, Induce-Fit Guest Binding, and Catalysis. Angew. Chem., Int. Ed. 2022, 61, e202209879 10.1002/anie.202209879. 36036434

[ref193] Gu J.; Chen W.; Shan G. G.; Li G.; Sun C.; Wang X. L.; Su Z. The Roles of Polyoxometalates in Photocatalytic Reduction of Carbon Dioxide. Mater. Today Energy 2021, 21, 100760 10.1016/j.mtener.2021.100760.

[ref194] Sivakumar R.; Thomas J.; Yoon M. Polyoxometalate-Based Molecular/Nano Composites: Advances in Environmental Remediation by Photocatalysis and Biomimetic Approaches to Solar Energy Conversion. J. Photochem. Photobiol. C 2012, 13, 277–298. 10.1016/j.jphotochemrev.2012.08.001.

[ref195] Meza L. R.; Das S.; Greer J. R. Strong, Lightweight, and Recoverable Three-Dimensional Ceramic Nanolattices. Science 2014, 345, 1322–1326. 10.1126/science.1255908. 25214624

[ref196] Lin H. Y.; Zhou L. Y.; Xu L. Photocatalysis in Supramolecular Fluorescent Metallacycles and Metallacages. Chem.—Asian J. 2021, 16, 3805–3816. 10.1002/asia.202100942. 34529337

[ref197] Yoshizawa M.; Miyagi S.; Kawano M.; Ishiguro K.; Fujita M. Alkane Oxidation via Photochemical Excitation of a Self-Assembled Molecular Cage. J. Am. Chem. Soc. 2004, 126, 9172–9173. 10.1021/ja047612u. 15281793

[ref198] Murase T.; Takezawa H.; Fujita M. Photo-Driven Anti-Markovnikov Alkyne Hydration in Self-Assembled Hollow Complexes. Chem. Commun. 2011, 47, 10960–10962. 10.1039/c1cc14523a. 21904772

[ref199] Murase T.; Nishijima Y.; Fujita M. Unusual Photoreaction of Triquinacene within Self-Assembled Hosts. Chem.—Asian J. 2012, 7, 826–829. 10.1002/asia.201101005. 22311687

[ref200] Cullen W.; Takezawa H.; Fujita M. Demethylenation of Cyclopropanes via Photoinduced Guest-to-Host Electron Transfer in an M_6_L_4_ Cage. Angew. Chem., Int. Ed. 2019, 58, 9171–9173. 10.1002/anie.201904752. 31066186

[ref201] Griefs P. Vorläufige Notiz Über Die Einwirkung von Salpetriger Säure Auf Amidinitro- Und Aminitrophenylsäure. Annalen der Chemie und Pharmacie 1858, 106, 123–125. 10.1002/jlac.18581060114.

[ref202] Wang Z. Griess Diazotization. In Comprehensive Organic Name Reactions and Reagents; John Wiley & Sons: Hoboken, NJ, 2010; pp 1267–1270.

[ref203] Dalton D. M.; Ellis S. R.; Nichols E. M.; Mathies R. A.; Toste F. D.; Bergman R. G.; Raymond K. N. Supramolecular Ga_4_L_6_^12–^ Cage Photosensitizes 1,3-Rearrangement of Encapsulated Guest via Photoinduced Electron Transfer. J. Am. Chem. Soc. 2015, 137, 10128–10131. 10.1021/jacs.5b06317. 26256754

[ref204] Schmidt M.; Esser B. Cavity-Promotion by Pillar[5]Arenes Expedites Organic Photoredox-Catalysed Reductive Dehalogenations. Chem. Commun. 2021, 57, 9582–9585. 10.1039/D1CC03221F. 34546245

[ref205] Discekici E. H.; Treat N. J.; Poelma S. O.; Mattson K. M.; Hudson Z. M.; Luo Y.; Hawker C. J.; de Alaniz J. R. A Highly Reducing Metal-Free Photoredox Catalyst: Design and Application in Radical Dehalogenations. Chem. Commun. 2015, 51, 11705–11708. 10.1039/C5CC04677G. 26104847

[ref206] Qiang H.; Chen T.; Wang Z.; Li W.; Guo Y.; Yang J.; Jia X.; Yang H.; Hu W.; Wen K. Pillar[5]Arene Based Conjugated Macrocycle Polymers with Unique Photocatalytic Selectivity. Chin. Chem. Lett. 2020, 31, 3225–3229. 10.1016/j.cclet.2020.04.020.

[ref207] Guo J.; Xu Y. W.; Li K.; Xiao L. M.; Chen S.; Wu K.; Chen X. D.; Fan Y. Z.; Liu J. M.; Su C. Y. Regio- and Enantioselective Photodimerization within the Confined Space of a Homochiral Ruthenium/Palladium Heterometallic Coordination Cage. Angew. Chem., Int. Ed. 2017, 56, 3852–3856. 10.1002/anie.201611875. 28247533

[ref208] Wang J. S.; Wu K.; Yin C.; Li K.; Huang Y.; Ruan J.; Feng X.; Hu P.; Su C. Y. Cage-Confined Photocatalysis for Wide-Scope Unusually Selective [2 + 2] Cycloaddition through Visible-Light Triplet Sensitization. Nat. Commun. 2020, 11, 4675 10.1038/s41467-020-18487-5. 32938933 PMC7494878

[ref209] Guo J.; Fan Y. Z.; Lu Y. L.; Zheng S. P.; Su C. Y. Visible-Light Photocatalysis of Asymmetric [2 + 2] Cycloaddition in Cage-Confined Nanospace Merging Chirality with Triplet-State Photosensitization. Angew. Chem., Int. Ed. 2020, 59, 8661–8669. 10.1002/anie.201916722. 32011801

[ref210] Jin Y.; Jiang H.; Tang X.; Zhang W.; Liu Y.; Cui Y. Coordination-Driven Self-Assembly of Anthraquinone-Based Metal-Organic Cages for Photocatalytic Selective [2 + 2] Cycloaddition. Dalton Trans. 2021, 50, 8533–8539. 10.1039/D1DT00652E. 34075985

[ref211] Li X.; Wu J.; Chen L.; Zhong X.; He C.; Zhang R.; Duan C. Engineering an Iridium-Containing Metal-Organic Molecular Capsule for Induced-Fit Geometrical Conversion and Dual Catalysis. Chem. Commun. 2016, 52, 9628–9631. 10.1039/C6CC04647A. 27320443

[ref212] Sunohara H.; Koyamada K.; Takezawa H.; Fujita M. An Ir_3_L_2_ complex with Anion Binding Pockets: Photocatalytic *E-Z* isomerization via molecular Recognition. Chem. Commun. 2021, 57, 9300–9302. 10.1039/D1CC03620C. 34519311

[ref213] Kumar A.; Saha R.; Mukherjee P. S. Self-Assembled Metallasupramolecular Cages towards Light Harvesting Systems for Oxidative Cyclization. Chem. Sci. 2021, 12, 5319–5329. 10.1039/D1SC00097G. 34163765 PMC8179592

[ref214] Hong Y.; Lam J. W. Y.; Tang B. Z. Aggregation-Induced Emission. Chem. Soc. Rev. 2011, 40, 5361–5388. 10.1039/c1cs15113d. 21799992

[ref215] Mei J.; Hong Y.; Lam J. W. Y.; Qin A.; Tang Y.; Tang B. Z. Aggregation-Induced Emission: The Whole Is More Brilliant than the Parts. Adv. Mater. 2014, 26, 5429–5479. 10.1002/adma.201401356. 24975272

[ref216] Mei J.; Leung N. L. C.; Kwok R. T. K.; Lam J. W. Y.; Tang B. Z. Aggregation-Induced Emission: Together We Shine, United We Soar!. Chem. Rev. 2015, 115, 11718–11940. 10.1021/acs.chemrev.5b00263. 26492387

[ref217] Zhang B.; Reek J. N. H. Supramolecular Strategies for the Recycling of Homogeneous Catalysts. Chem.—Asian J. 2021, 16, 3851–3863. 10.1002/asia.202100968. 34606169 PMC9297887

[ref218] Levi S. A.; Guatteri P.; van Veggel F. C. J. M.; Vancso G. J.; Dalcanale E.; Reinhoudt D. N. Direct Observation of Surface-Controlled Self-Assembly of Coordination Cages by Using AFM as a Molecular Ruler. Angew. Chem., Int. Ed. 2001, 40, 1892–1896. 10.1002/1521-3773(20010518)40:10<1892::AID-ANIE1892>3.0.CO;2-J. 11385664

[ref219] Menozzi E.; Pinalli R.; Speets E. A.; Ravoo B. J.; Dalcanale E.; Reinhoudt D. N. Surface-Confined Single Molecules: Assembly and Disassembly of Nanosize Coordination Cages on Gold (111). Chem.—Eur. J. 2004, 10, 2199–2206. 10.1002/chem.200305570. 15112208

[ref220] Ryan H. P.; Haynes C. J. E.; Smith A.; Grommet A. B.; Nitschke J. R. Guest Encapsulation within Surface-Adsorbed Self-Assembled Cages. Adv. Mater. 2021, 33, 2004192 10.1002/adma.202004192. 33236814

[ref221] Miyamura H.; Bergman R. G.; Raymond K. N.; Toste F. D. Heterogeneous Supramolecular Catalysis through Immobilization of Anionic M_4_L_6_ Assemblies on Cationic Polymers. J. Am. Chem. Soc. 2020, 142, 19327–19338. 10.1021/jacs.0c09556. 33136406

[ref222] McCarthy B. D.; Beiler A. M.; Johnson B. A.; Liseev T.; Castner A. T.; Ott S. Analysis of Electrocatalytic Metal-Organic Frameworks. Coord. Chem. Rev. 2020, 406, 213137 10.1016/j.ccr.2019.213137. 32499663 PMC7272229

[ref223] Vericat C.; Vela M. E.; Benitez G.; Carro P.; Salvarezza R. C. Self-Assembled Monolayers of Thiols and Dithiols on Gold: New Challenges for a Well-Known System. Chem. Soc. Rev. 2010, 39, 1805–1834. 10.1039/b907301a. 20419220

[ref224] Busi M.; Laurenti M.; Condorelli G. G.; Motta A.; Favazza M.; Fragalà I. L.; Montalti M.; Prodi L.; Dalcanale E. Self-Assembly of Nanosize Coordination Cages on Si(100) Surfaces. Chem.—Eur. J. 2007, 13, 6891–6898. 10.1002/chem.200700496. 17535002

[ref225] Condorelli G. G.; Motta A.; Favazza M.; Fragalà I. L.; Busi M.; Menozzi E.; Dalcanale E.; Cristofolini L. Grafting Cavitands on the Si(100) Surface. Langmuir 2006, 22, 11126–11133. 10.1021/la060682p. 17154593

